# Molecular dynamic staircases: all-carbon axial chiral “Geländer” structures†
†Electronic supplementary information (ESI) available: Experimental procedures, high-resolution ESI-MS and EI-MS, NMR, UV-Vis and CD spectroscopy, HPLC chromatograms, data of kinetic experiment and crystal refinement details. CCDC 1811809 and 1811810. For ESI and crystallographic data in CIF or other electronic format see DOI: 10.1039/c8sc01707g


**DOI:** 10.1039/c8sc01707g

**Published:** 2018-06-05

**Authors:** Rajesh Mannancherry, Michel Rickhaus, Daniel Häussinger, Alessandro Prescimone, Marcel Mayor

**Affiliations:** a Department of Chemistry , University of Basel , St. Johanns-Ring 19 , 4056 Basel , Switzerland . Email: marcel.mayor@unibas.ch; b Institute for Nanotechnology (INT) , Karlsruhe Institute of Technology (KIT) , P. O. Box 3640 , 76021 Karlsruhe , Germany; c Lehn Institute of Functional Materials (LFM) , School of Chemistry , Sun Yat-Sen University (SYSU) , Guangzhou 510275 , China

## Abstract

Herein, the syntheses of two tightly packed all-carbon “Geländer” molecules with axial chirality are described. Motivated by our previous results, we further reduced the bridge length by excluding the heteroatoms. The absolute configuration was determined by comparison of the measured and calculated circular dichroism (CD) spectra and the thermodynamic stability was determined by dynamic high-performance liquid chromatography (HPLC) and CD analysis. The cyclophanes were fully characterized by CD measurements, X-ray diffraction (XRD) analysis, NMR, UV-Vis and high resolution mass spectrometry (HRMS). Our novel all-carbon macrocycle is the most stable Geländer system reported so far.

## Abstract 2

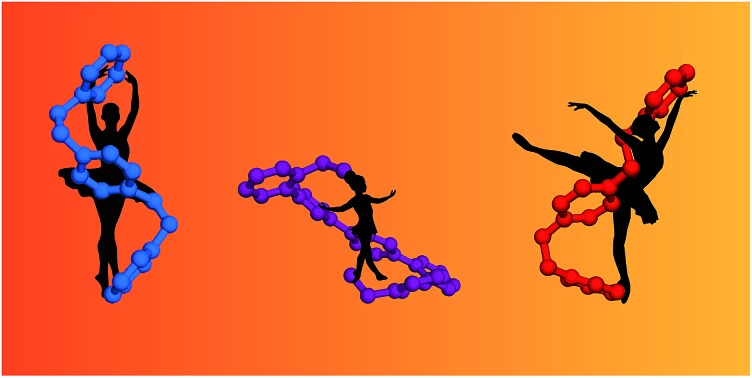
Expanding on previous Geländer models, we designed and characterized two chiral all-carbon Geländer molecules with enhanced thermodynamic stabilities.

## Introduction

Molecules with helical structure have fascinated chemists for many years. Spiral staircases,^1^–^10^ propellers,^11^,^12^ and screws^13^ are representative examples of helical structures that have attracted the attention of designers, architects and researchers. Chiral polycyclic aromatic compounds (PACs) have been of particular interest to fundamental researchers and material scientists since scientists became aware of correlations between molecular structure and physical properties.^14^,^15^ PACs have gained strong interest from both chemists and physicists due to their structural beauty, stability and exceptional optical and electronic properties for material applications. In principle, molecules with such an unique topology can be synthesized through three different approaches:^16^ first, through stabilizing intramolecular noncovalent interactions. One classical example is the α-helix in proteins, which is stabilized within a linear peptide strand by intramolecular hydrogen bonds between the amide groups of amino acids *i* and *i* + 4.^17^ Second, through stabilizing intermolecular noncovalent interactions by two or more molecules. The most famous example is the DNA helix, in which two complementary base strands are stabilized by intermolecular hydrogen bonds forming the well-known double helical conformation.^18^ The third approach to design helical structures is formed by steric effects or by length mismatch of parallel strands in rigid molecules. John A. Gladysz and co-workers impressively demonstrated the syntheses of diplatinum sp-carbon chain complexes with a double-helical arrangement.^19^ The metal–organic linear axis is wrapped by two longer alkyl-chains, which adapt a double helical arrangement resembling the double helical motif of DNA.

**Fig. 1:**
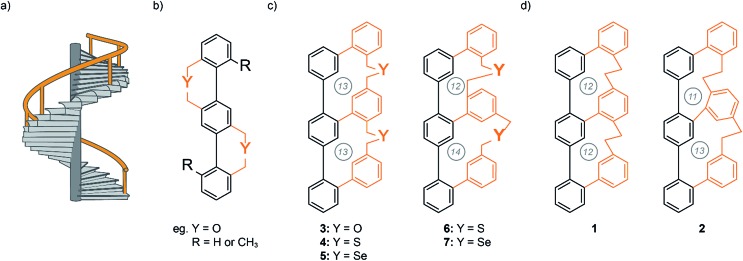
(a) Concept of a helical staircase with a banister (orange). (b) Vögtle's first terphenylene Geländer oligomer design.^1^,^2^ (c) Schematic illustration of the achieved Geländer molecules **3–7** with different heteroatoms in the bridging unit.^7^,^9^ (d) Schematic illustration of the all-carbon Geländer macrocycle **1** and **2**. The circled numbers correspond to the number of atoms in each macrocycle.

Another design of the later method, is the well-studied helical Geländer molecule. Geländer is the German word for the banister of a spiral staircase (Fig. 1a) and was first described by Vögtle and co-workers at the end of the 1990s.^1^,^2^ These are *ortho*-bridged terphenylenes in which the helical arrangement results from steric interactions between the bulky substituents *ortho* to the biaryl axis (Fig. 1b). Whereas helicenes and their derivatives consist of aromatic rings that are arranged perpendicularly to the propagation axis of the helix (helical chirality), the orientation of Geländer oligomers are along the propagation axis, which has to be an integral part of the molecular structure (axial chirality). However, due to the symmetry of Vögtle's terphenyl Geländer oligomers, each biphenyl bond is identical and can adopt either an *M* or *P* conformation. The chiral appearance of the molecule becomes a statistical process with 50% probability of the achiral *meso* conformation (*M*,*P*/*P*,*M*) and only 25% for each enantiomer with *P*,*P* and *M*,*M* conformation.

Inspired by Vögtle's Geländer oligomers we developed a ladder-like system, which solely forms either the *M* or *P* conformation.^7^–^9^ The terphenyl backbone is connected to a longer oligophenylene strand with individual linkages to each benzene unit of the backbone. The system can only overcome the length mismatch by wrapping the longer rail around the backbone. Thereby the chiral information is communicated across the entire banister part inducing a helical arrangement. We synthesized three Geländer oligomers with different chalcogens (oxygen, sulfur and selenium) in each bridging unit (Fig. 1c). Investigations by CD and XRD analysis demonstrated the implementation of the concept. Variable temperature CD spectroscopy allowed us to analyze the stereodynamic behavior and to determine the racemization barriers of the Geländer macrocycles **3–7**.^7^,^9^ The very comparable stereodynamic behaviors of the different Geländer model compounds **3–7** points at comparable stiffness features of the bridges, and it seems that a longer bond-length to a heavier chalcogen atom is compensated by the increased softness of the chalcogen ether bridge. To overcome the diversity imposed by various chalcogen atoms, we became interested in bridging structures comprising exclusively carbon atoms.

Motivated by these previously achieved results, we present herein a new synthetic strategy for the two all-carbon polycyclic ladder systems **1** and **2**, which are structural isomers that only differ in the sizes of the formed macrocycles (Fig. 1d). In this new design, the two oligomers with mismatch in the spatial requirement of their subunit are an oligo-phenyl and an oligo-phenylene-ethanylene structure, which no longer comprises chalcogen atoms. We were particular curious to investigate the effect of the tighter ethyl bridge compared with the benzylic chalcogen ether linkers on the intramolecular wrapping of both oligomer strands. Here, we report the syntheses of the members of this new family of all-carbon Geländer-type structures together with their full characterization. Furthermore, dynamic features of these helical structures were investigated by dynamic CD and HPLC analysis.

## Results and discussion

**Scheme 1:**
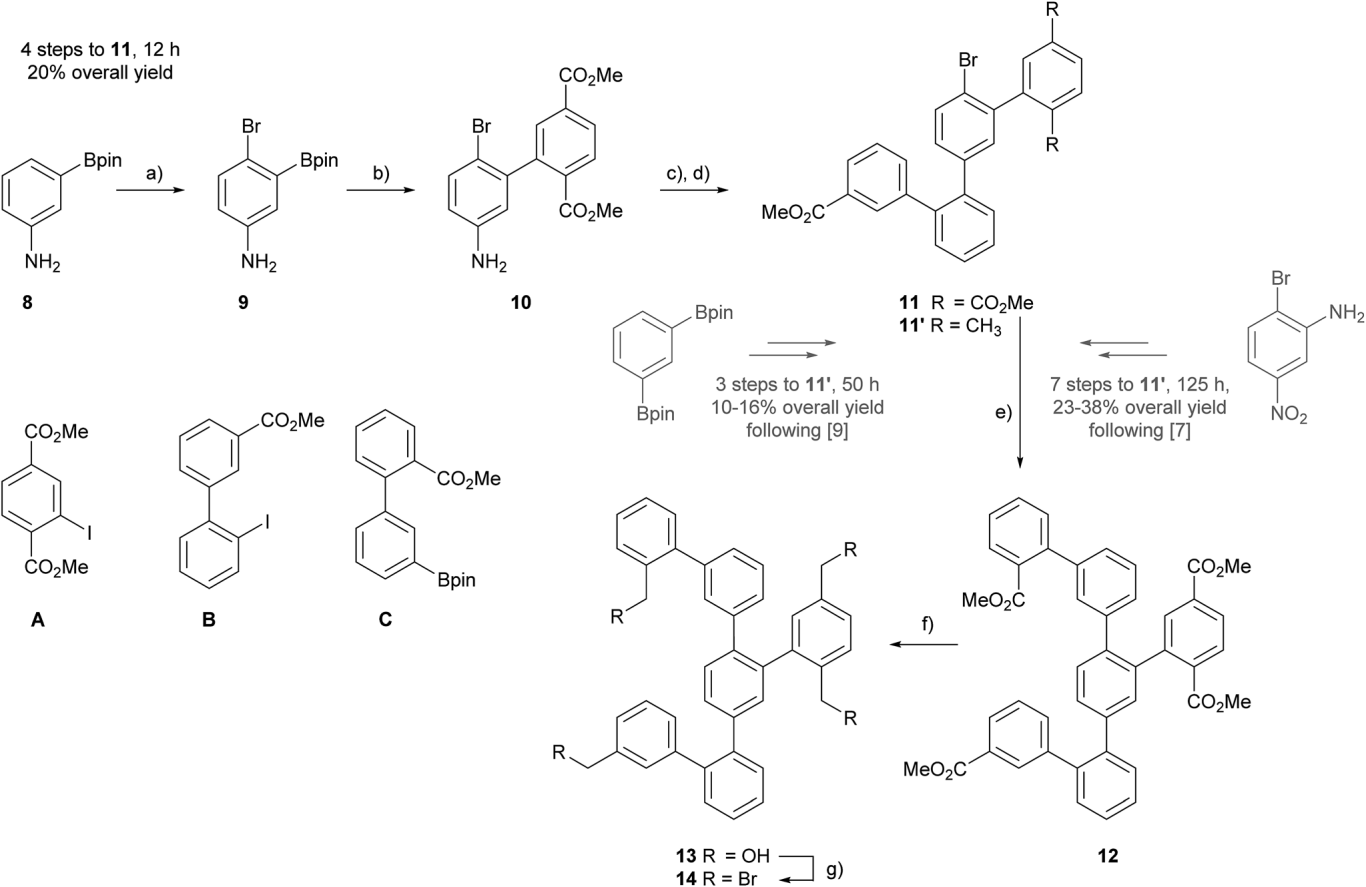
Synthesis of precursor **14**: (a) NBS, DMF, –15 °C, 1 h, 93%. (b) **A**, Pd(PPh_3_)_2_Cl_2_, K_2_CO_3_, 1,4-dioxane/MeOH (4 : 1), 60 °C, 1–2 h, 81%. (c) B_2_pin_2_, BPO, *t*-BuONO, MeCN, 81 °C, 1–2 h. (d) **B**, Pd(PPh_3_)_2_Cl_2_, K_2_CO_3_, 1,4-dioxane/MeOH (4 : 1), 60 °C, 7 h, 31% over 2 steps. (e) Sphos Pd G2, K_2_CO_3_, Tol/H_2_O (4 : 1), 110 °C, 4 d, 69%. (f) DIBAL-H, DCM, RT, 10 min, 93%. (g) PBr_3_, DCM, RT, 30 min, 74%. Bpin = 4,4,5,5-tetramethyl-1,3,2-dioxaborolane, BPO = benzoyl peroxide, SPhos Pd G2 = chloro(2-dicyclohexylphosphino-2′,6′-dimethoxy-1,1′-biphenyl)[2-(2′-amino-1,1′-biphenyl)]palladium(ii).

For the assembly of the all carbon Geländer-type structure a *Wurtz*-type coupling with the precursor **14** was considered. The fourfold benzylic bromide **14** has already been used as precursor for the assembly of the Geländer model compounds with sulfide- and selenide-bridges and thus, we already reported a successful synthesis.^9^ Here, we report an alternative approach making **14** accessible in shorter time and larger amounts. So far, all our assembly strategies were based on the quarterphenyl intermediate **11′** comprising a xylene subunit. As displayed in Scheme 1, the fourfold functionalized structure **11′** was obtained either starting from 2-bromo-5-nitroaniline in seven steps with an over-all yield up to 38%,^7^,^8^ or alternatively from 1,3-bis(4,4,5,5-tetramethyl-1,3,2-dioxaborolan-2-yl)benzene in only three steps but with a mediocre over-all yield of 11–16%.^9^

The targeted intermediate **14** comprises four times the same functional group and thus, using in all four positions the methyl benzoate as masked benzylic bromide seemed reasonable, as suitable methyl benzoate building blocks are easy accessible from inexpensive commercially available precursors. Thus, the assembly of the new quarterphenyl intermediate **11** started from 3-(4,4,5,5-tetramethyl-1,3,2-dioxaborolan-2-yl)aniline **8**, which already comprises two functional groups for subsequent cross-coupling reactions.

Applying an adapted protocol from Kamei and co-workers allowed to introduce a bromine as third functional group.^20^ Treating **8** at low temperature with NBS in DMF provided the brominated compound **9** in excellent 93% isolated yield after column chromatography (CC) as brown oil. The combination of the strong *para*-directing amino group and the *ortho*-directing boronic acid substituent result in perfect regiocontrol of the bromination reaction reflected in the excellent yield of **9**. The trifold functionalized intermediate **9** has the perfect substitution pattern for the central phenyl subunit of the terphenyl backbone. Its bromine and boronic acid ester substituents can be addressed by *Suzuki*-type coupling reactions, while the amine functional group can be considered as pre-stage of an additional boronic acid ester.^21^ The considerably higher reactivity of iodoaryls in Pd-catalyzed coupling reactions compared with bromine derivatives enables to use **9** as boronic acid derivative, in spite the fact that its bromine could react as coupling partner as well. Thus, treating **9** and dimethyl 2-iodoterephthalate (**A**) in *Suzuki*–*Miyaura* cross-coupling conditions not exceeding 60 °C gave the fourfold functionalized biphenyl derivate **10**, which was isolated as light orange oil in 81% yield after CC.

As the bromine substituent perfectly tolerated these reaction conditions without reacting itself, a similar strategy was considered a second time. Inspired by the protocol from Wang and co-workers, the amine of **10** was converted into an arylboronic pinacol ester and without further purification engaged as crude product in a subsequent cross-coupling reaction.^21^ The aniline derivative **10** was treated with one equivalent of bis(pinacolato)diboron (B_2_pin_2_), a slight excess (1.5 eq.) of *tert*-butyl nitrite (*t*-BuONO) in the presence of 2 mol% BPO in refluxing acetonitrile (MeCN) for 1 hour. Due to the metastability of the boronic species in CC conditions,^22^ the crude reaction product was directly treated with the iodobiphenyl **B**^8^ in similar *Suzuki*–*Miyaura* cross-coupling conditions to give the desired quarterphenyl building block **11** as orange oil in 31% isolated yield after CC. For the first step, a conversion of 69% of the amine to the boronic ester was determined by GC-MS. To profit from the remaining bromine substituent of **11** to further develop the oligophenyl scaffold more sever *Suzuki* reaction conditions were applied. Using the optimized catalyst SPhos Pd G2 in combination with higher temperature and elongated reaction times, the arylbromide **11** and the arylboronic pinacol ester **C**^8^ were coupled. Refluxing the precursors and reagents in a 4 : 1 toluene/water mixture for four days provided the hexaphenyl intermediate **12** in 69% yield after CC.

Compound **12** not only comprises all the carbon atoms of the target structure, it also exposes four methyl ester groups representing masked benzylic bromides. Reducing the fourfold methyl carboxylate **12** with DIBAL-H in DCM provided the fourfold benzylic alcohol **13** in 93% yield. The final conversion to the hexaphenyl intermediate exposing four benzylic bromides **14** was achieved by functional group transformation. Compound **13** was treated with phosphorus tribromide (PBr_3_) in DCM at room temperature to give **14** as white solid in 74% isolated yield after CC.

**Scheme 2:**
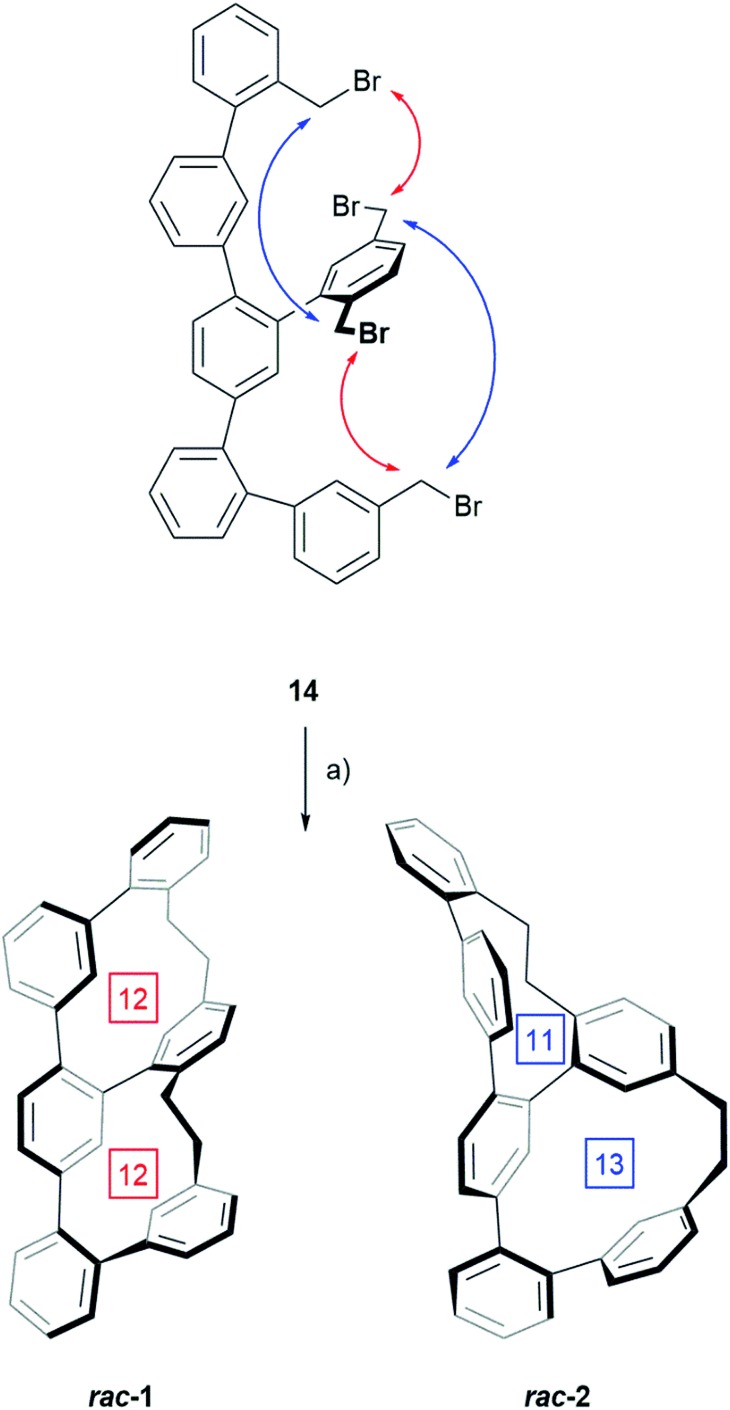
Synthesis of the two structural isomers ***rac*-1** and ***rac*-2** by twofold macro-cyclization: (a) PhLi, THF, 0 °C to RT, 2–3 h, 26% for the mixture ***rac*-1** and ***rac*-2**. The red and blue arrows point at the benzylic bromides engaged in the coupling reaction resulting in ***rac*-1** and ***rac*-2** respectively. The numbers in the square boxes denominate the carbon atoms in each macrocycle.

For the double macro-cyclization of **14**, only half of the benzyl bromides should be converted into an organolithium reagent and react intra-molecularly with a neighbouring benzyl bromide. The hope was that the intramolecular macrocyclization reaction is quick, and thus favored compared with intermolecular reactions or competing defunctionalization pathways *e.g.* due to remaining humidity. To our delight the intramolecular *Wurtz*-type coupling was successful by adding the precursor **14** dropwise to a 0 °C cooled solution of phenyl lithium. After allowing warming up to room temperature, the reaction was worked up and a mixture of both structural isomers **1** and **2** was isolated as white solid in 26% yield by CC (Scheme 2).

**Fig. 2:**
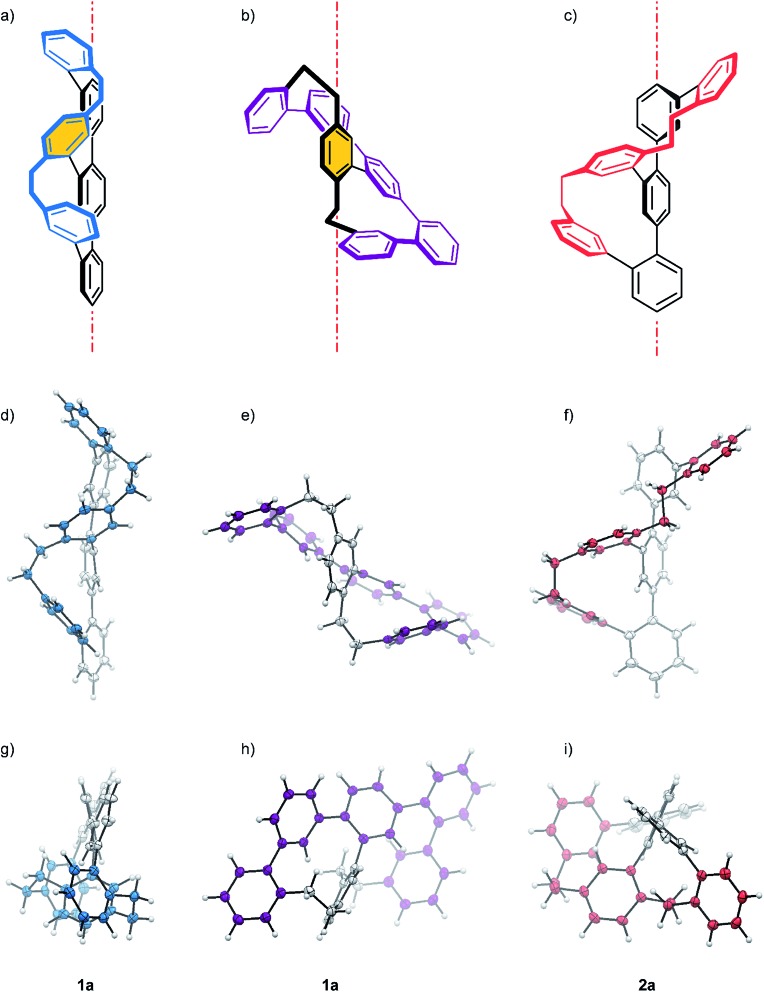
(a–c) Schematic representations of the *P* enantiomers **1a** (blue and purple) and **2a** (red). The red dot and dash lines represent the main axis. The X-ray structure of enantiomer **1a** (blue and purple) and **2a** (red) are displayed from different viewpoints. With (d)–(f) side- and (g)–(i) top-views of the structures with respect to their main axis. While (d) and (g) display the zig–zag arrangement of the longer oligomer, (e) and (h) display the helical arrangement of the purple quinquephenyl with respect to the xylene axis in **1a**. Color code: bridge = blue, purple or red, backbone = gray, hydrogen atoms = white.

The transformation of **14** to one of the two constitutional isomers requires two intramolecular ring closing reactions from which the first one decides, which isomer is obtained. Assuming an orthogonal arrangement between the central phenyl ring of the terphenyl backbone and the attached phenyl subunit with two benzylic bromides, the two *Wurtz*-type couplings can either occur between the pairs of neighbouring benzylic bromides indicated by red arrows or between the pairs suggested by blue arrows. While the reaction sequence suggested by the red arrows results in ***rac*-1** consisting of two equally sized macrocycles with 12 carbon atoms each, the alternative sequence displayed by blue arrows yields in the constitutional isomer ***rac*-2** consisting of a smaller and a larger cycle of 11 and 13 carbon atoms respectively. HPLC using a reversed stationary phase (Reprosil C18, MeCN, 100 mL min^–1^, 25 °C) allowed the separation of both structural isomers and according to the integration of the HPLC traces (see ESI Fig. S1†), 77% of the crude are the isomer with two equally sized rings ***rac*-1** and 23% are ***rac*-2**. Due to the small size of the sample (13.3 mg of crude ***rac*-1** and ***rac*-2**) preparative HPLC was considered for their separation. Using similar conditions on a preparative column, 6.2 mg of ***rac*-1** (12.1% yield) and 3.0 mg of ***rac*-2** (5.9% yield) were isolated. These samples of pure structural isomers ***rac*-1** and ***rac*-2** were enough material enabling the complete characterization by 1D and 2D NMR spectroscopy, UV-Vis spectroscopy, HRMS and single-crystal XRD (Fig. 2). Crystals suitable for X-ray analysis were obtained for both constitutional isomers ***rac*-1** and ***rac*-2** by slow vapor diffusion of DCM into solutions of the samples in *n*-hexane : *i*-PrOH (99 : 1). Both crystalized in the monoclinic space group *P*2_1_/*c* with a racemic unit cell comprising two molecules of both enantiomers (see ESI Fig. S3†). Fig. 2 displays the solid state structures of both structural isomers.

As expected, the oligo-phenylene-ethanylene structure of ***rac*-2** is wrapped helically around the terphenyl backbone (axial chirality). Torsion angles of 51.88° and 72.49° are observed between the phenyl-rings of the terphenyl backbone, in spite of the dimensional differences of both macrocycles of the structure (11 *vs.* 13 atoms). This rather small difference in torsion angles of both joints is only possible because of the variation in the arrangement of the ethyl bridges of the banister. While the two phenyl substituents of the ethyl bridge of the 13 atoms large macrocycle are fixing the ethyl link in a *syn* arrangement (lower ethyl bridge in red in Fig. 2f), the ethyl link of the smaller 11 atoms sized macrocycle is fixed in an *anti* configuration (upper ethyl bridge in red in Fig. 2f). A more precise inspection of both ethyl-bridges display an almost perfect “*gauche*” conformation with a torsion angle of 70.81° for the *syn* ethyl bridge, and a torsion angle of 161.19° for the phenyl substituents of the *anti* ethyl bridge in the smaller macrocycle. The entire oligo-phenylene-ethanylene banister covers an angle of 126.37° around the terphenyl backbone.

Also for ***rac*-1** with two equally sized macrocycles (12 atoms) a helical arrangement with the phenylene-ethanylene oligomer wrapped around the terphenyl backbone was expected. To our surprise, the solid state structure of ***rac*-1** displayed a zig–zag arrangement of the phenylene-ethynylene oligomer (blue in Fig. 2a, d, and g), with both ethyl bridges in a *syn* configuration and very limited effect on the torsion angles of the terphenyl backbone (black in Fig. 2a). Both terminal phenyl rings of the terphenyl subunit are almost coplanar with the central phenyl ring only slightly bent out of the plane, as reflected by the torsions angles of 32.50° and 37.56° in the terphenyl backbone. Both ethyl bridges of the banister are fixed as almost perfect “*gauche*” conformation with torsion angles between both phenyl substituents of 65.58° and 67.14° respectively. After studying the structure for a moment, we realized that the solid state structure can be described as a helically wrapped oligomer, but the wrapping oligomer and the axis have to be redefined. Namely the quinquephenyl (purple in Fig. 2b, e, and h) wraps helically around the *para*-xylene axis (highlighted by an orange background in Fig. 2a and b). This redefinition considerably shortens the axis of the structure with 5.805 Å between the α and α′ carbon of the *para*-xylene compared to the 11.396 Å of the 4,4′′-positions of the terphenyl subunit. The quinquephenyl covers an angle of 177.60° as helical banister around the *para*-xylene axis. The surprising arrangement of the Geländer model compound ***rac*-1** analyzed by X-ray analysis evoke the question whether or not this is a consequence of intermolecular packing forces in the solid state. To analyze the conformation of the dissolved structure extensive 1D and 2D NMR studies were performed. The recorded NOE signals suggest a very comparable arrangement of both macrocycles of ***rac*-1** as favored conformation dissolved in toluene-d_8_. A comprehensive discussion of the conformational analysis of dissolved ***rac*-1** is provided in the ESI (see ESI Fig. S2†).

**Fig. 3:**
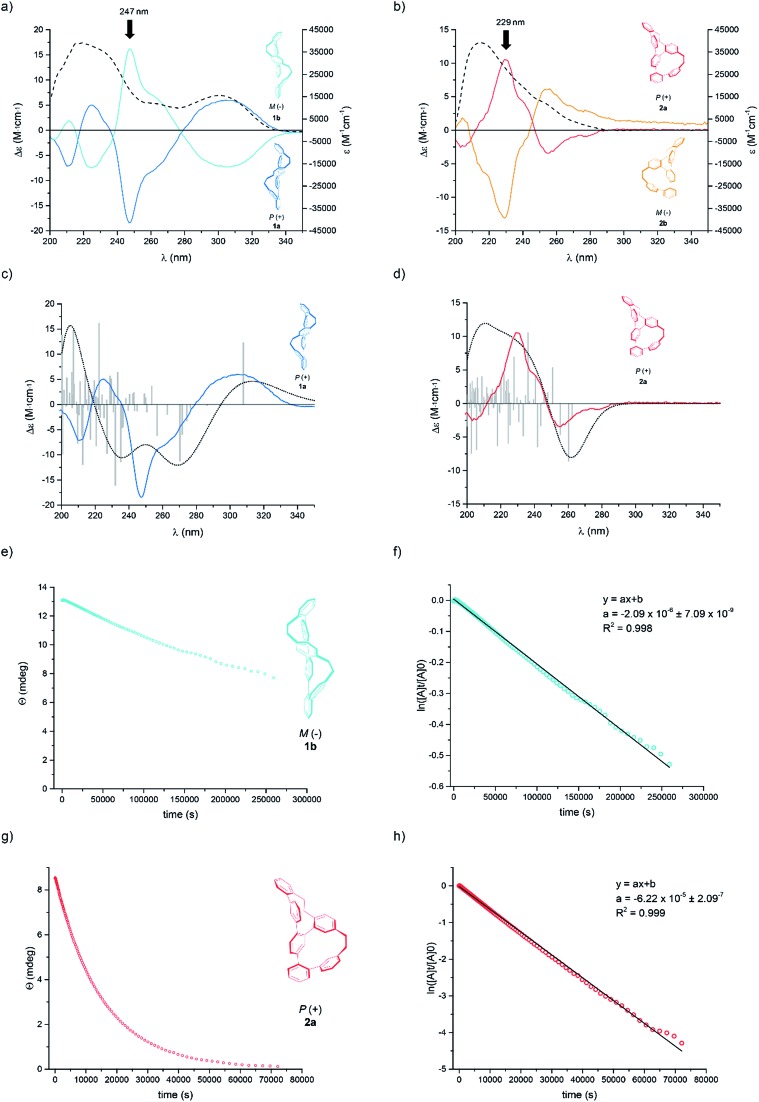
The UV/Vis (dashed lines) and CD plots (solid coloured line) for Geländer system (a) **1a**,**b** and (b) **2a**,**b** in 99 : 1 *n*-hexane : *i*-PrOH at 10 °C. The experimental and calculated CD spectra of (c) **1a** and (d) **2a** by using TD-B3LYP/6-31G** functional with 75 triplet and 75 singlet excitations; total: 150 states; width 0.4 eV. The calculated spectra are based on the data from the single-crystal structures. Colored: experimental spectra, black dots: calculated spectra, grey bars: calculated transitions. Decay of the CD signals for (e) **1b** and (g) **2a** together with their linear fit obtained by plotting the ln of the signal against time for (f) **1b** and (h) **2a**. The slope of the linear fit provides the rate constant *k*_rac_, which gives access to the Gibbs free energy of the racemization process Δ*G*≠rac at 25 °C (see ESI†).

The racemates of both ***rac*-1** and ***rac*-2** isomers could be successfully baseline separated into their enantiomers by HPLC using a chiral stationary phase (Chiralpak IA, 1 mL min^–1^, 99 : 1 *n*-hexane : *i*-PrOH, 18 °C) (see ESI Fig. S1†). For each oligomer the corresponding *M* and *P* helices were isolated in high enantiomeric purities (>99% ee). Further evidence, that the isolated fractions were enantiomers, was confirmed by circular dichroism measurements (Fig. 3a and b). We recorded CD and UV-Vis spectra for each isomer, **1a**, **1b**, **2a** and **2b**, in *n*-hexane : *i*-PrOH (99 : 1) at 10 °C immediately after HPLC separation. Complementary Cotton effects were observed in each case (**1a**,**b**: *λ* = 306 nm, 247 nm, 225 nm and 210 nm; **2a**,**b**: *λ* = 255 nm, 229 nm and 204 nm).

Both samples **1** and **2** crystallized as racemate and thus, solid-state analysis did not allow us to assign the configuration. However, the absolute configuration was determined by comparison of the calculated and measured CD spectra (Fig. 3a–d). For the calculation, the single-crystal structures were used as starting points with B3LYP/6-31G** basis set. Time-dependent calculations (TD-B3LYP/6-31G**, 150 states) with 75 triplet and 75 singlet excitations were performed and the obtained signals of the Cotton bands were compared with the experimental spectra. A good agreement was observed between experimentally recorded and computer calculated CD spectra for both systems, which allowed the final assignment of **1a** and **2a** as the *P* helices and **1b** and **2b** as the *M* helices.

Of particular interest was the racemization of the Geländer oligomers **1** and **2**. Most likely the racemization mechanism of both Geländer systems obey *Mislow's* “*Euclidean* rubber glove” enantiomerization pathway.^23^–^25^ This means, that the bicyclic helical structures are interconverted into their mirror images by deformation and rotations around single bonds without ever adopting an achiral conformation. In the case of both Geländer systems **1** and **2**, a planar achiral conformation is in principle possible (planar representation in Fig. 1), but very unlikely because of its strain. Due to their subtle structural differences the two macrocycles of each Geländer structure are not expected to adapt planar transition states simultaneously, but rather a pathway with subsequent transitions of both macrocyclic subunits is more likely. Thus, axial chirality should be maintained during the entire enantiomerization process supporting the hypothesis of a “*Euclidean* rubber glove” like pathway. The here presented results however do not proof this hypothesis, but rather suggest it as the most likely option. Applying this picture, both macrocycles are flexible enough to unfold and refold into their mirror image without breaking a bond like a right-handed rubber glove, which can be turned inside out to become a left-handed rubber glove. We were particular interested in the effects of the various ring sizes present in **1** compared with **2** on the racemization dynamics. The dynamics of both structures was analyzed by CD spectroscopy as well as by analyzing samples exposed to different temperatures over various periods by chiral HPLC.^26^,^27^ In particular the rate constants *k*_rac_, the half-lives *t*_(1/2)_, and the racemization barriers Δ*G*≠rac(*T*) were determined by time dependent CD spectroscopy at 25 °C. In a second series an enantiopure sample was exposed to different temperatures and the concentrations of both enantiomers were analyzed after different periods by chiral HPLC giving access to thermodynamic parameters like the enthalpies Δ*H*≠rac and entropies Δ*S*≠rac of the racemization process.^28^

A racemic sample was separated into its enantiomers by chiral HPLC and an enantiopure sample was immediately subjected to CD spectroscopy at 25 °C. In periodic intervals the CD signal of the sample was recorded at the wavelength of the most intense Cotton band (**1b**: *λ*_max_ = 229 nm, **2a**: *λ*_max_ = 247 nm). Fig. 3(e) and (g) display the exponential decay of the CD signal of **1b** and **2a** respectively. The racemization is a first order process and thus, *k*_rac_ is the slope of the straight line obtained by plotting the natural logarithm of the CD signal against time (Fig. 3(f) and (h)). The data collected by these studies are summarized in Table 1.

**Table 1:** Measured rate constants, half-lives, *Gibbs* free energies, enthalpies and entropies of compound **1** and **2** at 25 °C

Compound	Ratio of macrocycles	Analytical method	*k*_rac_ [s^–1^]	*t*_1/2_ [h]	Δ*G*≠rac [kJ mol^–1^]	Δ*H*≠rac [kJ mol^–1^]	Δ*S*≠rac [J mol^–1^ K^–1^]
**1**	[12 : 12]	Dynamic CD	2.1 × 10^–6^	91.98	103.7	—	—
		Chiral HPLC	2.2 × 10^–6^	85.88	103.6	101.7	–6.6
**2**	[11 : 13]	Dynamic CD	6.2 × 10^–5^	3.09	95.2	—	—
		Chiral HPLC	4.9 × 10^–5^	3.89	95.9	66.2	–99.8

The racemization kinetics of both helical model compounds **1** and **2** differs considerably. An increase of more than 8 kJ mol^–1^ was observed in the *Gibbs* free energy of the racemization (Δ*G*≠rac(*T*)) of **1** consisting of two equally sized macrocycles compared with its constitutional isomer **2**. Initially the arrangements of the ethanyl-linkers in the solid-state structures (see Fig. 2) were considered as stability indicators and thus, the helical structure of **2** with one *anti*-arranged ethanyl linker was expected to be thermodynamically favored compared to **1** with two *syn*-arranged ethanyl linkers. However, the two even-sized macrocycles of **1** allow a very compact arrangement with the zig–zag arranged oligo-phenylene-ethanylene thread leaving the terphenyl-backbone almost flat. The measured data clearly document that unwrapping this compact structure and refolding in its mirror image is energetically more costly than the analogue process with the helical structure **2**. While the energy difference of the racemization barrier seems with 8.4 kJ mol^–1^ not too excessive, it translates in an about 30 times larger half-life time of the pure enantiomer of the more stable constitutional isomer **1** compared with **2**.

The thermodynamic parameters involved in the process of unwrapping and refolding the helical structures of **1** and **2** were estimated by a *van't Hoff* plot based on racemization kinetics determined at different temperatures by chiral HPLC (see ESI†). To benchmark the comparability of both methods, the speed of racemization (*k*_rac_) was determined at the temperature of the CD experiment as well (25 °C). As displayed in Table 1, very comparable values were obtained with both methods. The thermodynamic parameters further corroborate the hypothesis of a compact helical arrangement with an oligo-phenylene-ethanylene thread tightly fixed in a zig–zag arrangement for the Geländer model compound **1**. Its racemization process is almost exclusively enthalpically driven, pointing at very limited degrees of freedom for the entire bis-macrocyclic system. In contrast to that, the racemization of **2** has considerable entropic contributions, as expected for a process profiting from the wiggling of the less tightly wrapped oligo-phenylene-ethanylene thread.

Comparing the racemization of the two purely carbon based Geländer model compounds **1** and **2** with the ones comprising chalcogen ether bridges (**3–7** in Fig. 1), shows an increase in the stability for both structures compared with their chalcogen comprising analogues. The racemization process of **2** is with 95.9 kJ mol^–1^ about 3.5 kJ mol^–1^ larger than the ones obtained for the model compounds **6** and **7** (92.4 kJ mol^–1^ and 92.3 kJ mol^–1^ respectively, see ESI Table S19†), which also had two differently sized macrocycles. A more pronounced increase in the stability was observed for the helical structure **1** consisting of two equally sized macrocycles. The comparison of the equally sized analogues **1** with **3–5** display a substantial stability increase from *e.g.* 95.8 kJ mol^–1^ for **3** to 103.7 kJ mol^–1^ for **1**. While this follows the expectation for the reduction of ring sizes from [13 : 13] in the case of **3–5** to [12 : 12] in the case of **1** which was the initial motivation of this study, the comparison remains difficult due to the unexpected variation in the helical packing of **1**. Whether the increase in stability emerges from the alternative wrapping of the two differently sized threads or really reflects the variation in the dimensions of the macrocycles has to be investigated with the next generation of model compounds. However, the model compound **1** is the most stable Geländer system synthesized so far.

## Conclusion

The two bis-macrocyclic structures **1** and **2** were synthesized and fully characterized. These are the first all-carbon Geländer model compounds and their solid-state structures were analyzed by X-ray analyses. While the design concept of compensating the length mismatch of two interlinked oligomers by wrapping the longer one helically around the shorter one was successful in **2**, an alternative zig-zag arrangement of the longer oligomer with the ethanyl-bridges in a *syn* arrangement was favored in the case of **1**. The structure of **1** is a helical arrangement of the quinquephenyl belt around the *para*-xylene core. Investigation of the racemization dynamics displayed the superior kinetic thermodynamic stability of the compact arrangement of **1**, also compared with earlier reported Geländer model compounds comprising chalcogen ether linkers. However, the variation in the intramolecular packing of the two oligomer strands makes it difficult to track the origin of the increased stability.

We are currently working on all-carbon model compounds with alternative ring sizes to identify the parameter determining the helical packing and the resulting stability in further details. In addition, we are working on Geländer model compounds with electron delocalization in the helical structure.

## Conflicts of interest

There are no conflicts to declare.

## Supplementary Material

Supplementary information

Crystal structure data
