# Supplementary material for: Molecular dynamic staircases: all-carbon axial chiral “Geländer” structures
Source: Chem Sci. 2018 Jun 5;9(26):5758–66. doi: 10.1039/c8sc01707g (PMC6050599; doi:10.1039/c8sc01707g)
Supplement: Supplementary file 1 [file SC-009-C8SC01707G-s001.pdf]

Electronic Supplementary Material (ESI) for Chemical Science.
This journal is © The Royal Society of Chemistry 2018

Electronic Supplementary Information

Molecular Dynamic Staircases: All-Carbon Axial Chiral "Geländer" Structures

Rajesh Mannancherry,[a] Michel Rickhaus,[a] Daniel Häussinger,[a] Alessandro Prescimone,[a] Marcel Mayor[a,b,c]

---

[a] Department of Chemistry
University of Basel
St. Johanns-Ring 19
4056 Basel, Switzerland
E-mail: marcel.mayor@unibas.ch

[b] Institute for Nanotechnology (INT)
Karlsruhe Institute of Technology (KIT)
P. O. Box 3640
76021 Karlsruhe, Germany

[c] Lehn Institute of Functional Materials (LIFM)
School of Chemistry
Sun Yat-Sen University (SYSU)
Guangzhou 510275, China

Electronic Supplementary Information

Table of Contents

Table of Contents.. 2

Synthetic procedures 4

4-Bromo-3-(4,4,5,5-tetramethyl-1,3,2-dioxaborolan-2-yl)aniline (**9**):....................................... 5

Dimethyl 5'-amino-2'-bromo-[1,1'-biphenyl]-2,5-dicarboxylate (**10**):.. 6

Trimethyl 4''-bromo-[1,1':2',1'':3'',1'''-quaterphenyl]-2''',3,5'''-tricarboxylate (**11**): 7

Dimethyl 3''-(2,5-bis(methoxycarbonyl)phenyl)-[1,1':2',1'':4'',1''':3''',1''''-quinquephenyl]-2'''',3-dicarboxylate (**12**): 8

3''-(2,5-bis(hydroxymethyl)phenyl)-2'''',3-bis(hydroxymethyl)-1,1':2',1'':4'',1''':3''',1''''-quinquephenyl (**13**).. 9

3''-(2,5-bis(bromomethyl)phenyl)-2'''',3-bis(bromomethyl)-1,1':2',1'':4'',1''':3''',1''''-quinquephenyl (**14**)...10

All-carbon Geländer system **1** and **2**..12

HPLC Chromatograms of Geländer System **1** and **2** 14

NMR studies of Geländer System **1** and **2**...15

1 in toluene-d_8 COSY...17

1 in toluene-d_8 NOESY 18

1 in toluene-d_8 HMBC 19

2 in toluene-d_8 COSY...20

2 in toluene-d_8 TOCSY...21

Single Crystal X-Ray Analysis of **1** 23

Single Crystal X-Ray Analysis of **2** 24

Determination of torsion angles of macrocycles **1** and **2**...25

Crystal packing of **1** and **2** 25

^{1}H-, ^{13}C-NMR ($CDCl_3$, 400/101 MHz, 25 °C) and HR-ESI spectra of compound **9** 26

^{1}H-, ^{13}C-NMR ($CDCl_3$, 400/101 MHz, 25 °C) and HR-ESI spectra of compound **10** 28

HR-ESI spectra of compound **15** 30

^{1}H-, ^{13}C-NMR (CD_3CN, 400/101 MHz, 25 °C) and HR-ESI spectra of compound **11**.............31

^{1}H-, ^{13}C-NMR (CD_3CN, 400/101 MHz, 25 °C) and HR-ESI spectra of compound **12**.............33

^{1}H-, ^{13}C-NMR (CD_3CN, 400/101 MHz, 25 °C) and HR-ESI spectra of compound **13**.............35

^{1}H- and ^{13}C-NMR (Toluene-d_8, 600/125 MHz, 25 °C) and HR-ESI spectra of compound **1**....37
1 in toluene-d_8 ^{1}H-NMR....37
1 in toluene-d_8 ^{13}C-NMR....38
^{1}H-NMR (Toluene-d_8, 600/125 MHz, 25 °C) and HR-ESI spectra of compound **2**....40
2 in toluene-d_8 ^{1}H-NMR....40
UV-Vis spectroscopy....42
Experimental UV-Vis and CD: Data of **1**....43
Experimental UV-Vis and CD: Data of **2**....50
Calculated CD: Data....57
Determination of $G^{\neq}_{\text{rac (298 K)}}$ by circular dichroism....82
Determination of $\Delta G^{\neq}_{\text{rac (T)}}$, $\Delta H^{\neq}_{\text{rac}}$ and $\Delta S^{\neq}_{\text{rac}}$by dynamic HPLC....95

Synthetic procedures

General Procedures: All chemicals were directly used for the synthesis without further purification, unless stated differently. Solvents for photophysical measurements were HPLC grade. Dry solvents were used as crown cap and purchased from *Acros Organics* and *Sigma-Aldrich*. NMR solvents were obtained from *Cambridge Isotope Laboratories, Inc.* (Andover, MA, USA). ^{1}H-NMR and ^{13}C-NMR were recorded on a *Bruker DPX-NMR* (400 MHz) instrument or on a Bruker Ascend 600 MHz Avance III HD. Chemical shifts (δ) are reported in parts per million (ppm) relative to the residual solvent peak. DART-MS was measured on a IonSense DART-SVP100 (He, 450 °C) connected to a Shimadzu LC-2020. Gas Chromatography (GC-MS) was performed on a *Shimadzu* GCMS-QP2010 SE gas chromatograph system, with a ZB-5HT inferno column (30 m x 0.25 mm x 0.25 mm), at 1 mL/min He-flow rate (split = 20:1) with a *Shimadzu* mass detector (EI 70 eV) was used. For high resolution mass spectra (HRMS) a HR-ESI-ToF-MS measurement on a *maXis^{TM} 4G* instrument from *Bruker* was performed. High-resolution electron ionization mass spectrometry was performed on a *Thermo DFS* (*ThermoFisher Scientific*, Bremen, Germany) double-focusing magnetic sector mass spectrometer (geometry BE). Mass spectra were measured in electron impact (EI) mode at 45 eV, with solid probe inlet, a source temperature of 200°C, an acceleration voltage of 5 kV, and a resolution of 10’000. The instrument was scanned between e.g. *m/z* 300 und 350 at scan rate of 100-200 s / decade in the electric scan mode. Perfluorokerosene (PFK, Fluorochem, Derbyshire, UK) served for calibration. Column chromatography was performed with SiliaFlash® P60 from *SILICYCLE* with a particle size of 40-63 µm (230-400 mesh) and for TLC *Silica gel 60* F_{254} glass plates with a thickness of 0.25 mm from *Merck* were used. The detection was observed with a UV-lamp at 254 or 366 nm. For HPLC a Shimadzu LC-20AB, LC-20AD, LC-20AP and a LC-20AT HPLC, respectively, was used equipped with a diodearray UV/Vis detector (SPD-M20A VP from Shimadzu, λ = 200-600 nm) and a column oven Shimadzu CTO-20AC. The used column for reverse phase was a Reprosil 100 C18, 5 µm, 250 x 16 mm; Dr. Maisch GmbH and for chiral separation a Chiralpak IA, 5 µm, 4.6 x 250 mm; Daicel Chemical Industries Ltd. CD measurements were performed with a Chirascan CD Spectrometer in *n*-hexane:*i*-PrOH 99:1 mixture at 10 °C in 1 cm quartz glass cuvettes directly after the chiral HPLC. All solutions were prepared and measured under air saturated conditions. Calculations were performed using Gaussian G09 version C.01.

4-Bromo-3-(4,4,5,5-tetramethyl-1,3,2-dioxaborolan-2-yl)aniline (9):

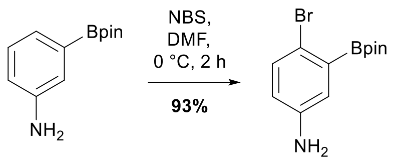

8 **9**

An oven-dried 25 mL Schlenk tube was purged with argon and charged with 3-(4,4,5,5-tetramethyl-1,3,2-dioxaborolan-2-yl)aniline (**8**, 4.00 g, 17.9 mmol, 1.0 eq.), NBS (3.5 g, 19.7 mmol, 1.1 eq.) and DMF (60 mL). The reaction mixture was cooled to -15 °C with acetone and dry ice and the solution was left stirring for 2 h (Reaction control by GC-MS). After 1 h the reaction was treated with 10% aq. $Na_2S_2O_3$ (80 mL) and was washed with diethylether (3 x 80 mL). The combined organic layers were washed with water (2 x 200 mL), brine (200 mL) and then dried over Na_2SO_4. After removal of the solvent under reduced pressure, the product was isolated by flash column chromatography (SiO_2, cyclohexane/ethyl acetate 5:1 to 1:1) to obtain the product **9** (2.50 g, 8.95 mmol, 93%) as light brown oil.

Analytical data for 9:

^{1}H NMR (400 MHz, $CDCl_3$, 25 °C) δ = 7.30 (d, *J* = 8.5 Hz, 1H), 6.98 (d, *J* = 3.0 Hz, 1H), 6.63 (dd, *J* = 8.5, 3.0 Hz, 1H), 1.37 (s, 12H) ppm.

^{13}C NMR (101 MHz, $CDCl_3$, 25 °C) δ = 144.7, 133.4, 123.0, 119.0, 116.2, 100.1, 84.4, 77.5, 77.2, 76.8, 24.9 ppm.

GC-MS (EI +, 70 eV): m/z (%) = 300.1 (12), 299.1 (92), 298.1 (35), 297.1 (97) $[M]^+$, 296.1 (25), 284.1 (13), 282.0 (13), 218.1 (17), 200.0 (30), 199.0 (76), 198.0 (48), 197.0 (81), 196.0 (19), 177.1 (11), 176.1 (100), 175.1 (35), 160.1 (19), 159.1 (20), 134.1 (13), 133.1 (10), 132.1 (49), 119.1 (25), 118.1 (41), 117.1 (23), 92.1 (19), 91.1 (45), 90.1 (13), 85.1 (34), 67.1 (10), 65.1 (24), 59.1 (15), 57.1 (20), 55.1 (14).

Analytical data can be found in reference[1].

---

1 T. Kamei, A. Ishibashi and T. Shimada, *Tetrahedron Lett.*, 2014, **55**, 4245–4247.

Dimethyl 5'-amino-2'-bromo-[1,1'-biphenyl]-2,5-dicarboxylate (10):

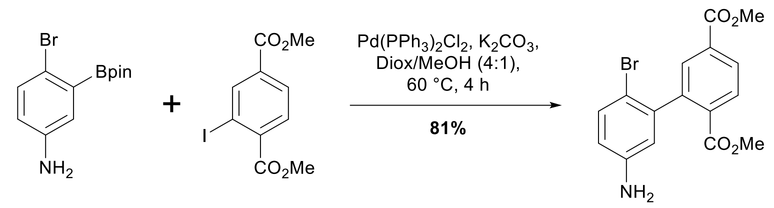

9 **A** **10**

To an argon flushed and vacuum dried Schlenk tube was consecutively added dimethyl-iodoterephthalate (**A**, 2.72 g, 8.49 mmol, 1.00 eq.), starting material **9** (2.53 g, 8.49 mmol, 1.00 eq.) and potassium carbonate (3.52 g, 25.5 mmol, 3.00 eq.). 1,4-dioxane (24 mL) and MeOH (6 mL) were added and the solution was degassed for 15 min before adding $Pd(PPh_3)_2Cl_2$ (119 mg, 0.17 mmol, 2 mol%) and heating to 60 °C for 4 h. DCM (30 mL) was added and the suspension was filtered off. The solvent was removed under reduced pressure and after flash column chromatography (SiO_2, cyclohexane/ethyl acetate 1:1) the product **10** (2.51 g, 8.49 mmol, 81%) was yielded as an orange/yellow oil.

Analytical data for 10:

^{1}H NMR (400 MHz, $CDCl_3$, 25 °C) δ = 8.10 (dd, *J* = 8.1, 1.7 Hz, 1H), 8.03 (dd, *J* = 8.1, 0.6 Hz, 1H), 7.93 (dd, *J* = 1.7, 0.5 Hz, 1H), 7.35 (dd, *J* = 8.2, 0.7 Hz, 1H), 6.66 – 6.54 (m, 2H), 3.94 (s, 3H), 3.72 (s, 3H) ppm.

^{13}C NMR (101 MHz, $CDCl_3$, 25 °C) δ = 166.9, 166.2, 145.4, 142.5, 142.3, 134.3, 133.0, 132.8, 132.2, 130.2, 128.9, 117.0, 116.2, 111.1, 52.6, 52.5.

GC-MS (EI +, 70 eV): m/z (%) = 286.1 (5), 285.1 (30), 284.1 (100) $[M-Br]^+$, 270.0 (19), 269.0 (64), 210.0 (8), 209.0 (6), 182.0 (8), 154.1 (6), 142.1 (9), 139.1 (11), 126.6 (6), 119.1 (12), 111.1 (6), 82.8 (8).

HRMS (ESI-ToF): calc. for $C_{16}H_{15}BrNO_4$ 364.0179 $[M+H]^+$; found 364.0181; calc. for $C_{16}H_{14}BrNNaO_4$ 385.9998 $[M+Na]^+$; found 385.9996.

Trimethyl 4''-bromo-[1,1':2',1'':3'',1'''-quaterphenyl]-2''',3,5'''-tricarboxylate (11):

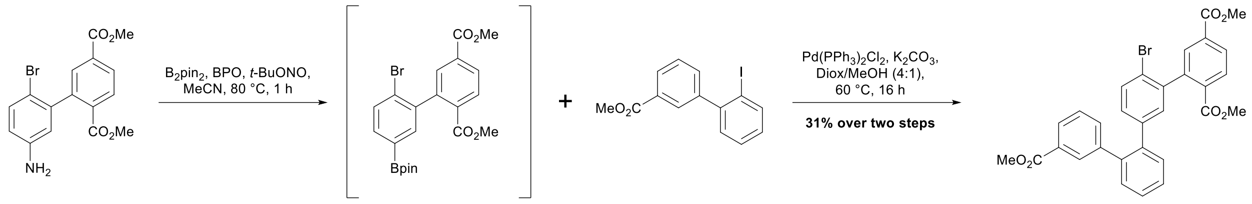

10 **15** **B** **11**

A 250 mL two-neck round bottom flask equipped with reflux condenser was charged with B_2pin_2 (8.99 g, 35.4 mmol, 1.00 eq.) and BPO (229 mg, 0.708 mmol, 2 mol%). Starting material **10** (12.9 g, 35.4 mmol, 1.00 eq.) was dissolved in dry MeCN (60 mL) and *t*-BuONO (6.37 mL, 53.1 mmol, 1.50 eq.) was then added in succession. The mixture was allowed to stir for 1 h at 80 °C. The solution was concentrated and the crude residue was redissolved in EtOAc (20 mL). The solution was washed with 1M HCl (20 mL), H_2O (2 x 20 mL) and then dried over Na_2SO_4. After filtration the solution was removed and the crude product was used directly for the next reaction.

To an argon flushed and oven-dried three-neck round bottom flask (250 mL) equipped with reflux condenser was consecutively added the crude mixture (8.00 g), methyl 2'-iodo-[1,1'-biphenyl]-3-carboxylate (**B**, 5.68 g, 16.8 mmol, 0.47 eq.) and K_2CO_3 (6.97 g, 50.4 mmol, 1.42 eq.). 1,4-dioxane (96 mL) and MeOH (24 mL) were added and the solution was degassed for 15 min. $Pd(PPh_3)_2Cl_2$ (354mg, 0.504 mmol, 3 mol%) was added and the reaction mixture was heated to 60 °C for 16 h. DCM (50 mL) was added and the suspension was filtered off. The solvent was removed under reduced pressure and flash column chromatography (SiO_2, cyclohexane/ethyl acetate 20:1) yielded the product **11** (6.07 g, 10.9 mmol, 31% over two steps) as a yellow/orange oil.

Analytical data for 15:

GC-MS (EI +, 70 eV): m/z (%) = 395.0 (100) $[M-Br]^+$, 394.0 (25), 396.0 (23), 295.0 (12), 59.0 (7), 313.0 (5), 294.0 (5), 248.9 (5), 397.0 (4), 206.9 (4)

HRMS (ESI-ToF): calc. for $[C_{22}H_{25}BBrO_6]$ 475.0922 $[M+H]^+$, found 475.0921; calc. for $[C_{22}H_{24}NaO_6]$ 497.0742 $[M+Na]^+$, found 497.0750.

Analytical data for 11:

^{1}H NMR (400 MHz, CD_3CN, 25 °C): δ = 8.03 – 7.93 (m, 2H), 7.88 (dt, *J* = 7.3, 1.7 Hz, 1H), 7.79 (td, *J* = 1.8, 0.7 Hz, 1H), 7.51 – 7.28 (m, 8H), 7.07 (dd, *J* = 8.3, 2.3 Hz, 1H), 6.90 (d, *J* = 2.3 Hz, 1H), 3.90 (s, 3H), 3.80 (s, 3H), 3.62 (s, 3H).

^{13}C NMR (101 MHz, CD_3CN, 25 °C) δ = 167.4, 166.9, 166.4, 142.7, 142.4, 142.1, 141.4, 140.3, 139.8, 135.4, 134.9, 134.0, 132.8, 132.7, 132.4, 131.5, 131.4, 131.4, 131.2, 131.1, 131.0, 129.8, 129.2, 129.2, 128.7, 121.9, 53.1, 52.9, 52.7.

GC-MS (EI +, 70 eV): m/z (%) = 481.0 (7), 480.0 (31), 479.0 (100) $[M-Br]^+$, 302.0 (6), 300.0 (7), 224.0 (18), 207.0 (5), 151.1 (6), 150.1 (11), 144.7 (6).

HRMS (ESI-MS, +): calc. for $[C_{30}H_{24}BrO_6]$ 559.07508 $[M+H]^+$, found 559.07543; calc. for $[C_{30}H_{23}BrNaO_6]$ 581.05702 $[M+Na]^+$, found 581.05630.

Dimethyl 3''-(2,5-bis(methoxycarbonyl)phenyl)-[1,1':2',1'':4'',1''':3''',1''''-quinque-phenyl]-2'''',3-dicarboxylate (12):

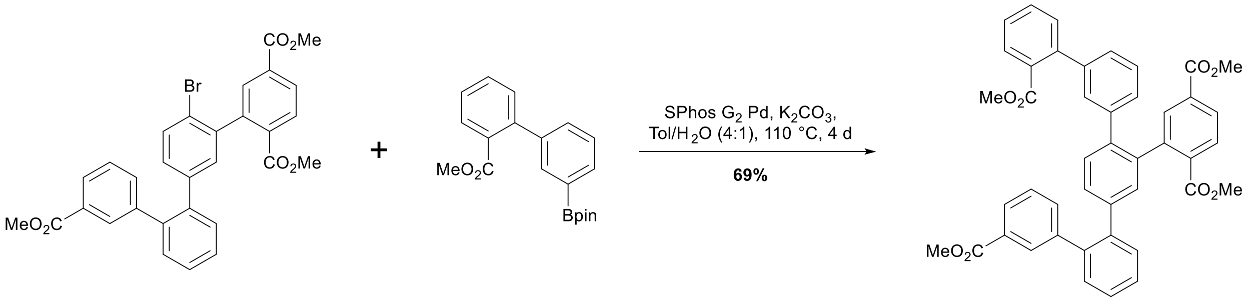

11 **C** **12**

To an argon flushed and vacuum dried Schlenk tube was consecutively added starting material **11** (1.76 g, 3.15 mmol, 1.00 eq.), methyl 3'-(4,4,5,5-tetramethyl-1,3,2-dioxaborolan-2-yl)-[1,1'-bi-phenyl]-2-carboxylate (**C**, 1.17 g, 3.47 mmol, 1.10 eq.) and K_2CO_3 (5.40 g, 39.1 mmol, 12.4 eq.). Dry toluene (40 mL) and H_2O (10 mL) were added and the solution was degassed for 15 min before adding SPhos Pd G_2 (51.7 mg, 0.126 mmol, 4 mol%). The reaction mixture was heated to 110 °C for 4 days. EtOAc (30 mL) was added and the suspension was filtered off. The solvent was removed under reduced pressure. The product **12** (1.49 g, 2.16 mmol, 69%) was yielded by flash column chromatography (SiO_2, cyclohexane/ethyl acetate 5:1) as a yellow/orange solid.

Analytic data for 12:

^{1}H NMR (400 MHz, CD_3CN, 25 °C) δ = 7.92 – 7.80 (m, 3H), 7.72 – 7.62 (m, 2H), 7.55 – 7.23 (m, 11H), 7.15 (t, J = 7.7 Hz, 1H), 7.08 – 6.95 (m, 3H), 6.93 – 6.86 (m, 2H), 3.86 (s, 3H), 3.77 (s, 3H), 3.56 (s, 3H), 3.45 (s, 3H).

^{13}C NMR (101 MHz, CD_3CN, 25 °C): δ = 169.60, 167.43, 167.40, 166.60, 143.27, 142.63, 142.31, 141.54, 141.11, 141.02, 140.57, 140.46, 140.38, 139.83, 139.62, 135.93, 135.48, 133.50, 133.32, 132.27, 132.09, 131.91, 131.55, 131.38, 131.05, 130.62, 130.52, 130.50, 130.38, 130.35, 130.29, 129.35, 129.17, 129.15, 128.99, 128.77, 128.60, 128.58, 128.30, 127.83, 53.04, 52.74, 52.63, 52.47.

DART-MS (450 °C, +): m/z (%) = 710.1 (13), 709.1 (44), 708.1 ($M+NH_4^+$, 100), 707.3 (7), 691.0 ($M+H^+$, 6), 659.2 (6).

HRMS (ESI-ToF): calc. for [$C_{44}H_{35}O_8$] 691.2326 $[M+H]^+$, found 691.2321; calc. for [$C_{44}H_{34}NaO_8$] 713.2146 $[M+Na]^+$, found 713.2145.

3''-(2,5-bis(hydroxymethyl)phenyl)-2'''',3-bis(hydroxymethyl)-1,1':2',1'':4'',1''':3''',1''''-quinquephenyl (13)

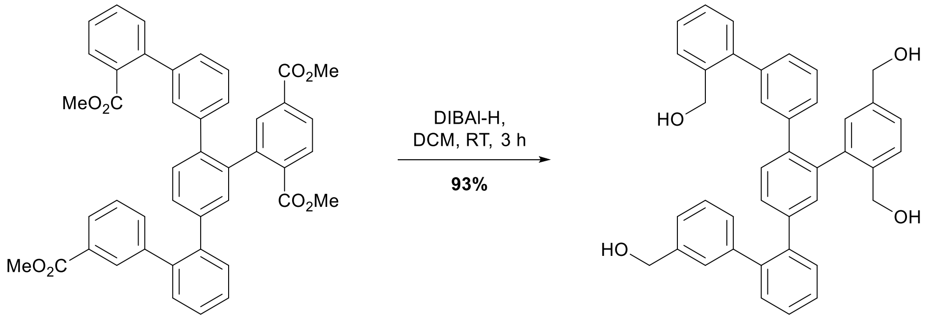

12 **13**

An oven dried, argon flushed 250 mL two-neck round bottom flask was equipped with dropping funnel and was charged with starting material **12** (1.18 g, 1.71 mmol, 1.00 eq.) and dry DCM (50 mL). DIBAl-H (1M in cyclohexane, 17.1 mL, 17.1 mmol, 10.0 eq.) was added dropwise to the reaction mixture at RT and the reaction mixture was stirred for 3 h. The mixture was cooled to 0 °C, diluted with DCM (20 mL) and then treated sequentially with H_2O (20 mL), 10% aq. NaOH (20

mL) and with H_2O (20 mL) again. The solution was warmed to RT and left stirring for 15 min. The organic phase was separated and the aqueous phase was back extracted with DCM (3 x 40 mL). The combined organic layers were dried over Na_2SO_4, filtered off and concentrated in *vacuo* to yield the product **13** (923 mg, 1.59 mmol, 93%).

Analytic data for 13:

^{1}H NMR (400 MHz, CD_3CN, 25 °C) δ = 7.52 – 7.36 (m, 7H), 7.32 – 7.17 (m, 8H), 7.14 – 7.06 (m, 3H), 6.99 (t, *J* = 1.8 Hz, 1H), 6.91 (dd, *J* = 7.6, *J* = 1.5 Hz, 1H), 6.84 – 6.81 (m, 1H), 6.72 (d, *J* = 1.8 Hz, 1H), 4.42 (ddd, *J* = 22.8, 5.7, 1.7 Hz, 4H), 4.14 – 4.00 (m, 3H), 3.84 (qd, *J* = 13.6, 5.4 Hz, 2H), 3.61 (t, *J* = 5.8 Hz, 1H), 3.46 (t, *J* = 5.8 Hz, 1H), 3.32 (t, *J* = 5.4 Hz, 1H), 3.09 (t, *J* = 5.5 Hz, 1H).

^{13}C NMR (101 MHz, CD_3CN, 25 °C) δ = 142.67, 142.46, 141.58, 141.56, 141.31, 141.29, 141.01, 141.00, 140.69, 140.19, 139.92, 139.56, 139.34, 138.92, 133.47, 131.46, 131.29, 131.26, 130.88, 130.64, 130.59, 130.13, 129.67, 129.62, 129.53, 129.09, 129.01, 128.92, 128.84, 128.74, 128.65, 128.36, 127.93, 127.84, 126.71, 126.24, 64.71, 64.39, 62.19, 62.06.

HRMS (ESI-ToF): calc. for [$C_{40}H_{35}O_4$] 579.25299 $[M+H]^+$, found 579.2530; calc. for [$C_{40}H_{34}NaO_4$] 601.2349 $[M+Na]^+$, found 601.2349.

3''-(2,5-bis(bromomethyl)phenyl)-2'''',3-bis(bromomethyl)-1,1':2',1'':4'',1''':3''',1''''-quinquephenyl (14)

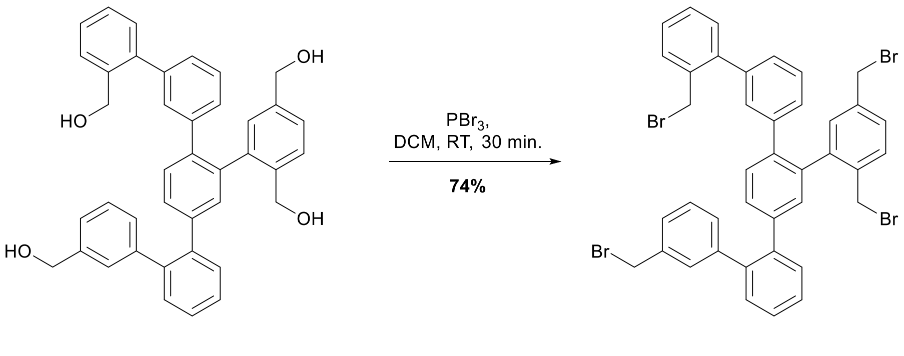

13 **14**

An oven dried and argon flushed Schlenk tube (50 mL) was charged with a solution of starting material **13** (372 mg, 643 µmol, 1.00 eq.) in dry DCM (35 mL) before adding PBr_3 (2.20 eq.). After

30 min complete consumption of the starting material could be observed by TLC. To the solvent was added Celite® and the solvent was removed under reduced pressure. The residue was purified by a short plug on neutral aluminium oxide (cyclohexane:ethyl acetate 20:1) to isolate the product **14** as a white solid (392 mg, 472 µmol, 74%).

Analytical data for 14:

^{1}H NMR (400 MHz, $CDCl_3$, 25 °C): δ = 7.57 – 7.54 (m, 1 H), 7.49 – 7.42 (m, 5 H), 7.37 (d, *J* = 1.9 Hz, 1 H), 7.36 – 7.34 (m, 1 H), 7.34 – 7.31 (m, 3 H), 7.31 – 7.28 (m, 2 H), 7.27 – 7.19 (m, 5 H), 7.11 (td, *J* = 1.8, 1.8, 0.6 Hz, 1 H), 7.05 (dd, *J* = 1.9, 0.4 Hz, 1 H), 6.97 (dd, *J* = 7.4, 1.6 Hz, 1 H), 6.89 (d, *J* = 1.9 Hz, 1H), 4.43 (q, *J* = 10.2, 10.2, Hz, 2 H), 4.33 (q, *J* = 10.2, 10.2 Hz, 2 H), 4.14 (d, *J* = 1.1 Hz, 2H), 4.05 (d, *J* = 10.4 Hz, 1 H), 3.88 (d, *J* = 10.4 Hz, 1 H) ppm.

^{13}C NMR (101 MHz, $CDCl_3$, 25 °C) δ = 142.0, 141.7, 141.3, 140.4, 140.1, 139.8, 139.7, 139.6, 138.9, 137.6 (2 C), 137.1, 135.4, 134.9, 132.6, 132.1, 131.1, 131.0, 130.5, 130.5, 130.5, 130.4, 130.3, 130.2, 129.9, 129.5, 128.7, 128.5, 128.3, 128.2, 128.1, 127.8, 127.8, 127.8, 127.3, 127.2, 33.5, 32.5, 32.3, 31.2 ppm.

DART-MS (500 °C, +): *m/z* (%) = 852.0 (20), 850.9 (21), 849.9 (66), 848.9 (26), 847.7 ($M+NH_4^+$, 100), 847.4 (14), 845.9 (64), 843.9 (17), 770.1 (32), 769.0 (18), 767.9 (71), 766.9 (16), 766.0 (58), 764.0 (17), 686.1 (11), 589.0 (16), 587.3 (15).

Analytical data can be found in reference[2].

---

2 M. Rickhaus, O. T. Unke, R. Mannancherry, L. M. Bannwart, M. Neuburger, D. Häussinger and M. Mayor, *Chem. - Eur. J.*, 2015, **21**, 18156–18167.

All-carbon Geländer system 1 and 2

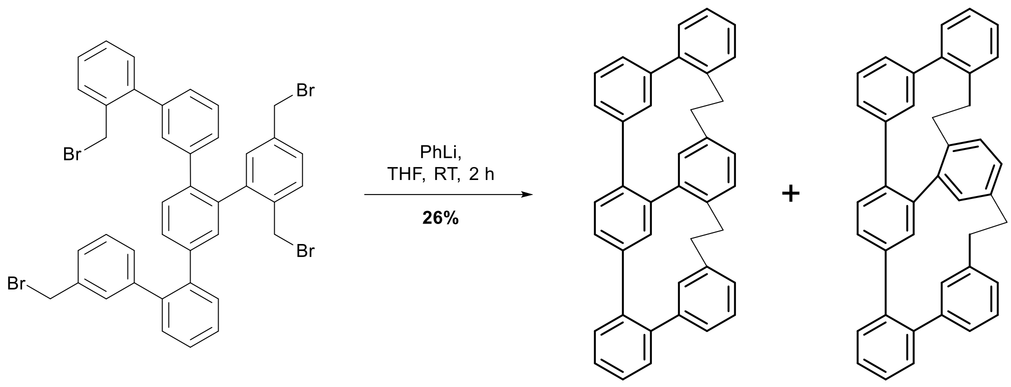

14 **1** **2**

An oven dried, argon flushed two-neck round bottom flask (25 mL) was equipped with droping funnel. The flask was charged with PhLi (1.8 M in Bu_2O, 165 µL, 3.00 eq) and freshly distilled dry THF (5 mL). The mixture was cooled down to 0 °C and then starting material **14** (82.0 mg, 98.8 µmol, 1.00 eq.) dissolved in freshly distilled dry THF (5 mL) was added dropwise to the solution via droping funnel over 15 min. After the addition, the solution was stirred at RT for 1 h. The reaction mixture was slowly quenched with H_2O (5 mL) and with a 1 M aqueous solution of HCl (5 mL). The aqueous phase was washed with EtOAc (3 x 10 mL). The combined organic layers were washed with H_2O (30 mL), brine (30 mL) and dried over Na_2SO_4. After filtration, the solvent was removed under reduced pressure. The product (**1** and **2**, 13.3 mg, 26.0 µmol, 26%) was isolated by column chromatography (SiO_2, cyclohexane:toluene, 4:1) as a white solid. The constitutional isomers **1** and **2** were separated by HPLC on a reversed stationary phase (Preparative Reprosil C18 column, eluent: MeCN, flow rate: 100 mL min^{-1}, T = 25 °C). Further separation of the corresponding enantiomers were achieved by HPLC on a chiral stationary phase (Analytical Chiralpak® IA column, eluent: *n*-hexane:*i*-PrOH 99:1, flow rate: 1 mL min^{-1}, T = 18 °C).

Analytic data for 1:

^{1}H NMR (600 MHz, Toluene-d_8, 25 °C) δ = 7.71 (d, *J* = 8.2 Hz, 1H), 7.60 (dd, *J* = 8.1, 2.2 Hz, 1H), 7.46 (dd, *J* = 7.5, 1.5 Hz, 1H), 7.28 (dtd, *J* = 7.6, 3.5, 1.6 Hz, 2H), 7.22 (td, *J* = 7.4, 1.4 Hz, 1H), 7.21 – 7.17 (m, 2H), 7.16 – 7.12 (m, 2H), 7.05 (dd, *J* = 7.4, 1.4 Hz, 1H), 6.99 – 6.97 (m, 1H), 6.95 – 6.90 (m, 2H), 6.87 (dt, *J* = 7.3, 1.3 Hz, 1H), 6.73 (d, *J* = 7.8 Hz, 1H), 6.62 (dt, *J* = 7.1, 1.6 Hz, 1H), 6.50 (dd, *J* = 7.9, 1.9 Hz, 1H), 6.34 (dd, *J* = 11.6, 2.0 Hz, 2H), 5.29 (t, *J* = 1.7 Hz, 1H), 2.76

(dt, J = 13.6, 3.9 Hz, 1H), 2.59 (dt, J = 13.0, 4.1 Hz, 1H), 2.46 (tdd, J = 13.2, 8.9, 3.9 Hz, 3H), 2.25 (dt, J = 13.9, 3.7 Hz, 1H), 2.10 – 2.05 (m, 1H), 2.05 – 1.96 (m, 1H) ppm.

^{13}C NMR (151 MHz, Toluene-d_8, 25 °C) δ = 143.15, 141.99, 140.76, 140.75, 140.29, 139.44, 139.38, 138.67, 138.57, 138.17, 137.99, 137.75, 137.52, 137.33, 137.25, 131.64, 131.56, 130.85, 129.82, 129.69, 129.31, 129.10, 128.91, 128.87, 128.76, 128.14, 127.95, 127.88, 127.80, 127.65, 127.55, 126.92, 126.91, 126.06, 125.10, 36.35, 36.19, 33.32, 32.36, 20.84 ppm.

DART-MS (450 °C, +): *m/z* (%) = 529.1 (10), 528.1 ($M+NH_4^+$, 29), 526.1 (11), 525.1 (15), 512.2 (25), 511.1 ($M+H^+$, 100), 510.0 (48), 278.8 (12).

HRMS (EI, +): calc. for [$C_{40}H_{30}$] 510.23475 [M], found 510.23301.

Analytic data for 2:

^{1}H NMR (600 MHz, Toluene-d_8, 25 °C) δ = 7.44 (d, J = 7.6 Hz, 1H), 7.37 (s, 1H), 7.35 (d, J = 6.8 Hz, 1H), 7.28 – 7.22 (m, 3H), 7.21 – 7.12 (m, 5H), 7.00 – 6.94 (m, 4H), 6.91 – 6.88 (m, 3H), 6.87 – 6.81 (m, 3H), 6.71 – 6.65 (m, 2H), 5.54 (s, 1H), 3.08 – 2.99 (m, 1H), 2.92 (t, J = 12.1 Hz, 1H), 2.84 – 2.76 (m, 1H), 2.74 – 2.65 (m, 2H), 2.58 (ddd, J = 13.7, 5.0, 2.5 Hz, 1H), 2.21 (t, J = 12.7 Hz, 1H), 1.74 – 1.66 (m, 1H) ppm.

^{13}C NMR (151 MHz, Toluene-d_8, 25 °C) δ = 144.42, 144.42, 143.43, 142.97, 142.67, 141.69, 141.66, 141.46, 140.83, 140.72, 140.46, 140.04, 139.39, 138.22, 137.18, 135.05, 130.60, 129.24, 129.24, 128.99, 128.60, 128.44, 128.00, 127.81, 127.76, 127.71, 127.53, 127.46, 127.33, 126.95, 126.66, 126.66, 126.07, 125.83, 125.53, 125.02, 39.10, 37.50, 36.80, 35.80 ppm.

DART-MS (450 °C, +): *m/z* (%) = 530.1 (8), 529.2 (28), 528.2 ($M+NH_4^+$, 100), 512.1 (10), 511.0 ($M+H^+$, 34), 510.1 (6), 391.0 (11), 278.8 (8).

HRMS (EI, +): calc. for [$C_{40}H_{30}$] 510.23475 [M], found 510.23339.

HPLC Chromatograms of Geländer System 1 and 2

High-performance liquid chromatography (HPLC) using a reversed stationary phase (Reprosil C18, MeCN, 100 mL min^{-1}, 25 °C) allowed the separation of ***rac*-1** and ***rac*-2**. Furthermore both structural isomers could be successfully baseline separated into their enantiomers **1a** and **1b** respectively into **2a** and **2b** by HPLC using a chiral stationary phase (Figure S1; Chiralpak IA, 1 mL min-1, 99:1 *n*-hexane:*i*-PrOH, 18 °C).

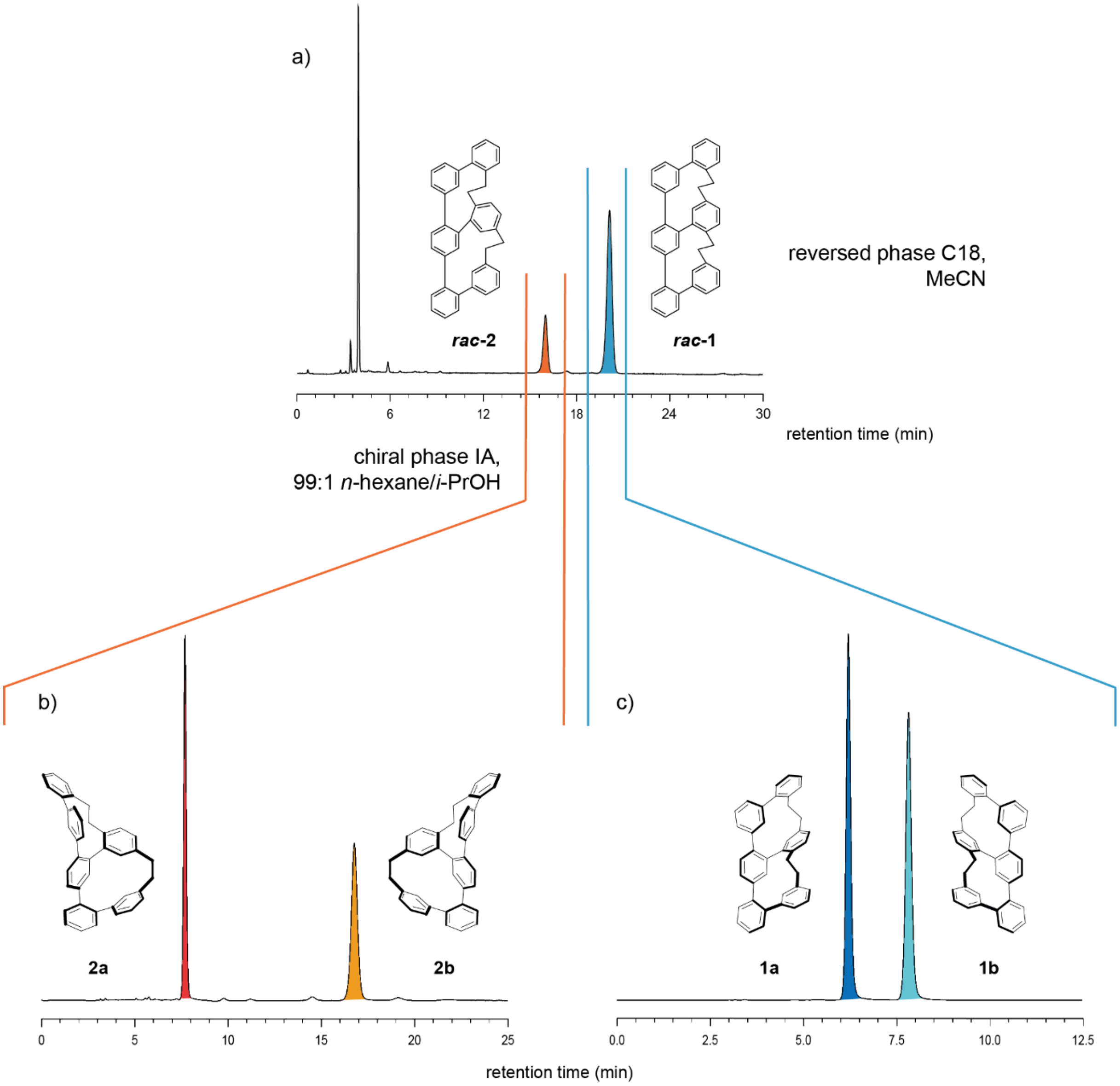

Figure S1: HPLC chromatograms of reversed (Reprosil C18, MeCN, 100 mL min^{-1}, 25 °C) and chiral (Chiralpak IA, 1 mL min^{-1}, 99:1 *n*-hexane:*i*-PrOH, 18 °C) stationary phases. a) Separation of the regioisomers of **1** (blue) and **2** (orange) on a reversed stationary phase with MeCN as eluent. The ratio of ***rac*-1** to ***rac*-2** is 3:1. b) Separation of both enantiomers of **2a** (red) and **2b** (orange) and c) separation of enantiomers **1a** (blue) and **1b** (turquoise) on a chiral stationary phase in a mixture of 99:1 *n*-hexane and *i*-PrOH.

NMR studies of Geländer System 1 and 2

a) b) c) d)

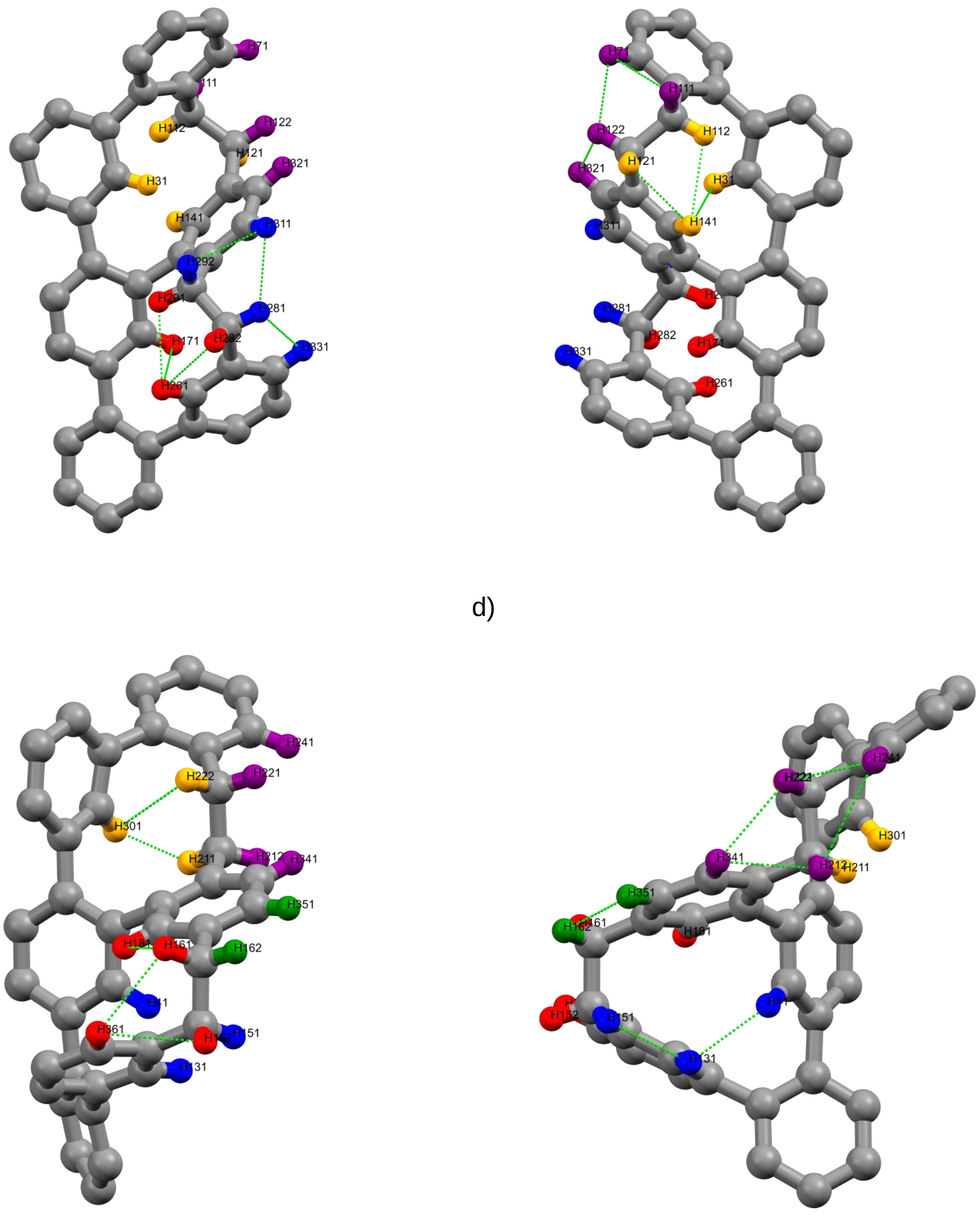

Figure S2: NOE relationships of Geländer a), b) **1** and c),d) **2** from different viewpoints - a) and c) front view and b) and d) back view. Five important hydrogen groups colored in red, blue, purple, yellow and green. The dashed green lines visualize the trough space couplings and explain the configuration of each ethyl bridge.

$^{1}H,^{1}H$-NOESY experiment particularly showed four important areas for **1** and five areas for **2** of through space relationships (Figure S2a-d; highlighted in blue, red, yellow, purple and green) which lashes the ethyl protons in the *syn*/*syn* conformation for helix **1** and for **2** in the *syn*/*anti* conformation (Figure S2a-d; Nuclear Overhauser effect (NOE) relationships highlighted in green dashed lines).

The *syn* configuration of the bottom ethyl bridge in **1** can be explained by the NOE relationships of the protons coloured in red and blue. The α protons H282 and H291 in red have only a NOE to proton H261 (red). The β protons H281 and H292 in blue show a trough space coupling to proton H311 (blue) and additionally, as a cross-check, H281 interacts with H331 (blue) too. The top bridge with a *syn* configuration is explained by the yellow and purple coloured hydrogens. The α protons H111 and H122 in purple show a NOE with the same hydrogen atom H71 (purple) but only H321 (purple) additionally interacts with H122. The β protons H112 and H121 in yellow are coupling with the central benzene unit proton H141 (yellow) of the banister part. These NOE relationships tighten both ethyl bridges in the *syn* arrangement.

The *syn* configuration of the bottom ethyl bridge in **2** is explained by the NOE relationships of the protons coloured in red, blue and green. The α protons H156 and H161 in red have a NOE with H261 (red) and in addition H161 couples with H181 too. The β hydrogen, H151 in blue, shows through space coupling to backbone proton H41 (blue) on the central benzene core and proton H131 (blue) on the neighbouring phenyl unit. The other β proton H162 in green interacts only with the central benzene unit hydrogen H351 (green) of the banister system. The top bridge with the *anti* configuration is described by the yellow and purple coloured hydrogens. Each of the α proton H212 and H221 (purple) have a through space coupling to protons H241 (purple) and H341 (purple). In addition the β hydrogens H211 and H222 in yellow show a NOE to proton H301 (yellow) on the opposite phenyl group. These through space relations lash the bottom ethyl bridge in the *syn* and the top bridge in the *anti* conformation.

Finally, NMR spectroscopy confirmed the structural arrangement of both macrocycles **1** and **2** to be the same as previously elucidated by X-ray crystallography.

1 in toluene-d_8 COSY

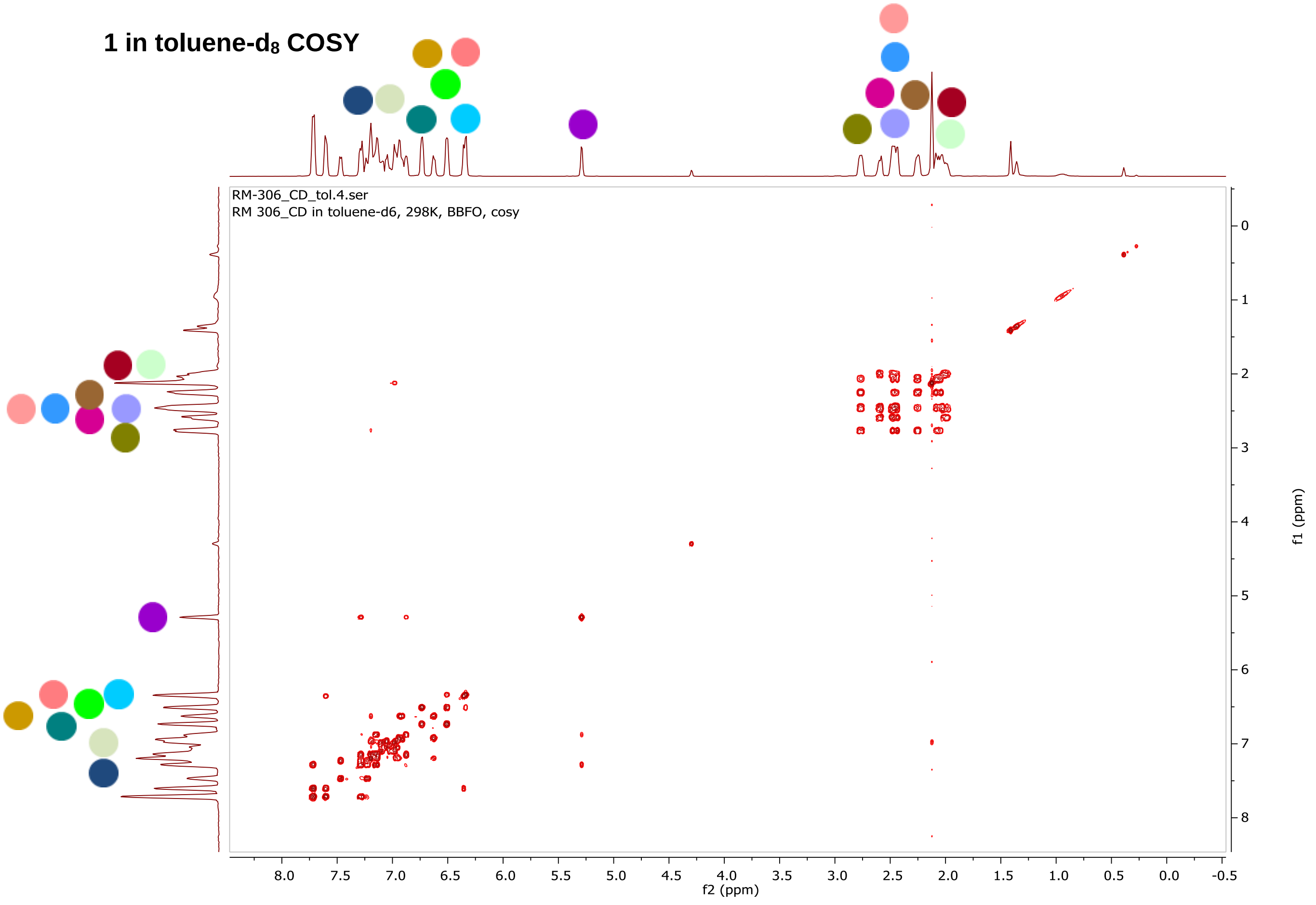

H31
H71
H111
H112
H121
H122
H141
H171
H261
H281
H282
H291
H292
H311
H321
H331

1 in toluene-d$_8$ NOESY

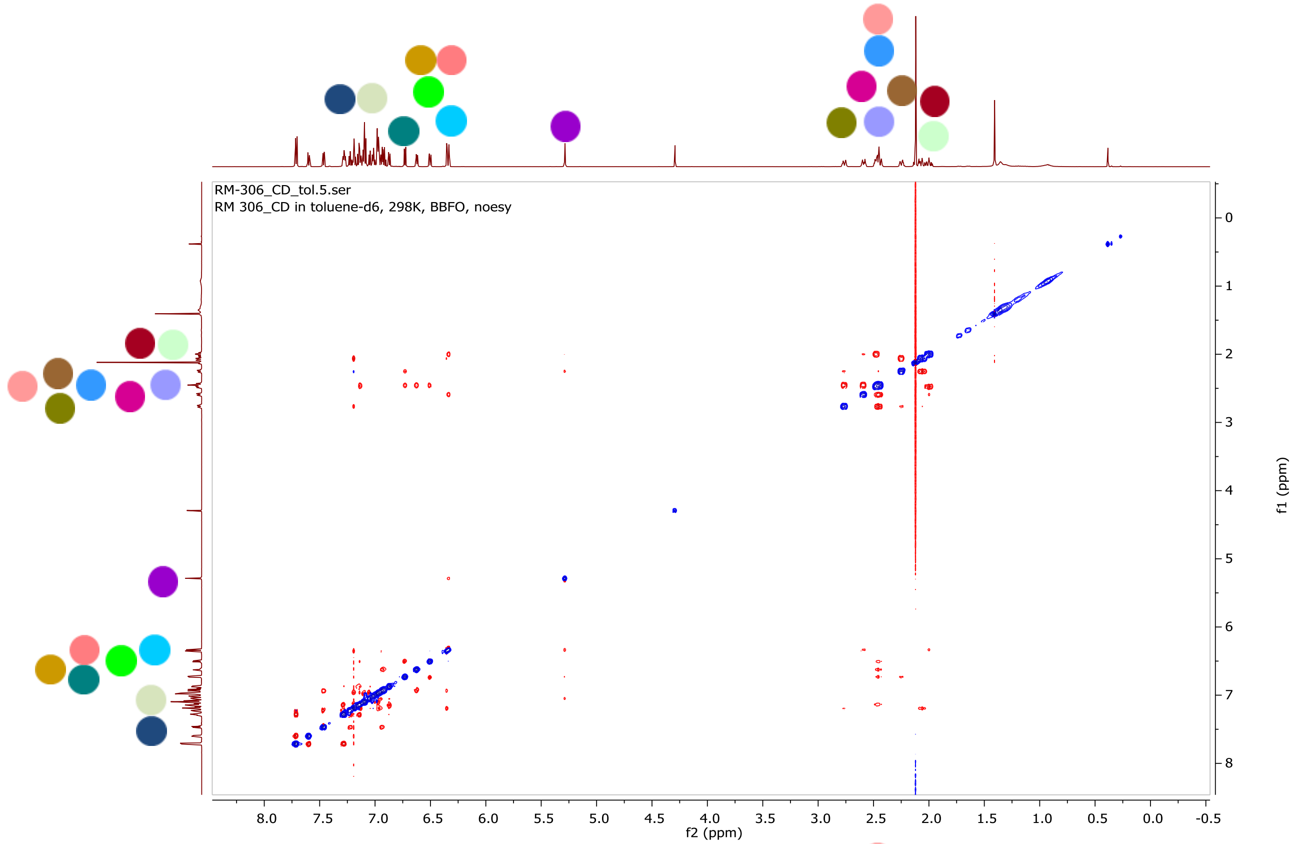

1 in toluene-d$_8$ TOCSY

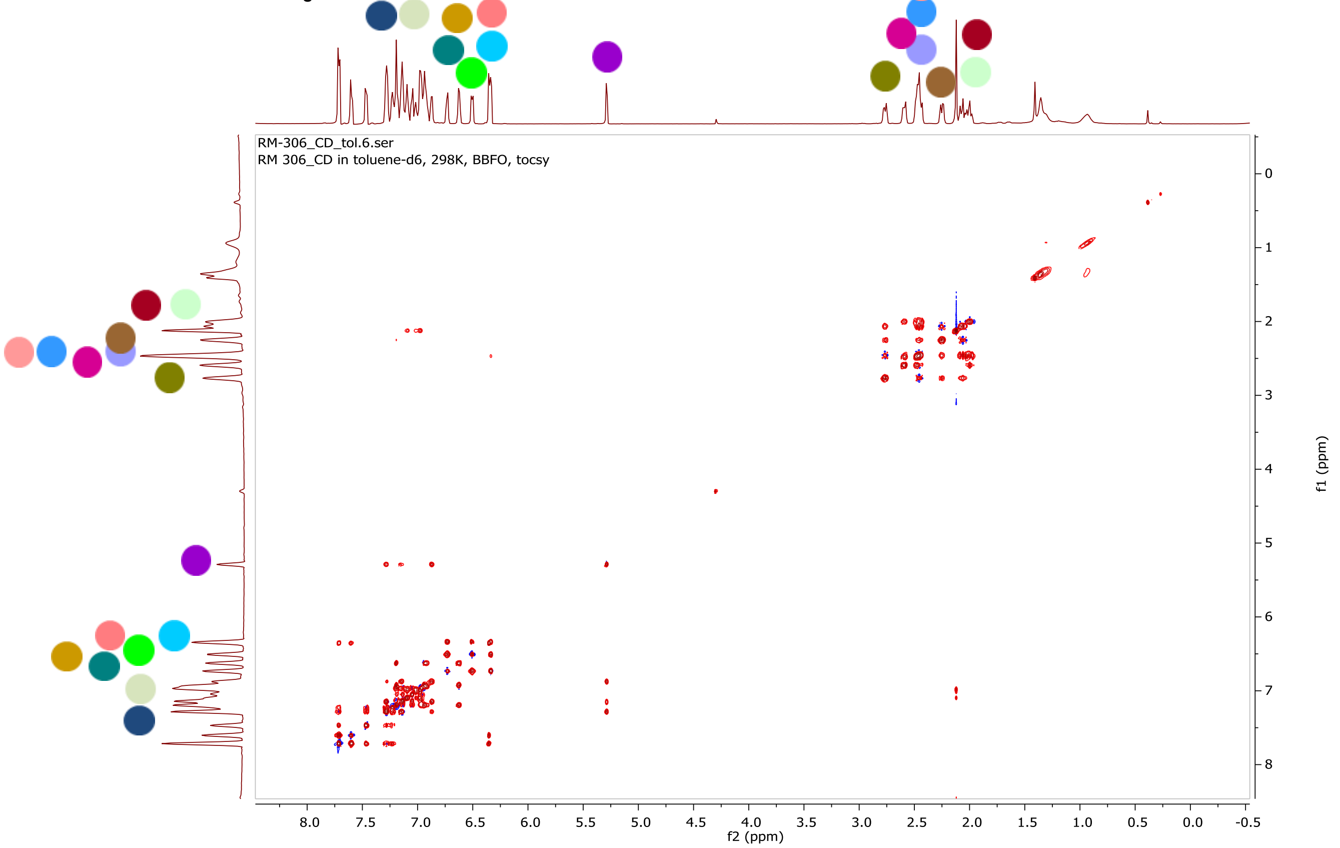

1 in toluene-d$_8$ HMBC

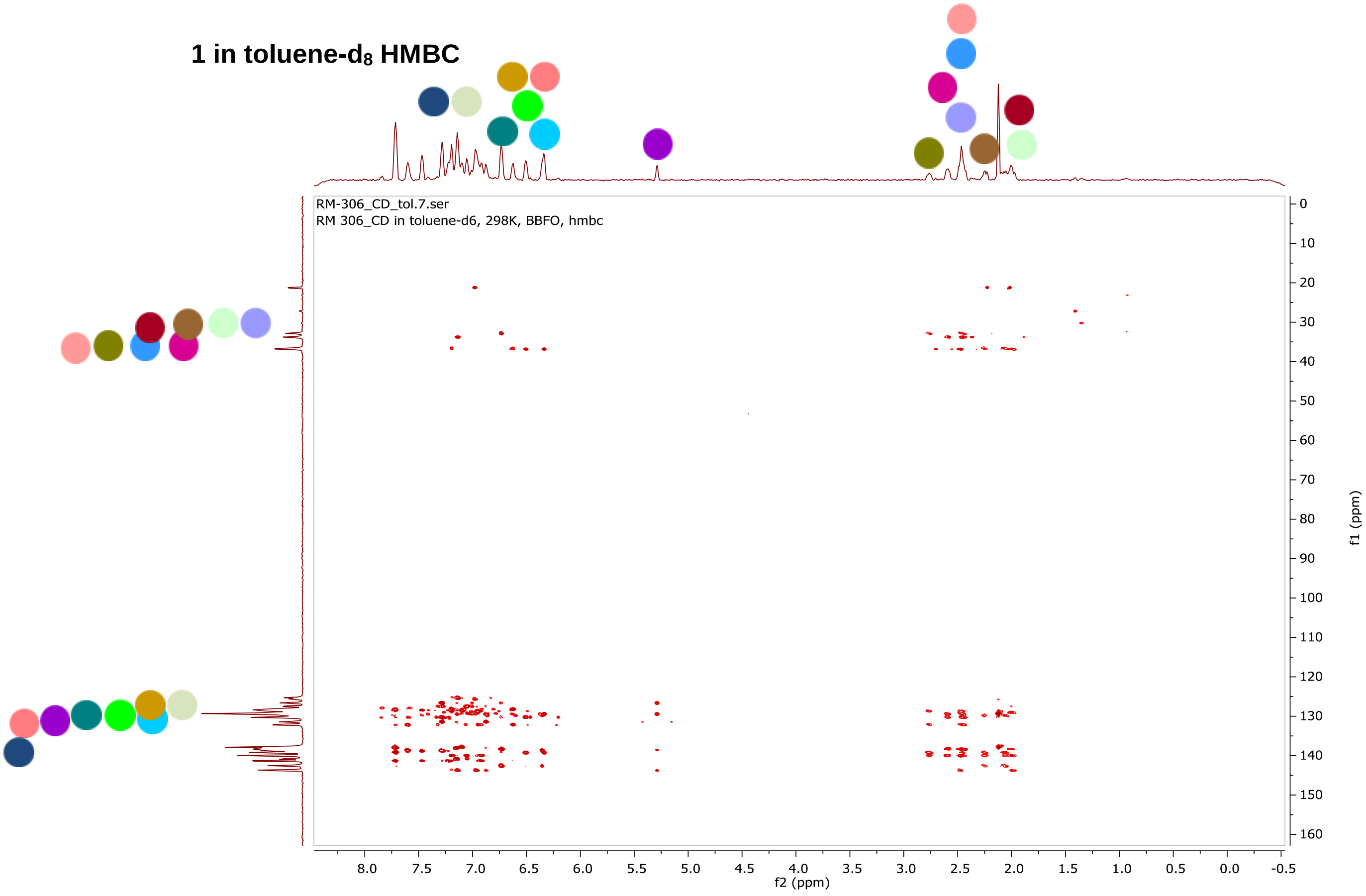

1 in toluene-d$_8$ HMQC

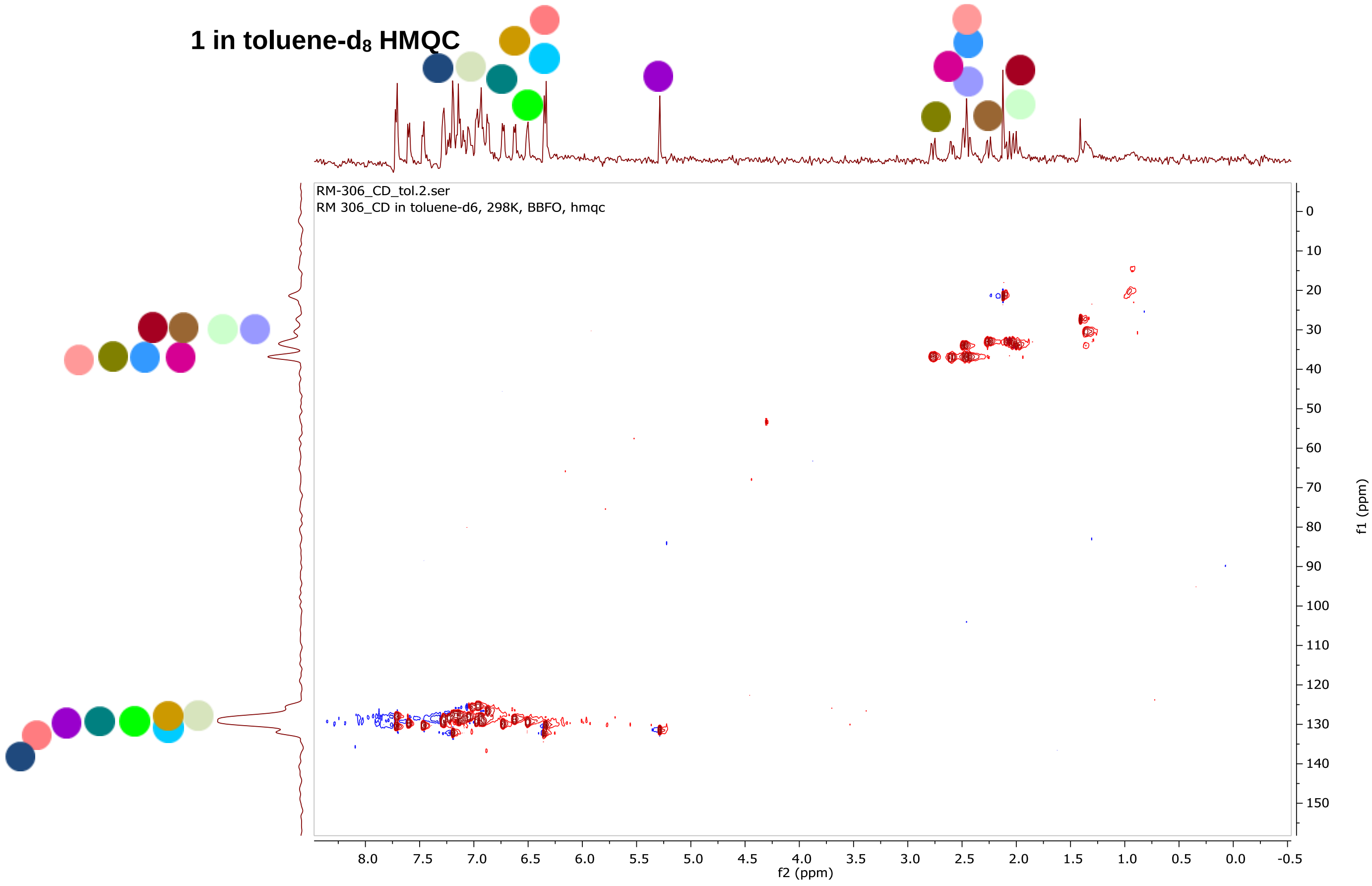

2 in toluene-d_8 COSY

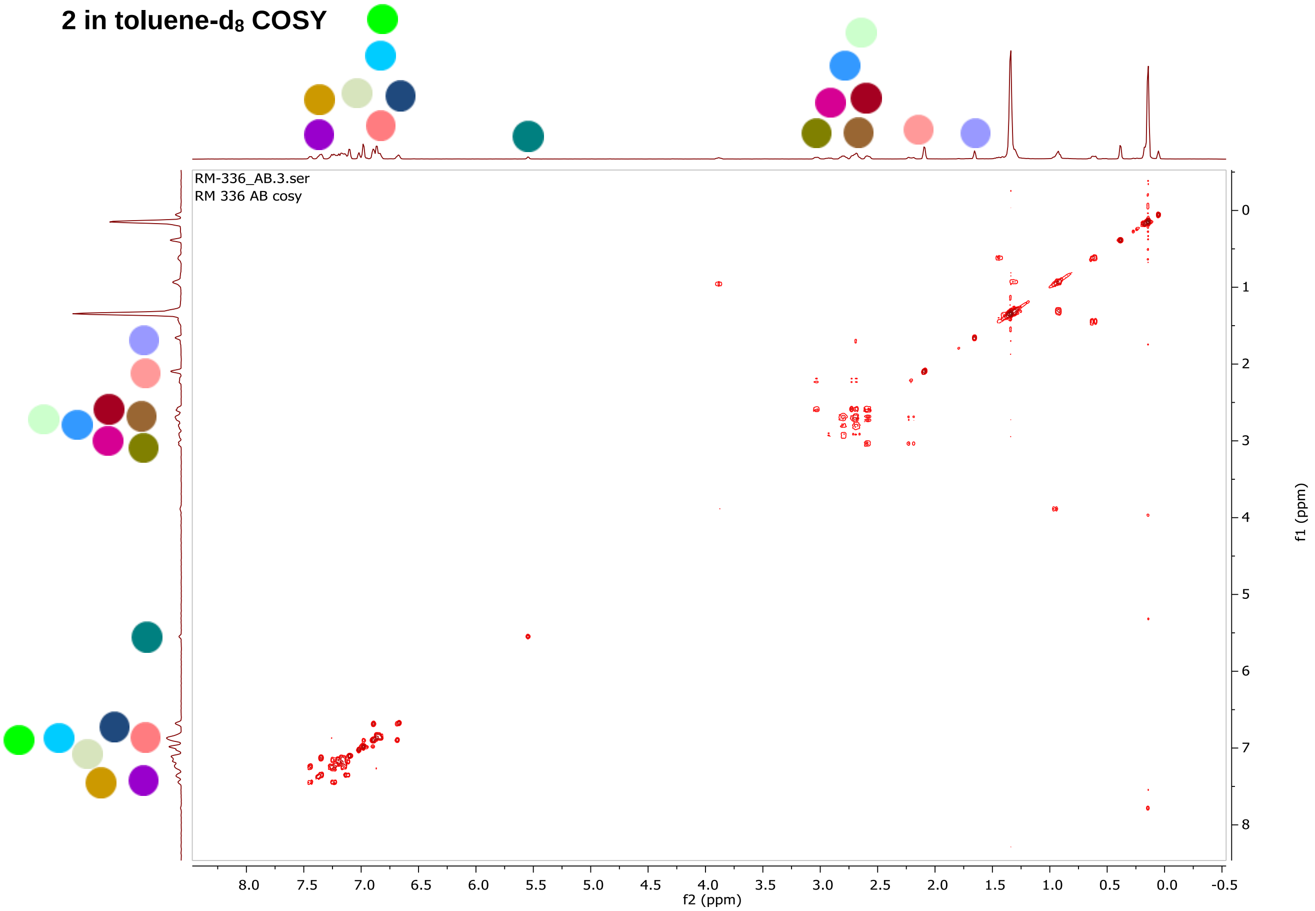

- H41
- H131
- H151
- H152
- H161
- H162
- H181
- H211
- H212
- H221
- H222
- H241
- H301
- H341
- H351
- H361

2 in toluene-d_8 NOESY

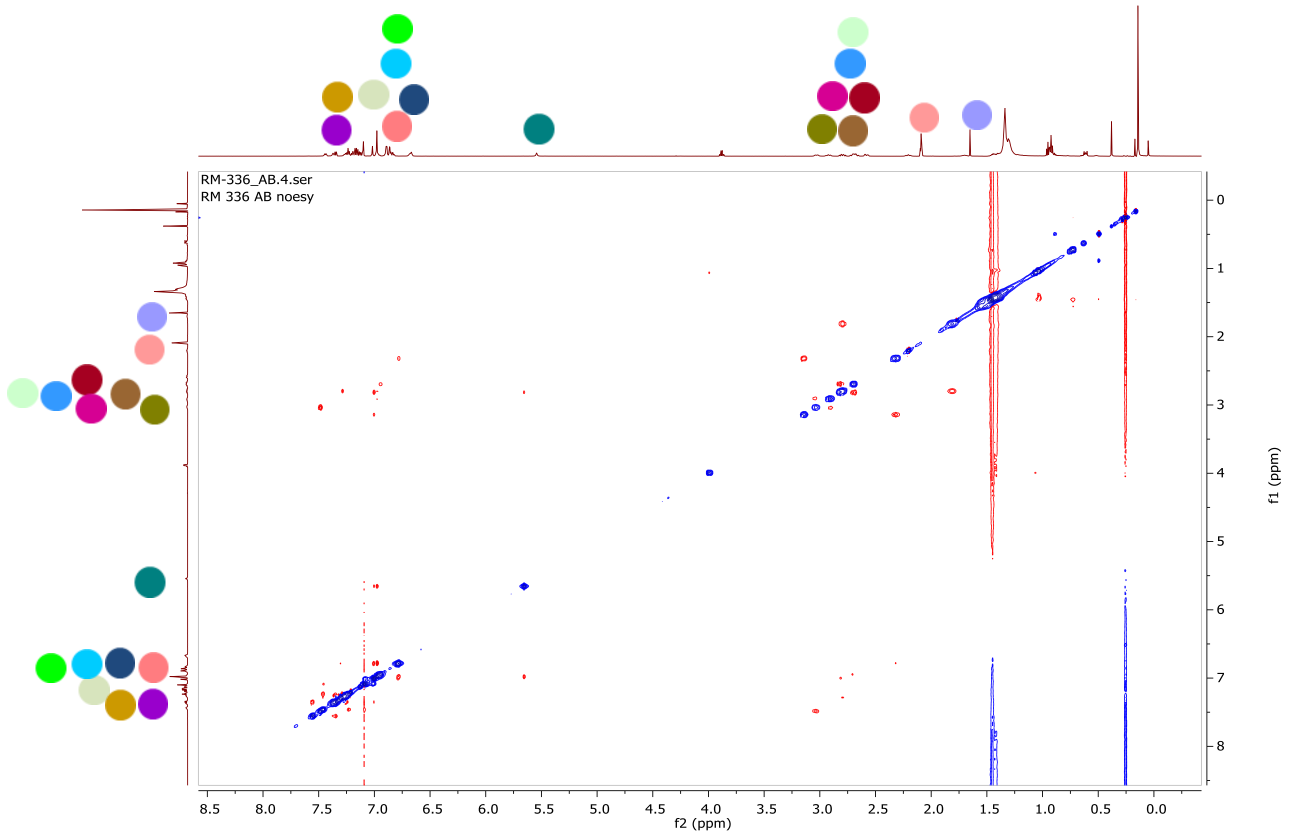

2 in toluene-d_8 TOCSY

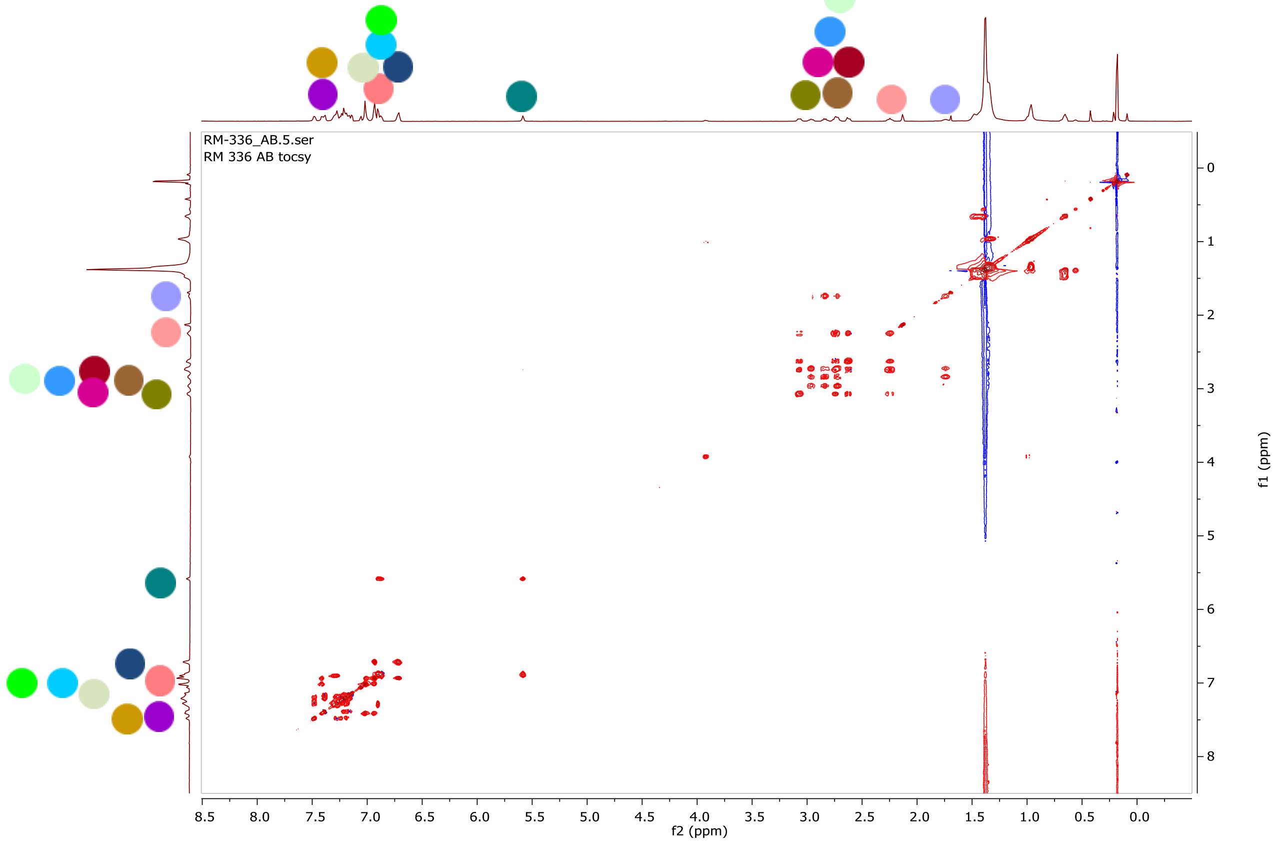

2 in toluene-d_8 HMBC

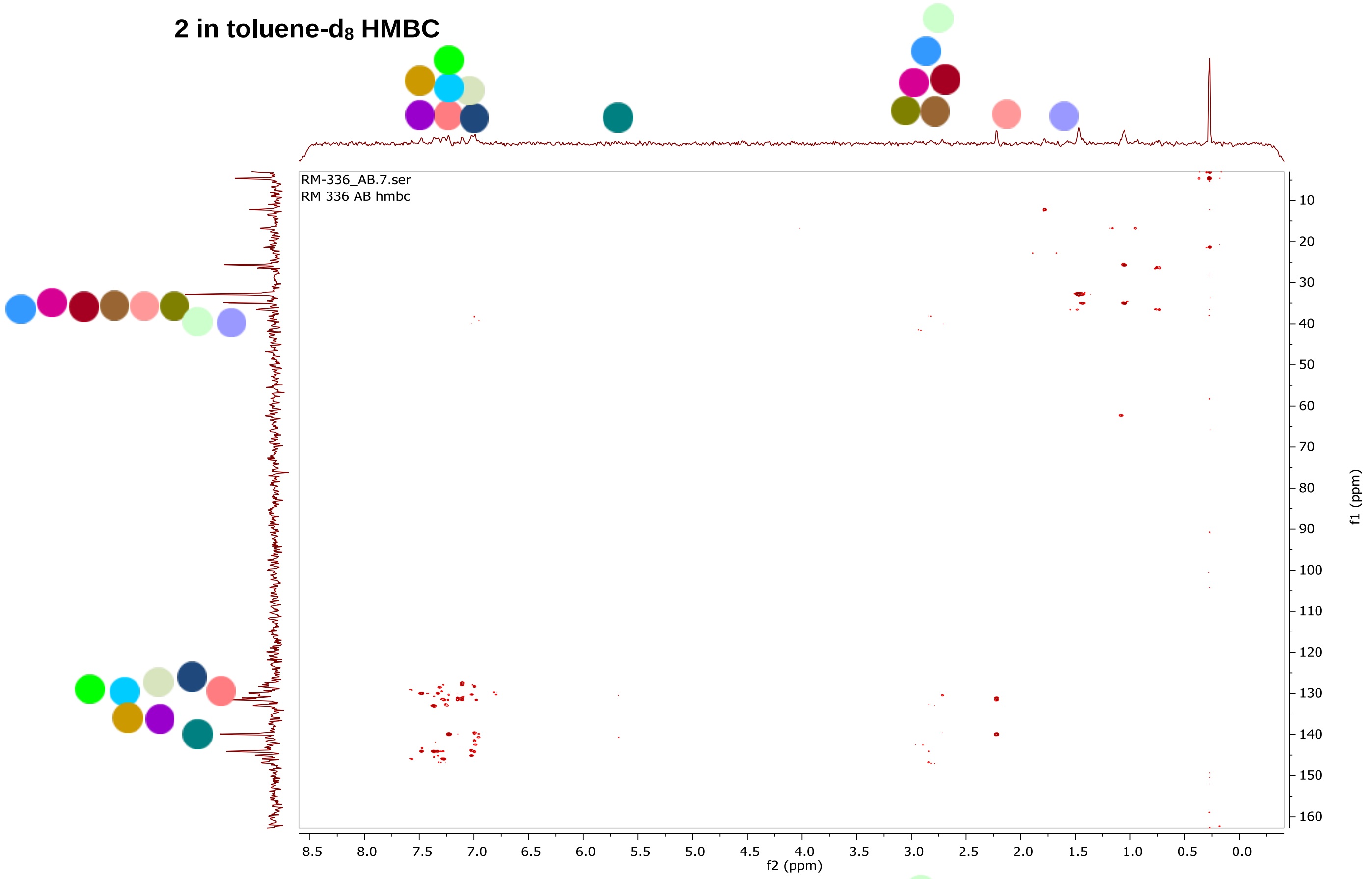

2 in toluene-d_8 HMQC

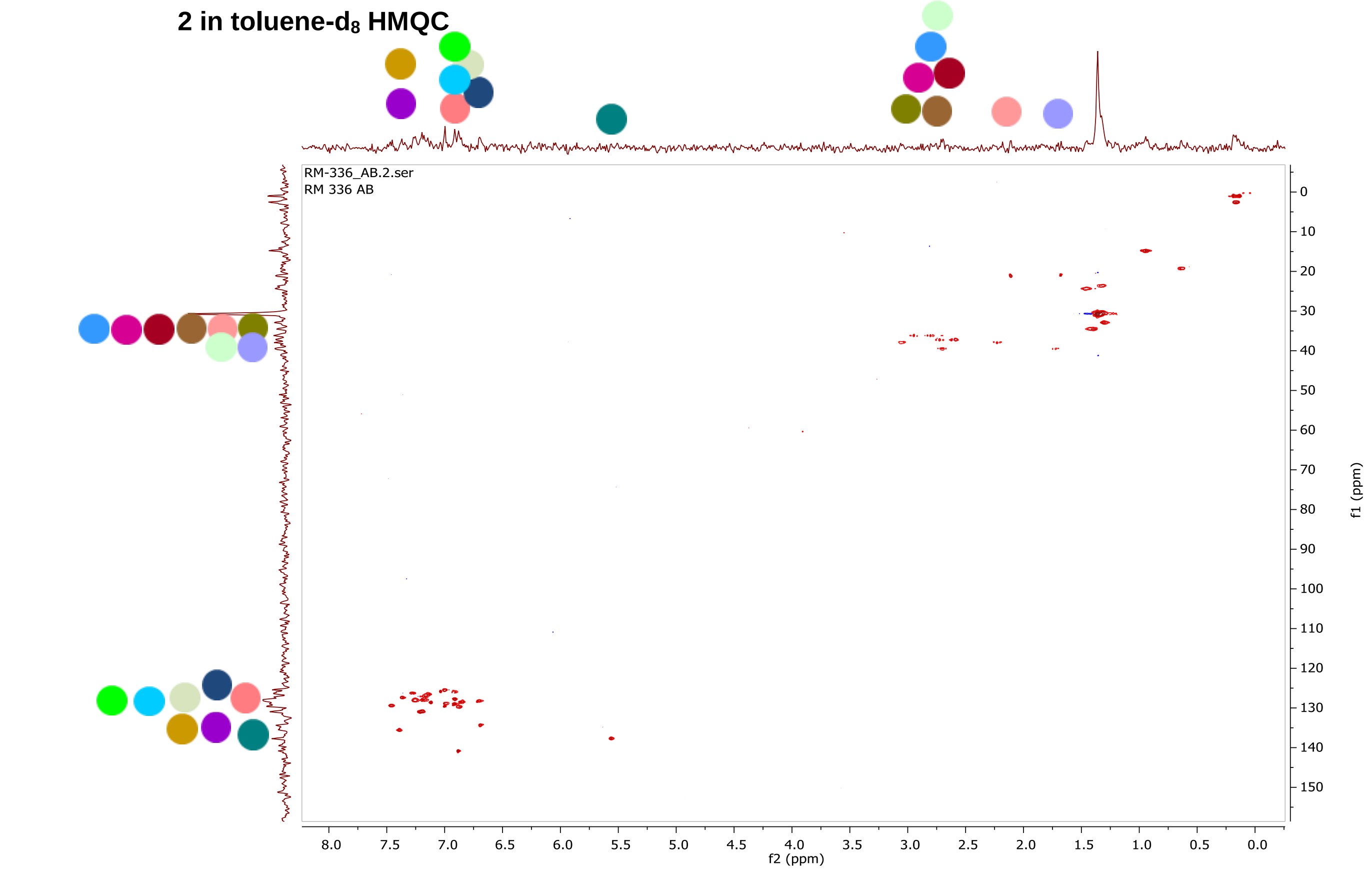

Single Crystal X-Ray Analysis of 1

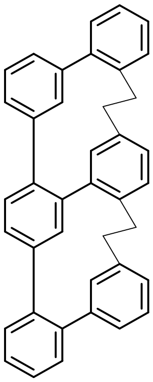

1

Crystals of **1** were grown from *n*-hexane:*i*-PrOH (99:1) by slow vapor diffusion technique with DCM at room temperature. Formula $C_{40}H_{30}$, M = 510.68, F(000) = 1080, colourless block, size 0.050 x 0.080 x 0.100 mm^3, monoclinic, space group P 21/c , Z = 4, a = 8.5388(5) Å, b = 14.2697(8) Å, c = 22.0284(14) Å, α = 90°, β = 94.249(3)°, γ = 90°, V = 2676.7(3) $Å^3$, Dcalc. = 1.267 Mg * m^{-3}. The crystal was measured on a Bruker Kappa Apex2 diffractometer at 123K using graphite-monochromated Cu Kα-radiation with λ = 1.54178 Å, Θmax = 68.977°. Minimal/maximal transmission 0.88/0.97, μ = 0.540 mm^{-1}. The Apex2 suite has been used for datacollection and integration. From a total of 38356 reflections, 4895 were independent (merging r = 0.036). From these, 4875 were considered as observed (I>2.0σ(I)) and were used to refine 361 parameters. The structure was solved by other methods using the program Superflip. Least-squares refinement against F^2 was carried out on all non-hydrogen atoms using the program CRYSTALS. R = 0.0352 (observed data), wR = 0.0781 (all data), GOF = 0.9378. Minimal/maximal residual electron density = -0.22/0.22 e $Å^{-3}$. Chebychev polynomial weights were used to complete the refinement. Plots were produced using CAMERON. Crystallographic data (excluding structure factors) for the structure **1** in this paper have been deposited with the Cambridge Crystallographic Data Center, the deposition number is (1811810). Copies of the data can be obtained, free of charge, on application to the CCDC, 12 Union Road, Cambridge CB2 1EZ, UK [fax: +44-1223-336033 or e-mail: deposit@ccdc.cam.ac.uk].

Single Crystal X-Ray Analysis of 2

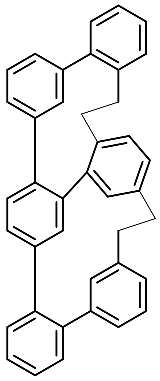

2

Crystals of **2** were grown from *n*-hexane:*i*-PrOH (99:1) by slow vapor diffusion technique with DCM at room temperature. Formula $C_{40}H_{30}$, M = 510.68, F(000) = 1080, colourless needle, size 0.050 x 0.080 x 0.100 mm^3, monoclinic, space group P 21/c , Z = 4, a = 9.2297(4) Å, b = 10.6903(4) Å, c = 27.8883(12) Å, α = 90°, β = 91.6399(18)°, γ = 90°, V = 2750.6(2) Å^3, Dcalc. = 1.233 Mg * m^{-3}. The crystal was measured on a Bruker Kappa Apex2 diffractometer at 123K using graphite-monochromated Cu Kα-radiation with λ = 1.54178 Å, Θmax = 70.194°. Minimal/maximal transmission 0.89/0.97, μ = 0.526 mm^{-1}. The Apex2 suite has been used for datacollection and integration. From a total of 28502 reflections, 5086 were independent (merging r = 0.030). From these, 5068 were considered as observed (I>2.0σ(I)) and were used to refine 361 parameters. The structure was solved by other methods using the program Superflip. Least-squares refinement against F^2 was carried out on all non-hydrogen atoms using the program CRYSTALS. R = 0.0361 (observed data), wR = 0.0785 (all data), GOF = 0.9187. Minimal/maximal residual electron density = -0.16/0.29 e Å^{-3}. Chebychev polynomial weights were used to complete the refinement. Plots were produced using CAMERON. Crystallographic data (excluding structure factors) for the structure **2** in this paper have been deposited with the Cambridge Crystallographic Data Center, the deposition number is (1811809). Copies of the data can be obtained, free of charge, on application to the CCDC, 12 Union Road, Cambridge CB2 1EZ, UK [fax: +44-1223-336033 or e-mail: deposit@ccdc.cam.ac.uk].

Determination of torsion angles of macrocycles 1 and 2

The torsional angles of each biphenyl unit in the backbone of the zig-zag system **1** and of the helical system **2** were determined by measuring the two corresponding planes. The plane was defined by the mean of the six carbon atoms of each benzene unit (For **1**: 32.50° and 37.56°; for **2**: 70.81° and 161.19°).

The overall torsion angle of the diminished helical system **1** was determined by the centroids between the methyl bonds of the *para*-xylene unit and together with the centroids of the two external benzene units of the banister core. Thereby the overall torsion angle of 177.60° was measured.

The overall torsion angle for Geländer system **2** was determined by the centroids of the two external benzene units of the banister core and with the 4 and 4'' carbons of the terphenyl backbone. The overall torsion angle of 126.37° was found.

Crystal packing of 1 and 2

Both samples ***rac*-1** and ***rac*-2** were crystallized from *n*-hexane:*i*-PrOH (99:1) by slow vapor diffusion with DCM. The unit cells are racemic for both **1** and **2** and the enantiomers are present in a 2:2 ratio.

a) b)

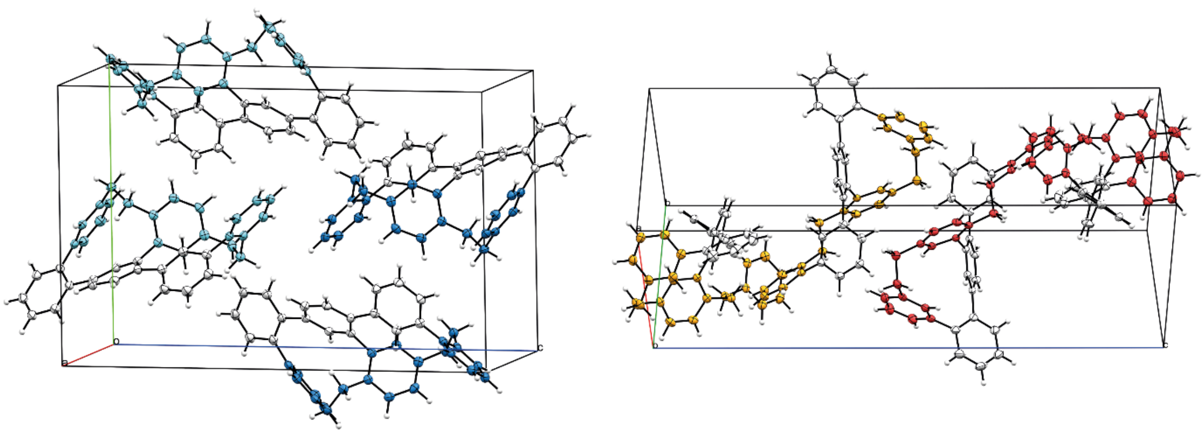

Figure S3: The unit cell of a) ***rac*-1** (blue, turquoise) and b) ***rac*-2** (red, orange), which contains two molecules of each enantiomer. Color code: Bridge = blue, turquoise red and orange, backbone = gray, hydrogen atoms = white.

^{1}H-, ^{13}C-NMR ($CDCl_3$, 400/101 MHz, 25 °C) and HR-ESI spectra of compound 9

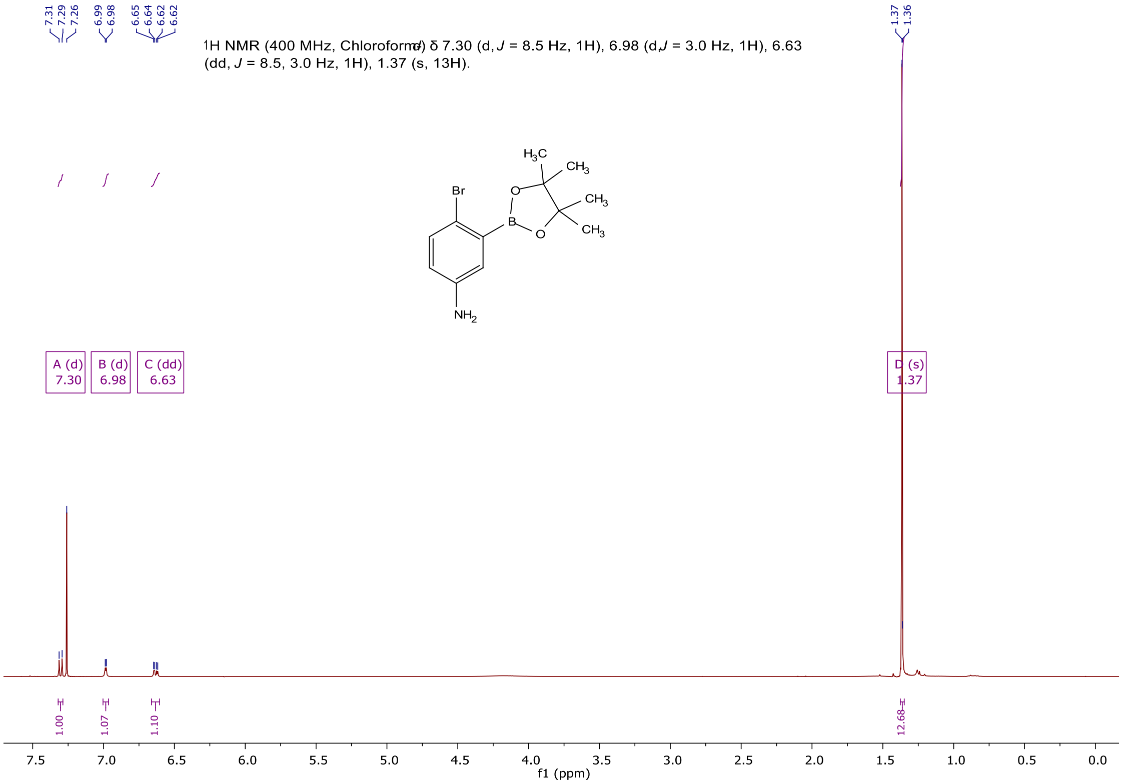

^{1}H NMR (400 MHz, Chloroform-*d*) δ 7.30 (d, *J* = 8.5 Hz, 1H), 6.98 (d, *J* = 3.0 Hz, 1H), 6.63 (dd, *J* = 8.5, 3.0 Hz, 1H), 1.37 (s, 13H).

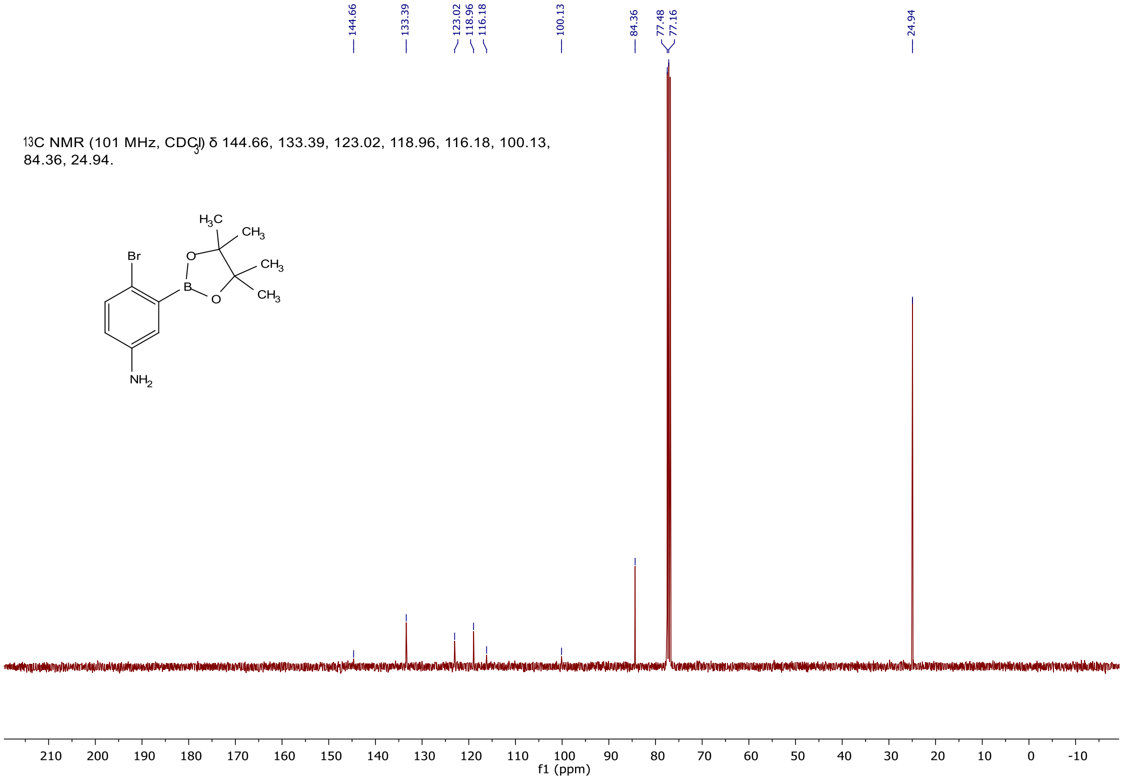

^{13}C NMR (101 MHz, $CDCl_3$) δ 144.66, 133.39, 123.02, 118.96, 116.18, 100.13, 84.36, 24.94.

Mass Spectrum SmartFormula Report

Analysis Info

Analysis Name: N:\new acq data\RM089 001.d
Method: hn Direct_Infusion_pos mode_75-1700 mid 4eV.m
Sample Name: Rajesh Mannancherry, RM089
Comment: ca 1.2 ug/mL MeCN

Acquisition Date: 16.07.2015 08:45:57
Operator: hn
Instrument / Ser#: maXis 4G 21243

Acquisition Parameter

Source Type	ESI	Ion Polarity	Positive	Set Nebulizer	0.4 Bar
Focus	Not active	Set Capillary	3600 V	Set Dry Heater	180 °C
Scan Begin	75 m/z	Set End Plate Offset	-500 V	Set Dry Gas	4.0 l/min
Scan End	1700 m/z	Set Collision Cell RF	500.0 Vpp	Set Ion Energy (MS only)	4.0 eV

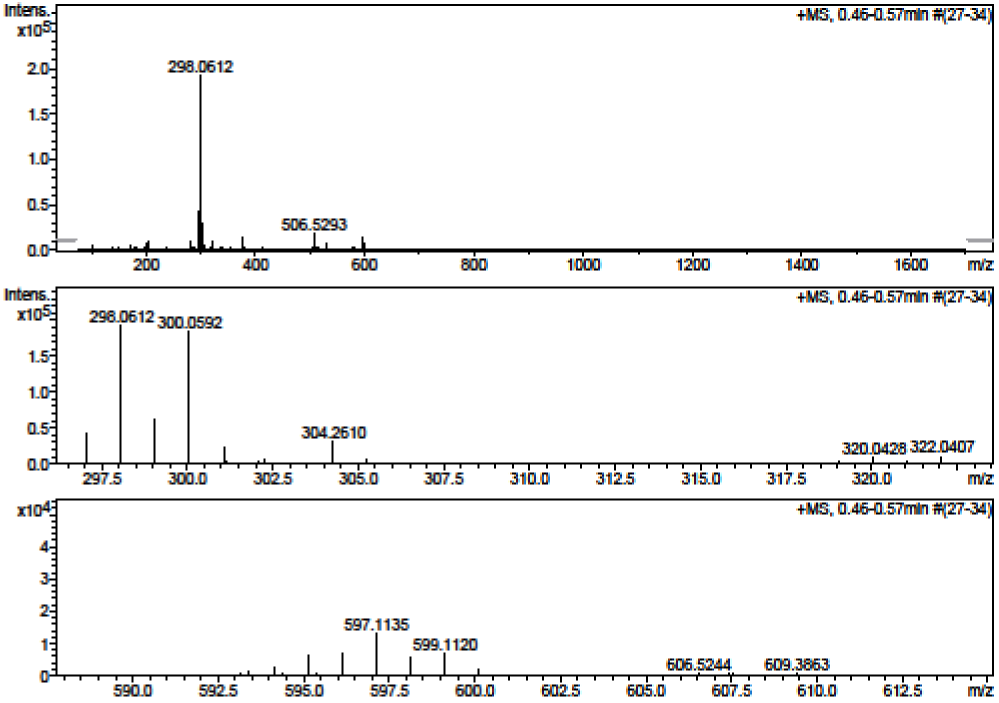

Meas. m/z	#	Formula	Score	m/z	err [mDa]	err [ppm]	mSigma	rdb	e⁻ Conf	N-Rule	z
298.0612	1	C 12 H 18 B Br N O 2	100.00	298.0611	-0.2	-0.5	23.3	4.5	even	ok	1+
320.0428	1	C 12 H 17 B Br N Na O 2	100.00	320.0430	-0.0	-0.1	19.6	4.5	even	ok	
595.1155	1	C 24 H 35 B 2 Br 2 N 2 O 4	100.00	595.1146	-0.9	-1.5	25.6	8.5	even	ok	

^{1}H-, ^{13}C-NMR (CDCl$_3$, 400/101 MHz, 25 °C) and HR-ESI spectra of compound 10

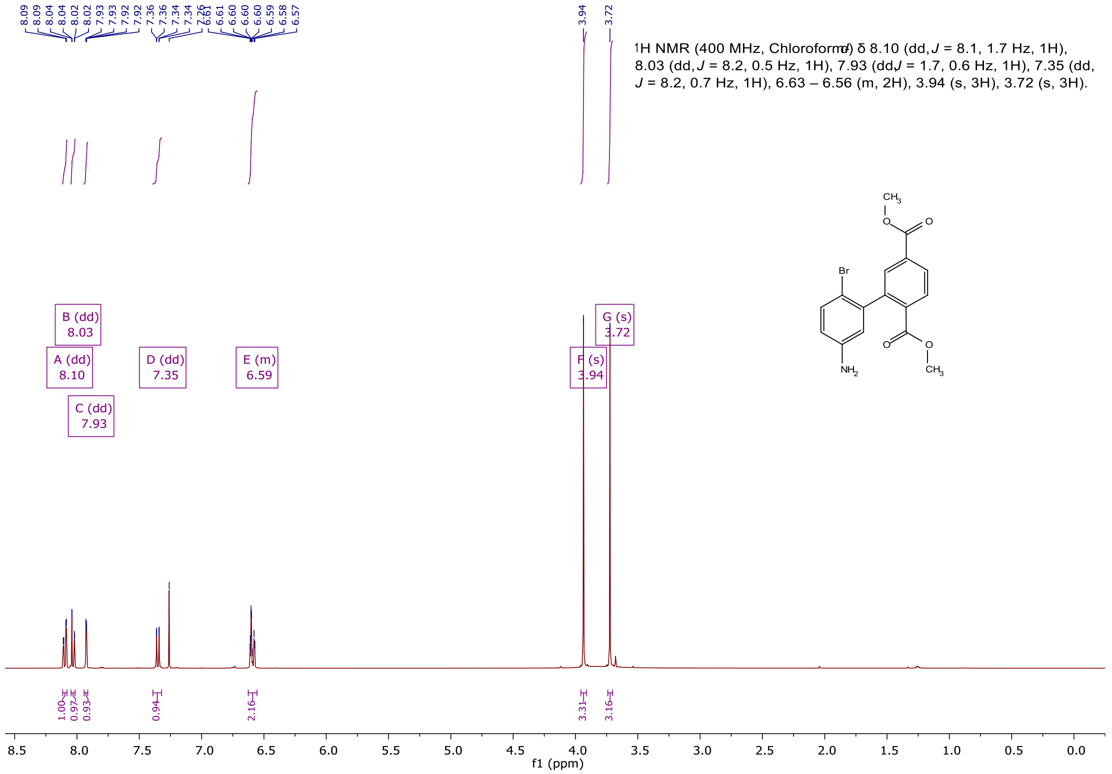

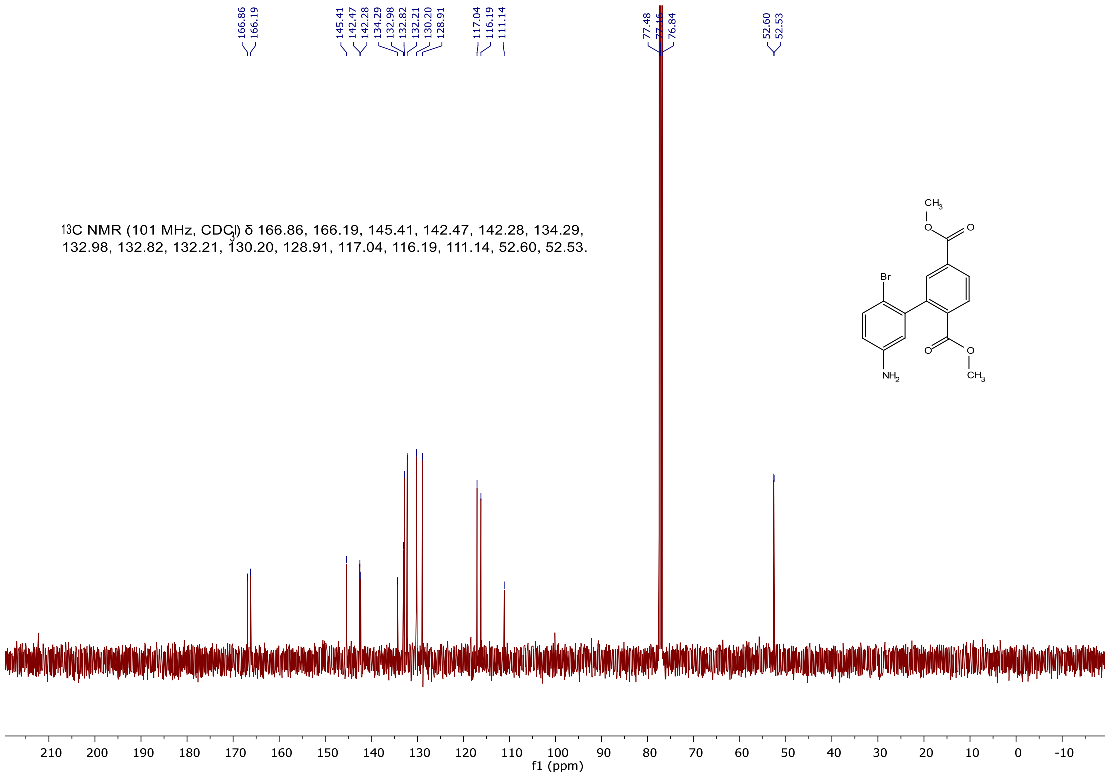

Mass Spectrum SmartFormula Report

Analysis Info

Analysis Name	N:\new acq data\RM098 001.d	Acquisition Date	27.07.2015 17:55:44
Method	hn Direct_Infusion_pos mode_75-1700 mid 4eV.m	Operator	hn
Sample Name	Rajesh Mannancherry, RM098	Instrument / Ser#	maXis 4G 21243
Comment	ca. 6 ug/ml ACN hinter ACN/HCOOH		

Acquisition Parameter

Source Type	ESI	Ion Polarity	Positive	Set Nebulizer	0.4 Bar
Focus	Not active	Set Capillary	3600 V	Set Dry Heater	180 °C
Scan Begin	75 m/z	Set End Plate Offset	-500 V	Set Dry Gas	4.0 l/min
Scan End	1700 m/z	Set Collision Cell RF	500.0 Vpp	Set Ion Energy (MS only)	4.0 eV

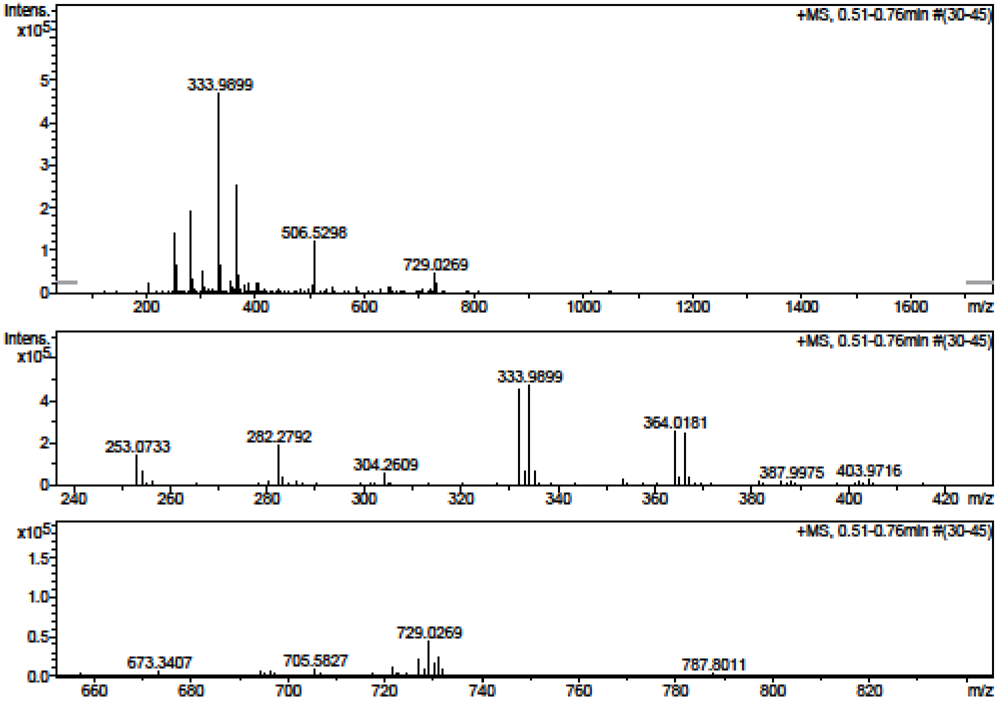

Meas. m/z	#	Formula	Score	m/z	err [mDa]	err [ppm]	mSigma	rdb	e⁻ Conf	N-Rule	z
331.9919	1	C 15 H 11 Br N O 3	100.00	331.9917	-0.2	-0.7	26.1	10.5	even	ok	1+
364.0181	1	C 16 H 15 Br N O 4	100.00	364.0179	-0.2	-0.5	16.3	9.5	even	ok	
385.9996	1	C 16 H 14 Br N Na O 4	100.00	385.9998	0.2	0.6	11.2	9.5	even	ok	
401.9736	1	C 16 H 14 Br K N O 4	100.00	401.9738	0.2	0.5	9.3	9.5	even	ok	
727.0286	1	C 32 H 29 Br 2 N 2 O 8	100.00	727.0285	-0.1	-0.1	9.0	18.5	even	ok	

HR-ESI spectra of compound 15

Mass Spectrum SmartFormula Report

Analysis Info

Analysis Name	E:\acq data for data analysis\RM135_2 001.d	Acquisition Date	31.10.2017 12:17:41
Method	21 Direct_pos_low.m	Operator	hn
Sample Name	Rajesh Mannancherry	Instrument / Ser#	maXis 4G 21243
Comment	RM135 2. Probe, ca. 10 ug/ml MeOH		

Acquisition Parameter

Source Type	ESI	Ion Polarity	Positive	Set Nebulizer	0.4 Bar
Focus	Not active	Set Capillary	3600 V	Set Dry Heater	180 °C
Scan Begin	75 m/z	Set End Plate Offset	-500 V	Set Dry Gas	3.0 l/min
Scan End	1700 m/z	Collision Energy	8.0 eV	Set Ion Energy (MS only)	4.0 eV

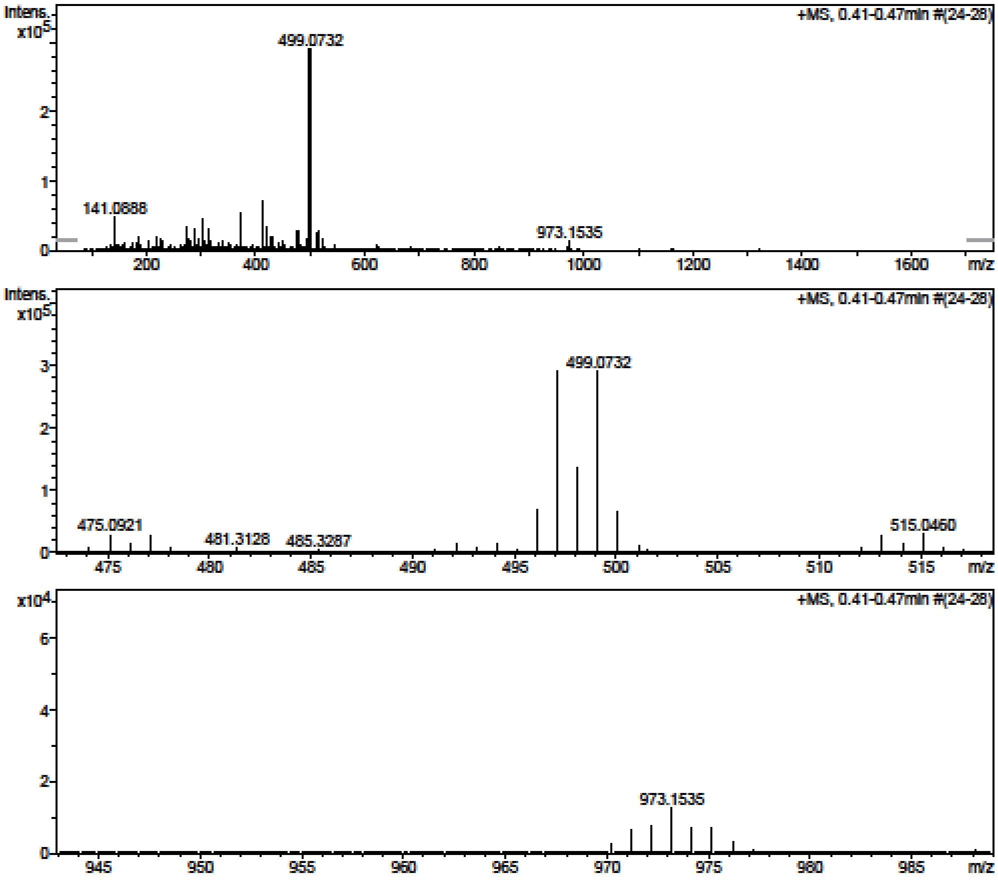

Meas. m/z	#	Formula	Score	m/z	err [mDa]	err [ppm]	mSigma	rdb	e¯ Conf	z
475.0921	1	C 22 H 25 B Br O 6	100.00	475.0926	0.1	0.3	26.2	10.5	even	1+
492.1186	1	C 22 H 28 B Br N O 6	100.00	492.1191	0.2	0.3	32.6	9.5	even	
497.0750	1	C 22 H 24 B Br Na O 6	100.00	497.0745	-0.4	-0.8	6.9	10.5	even	
513.0479	1	C 22 H 24 B Br K O 6	100.00	513.0485	0.2	0.5	8.3	10.5	even	
971.1558	1	C 44 H 48 B 2 Br 2 Na O 12	100.00	971.1593	3.3	3.4	15.9	20.5	even	

^{1}H-, ^{13}C-NMR (CD_3CN, 400/101 MHz, 25 °C) and HR-ESI spectra of compound 11

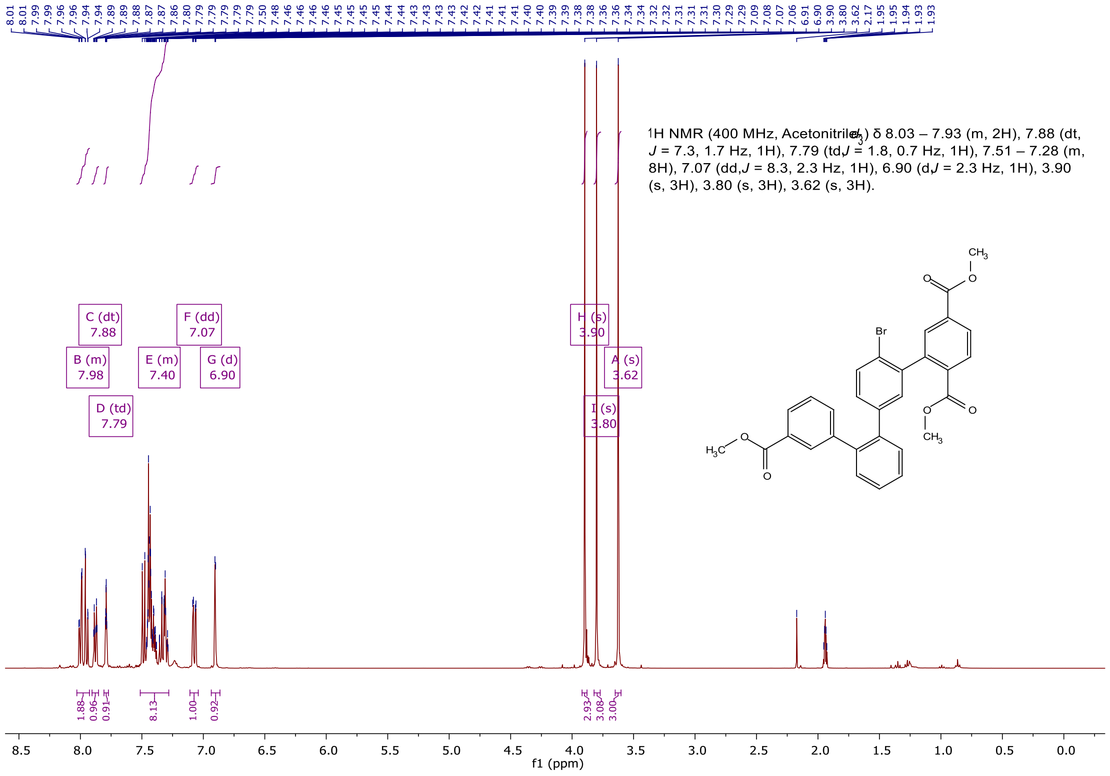

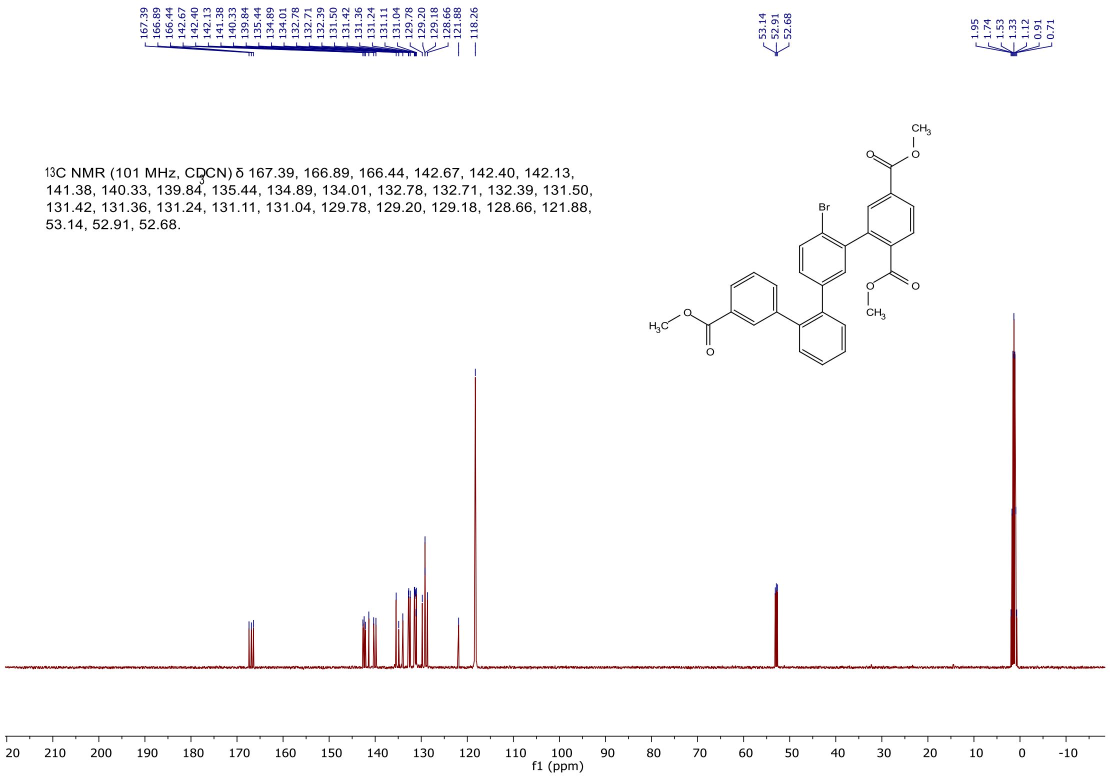

D:\Data\Service\Data\juQEx5486
Client: Juricek

18.09.2017 16:17:42
(+)-HR-ESI-MS

Sample: RM144
Solvent: MeOH/CHCl3 3:2

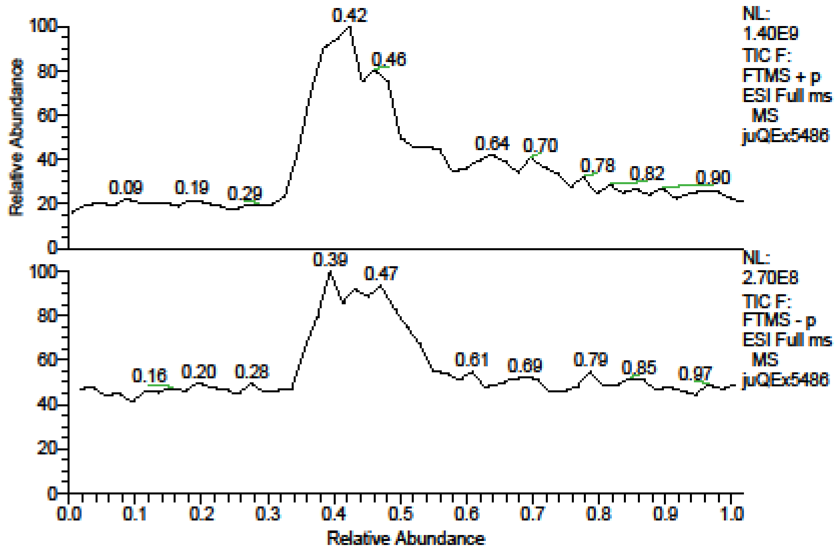

juQEx5486#41-45 RT: 0.40-0.44 AV: 3
SB: 26 0.07-0.29 , 0.71-0.98
T: FTMS + p ESI Full lock ms [100.00-1500.00]
m/z= 558.74752-584.15919

m/z	Intensity	Relative	Theo. Mass	Delta (ppm)	RDB equiv.	Composition
559.07543	75022832.0	100.00	559.07535	0.13	20.0	$C_{31} H_{23} O_3 N Br Na$
			559.07508	0.62	18.5	$C_{30} H_{24} O_6 Br$
561.07329	73179624.0	97.54	561.07228	1.80	27.0	$C_{36} H_{20} O N Br$
576.10187	59435140.0	79.22	576.10190	-0.06	19.0	$C_{31} H_{26} O_3 N_2 Br Na$
			576.10163	0.42	17.5	$C_{30} H_{27} O_6 N Br$
578.09959	60059192.0	80.05	578.09883	1.31	26.0	$C_{36} H_{23} O N_2 Br$
581.05630	34302804.0	45.72	581.05702	-1.25	18.5	$C_{30} H_{23} O_6 Br Na$
			581.05540	1.53	17.5	$C_{27} H_{22} O_8 N_2 Br$
583.05478	35065152.0	46.74	583.05422	0.96	27.0	$C_{36} H_{19} O N Br Na$
			583.05395	1.43	25.5	$C_{36} H_{20} O_4 Br$

juQEx5486 #41-45 RT: 0.40-0.44 AV: 3 SB: 26 0.07-0.29 , 0.71-0.98 NL: 1.15E8
T: FTMS + p ESI Full lock ms [100.00-1500.00]

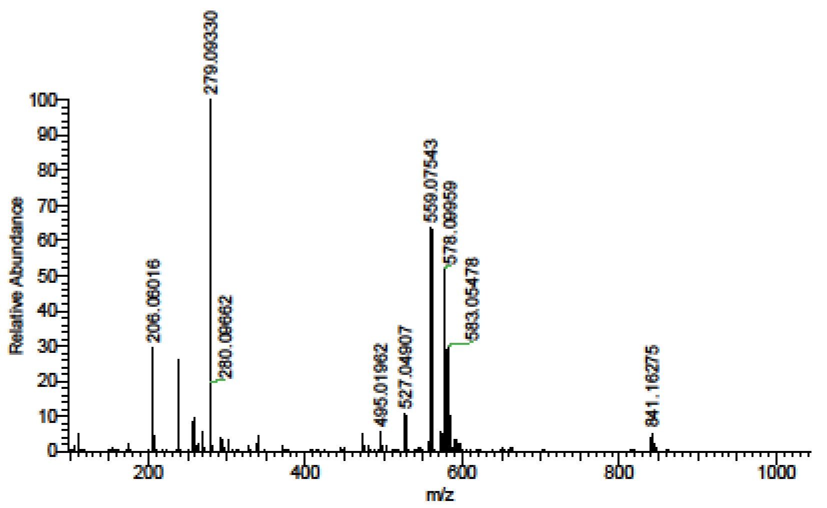

juQEx5486 #41-45 RT: 0.40-0.44 AV: 3 SB: 26 0.07-0.29 , 0.71-0.98 NL: 7.34E7
T: FTMS + p ESI Full lock ms [100.00-1500.00]

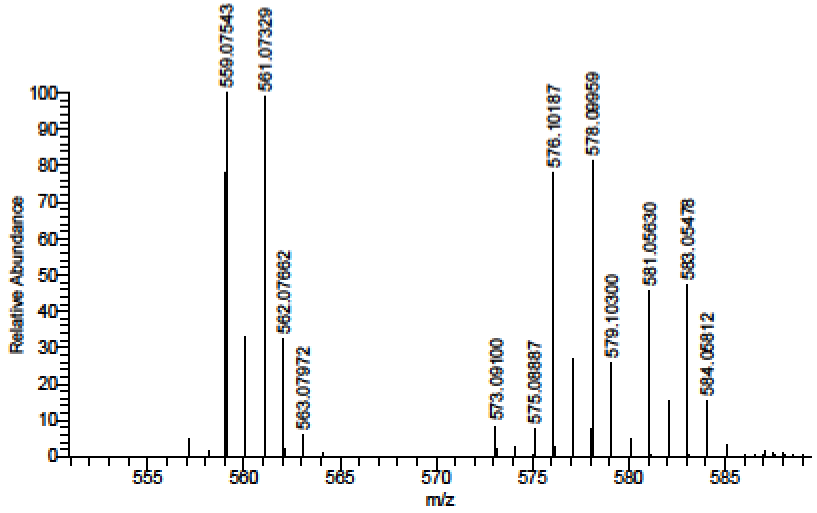

^{1}H-, ^{13}C-NMR (CD_3CN, 400/101 MHz, 25 °C) and HR-ESI spectra of compound 12

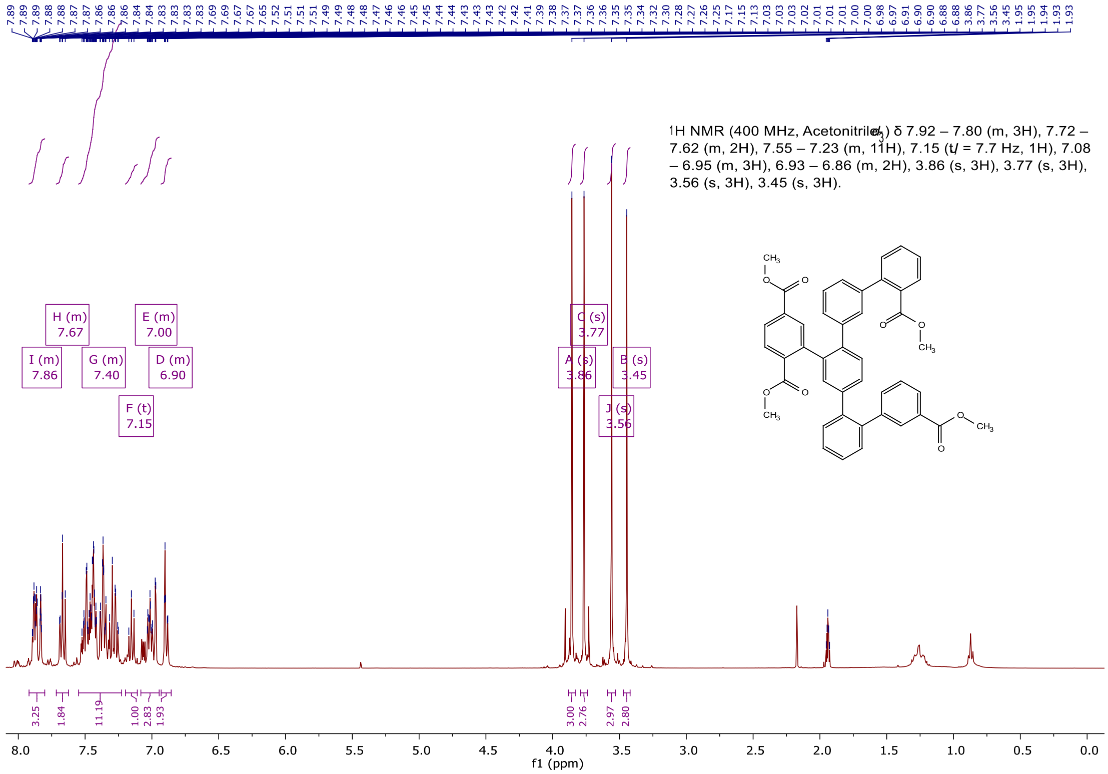

^{1}H NMR (400 MHz, Acetonitrile-d_3) δ 7.92 – 7.80 (m, 3H), 7.72 – 7.62 (m, 2H), 7.55 – 7.23 (m, 11H), 7.15 (t, J = 7.7 Hz, 1H), 7.08 – 6.95 (m, 3H), 6.93 – 6.86 (m, 2H), 3.86 (s, 3H), 3.77 (s, 3H), 3.56 (s, 3H), 3.45 (s, 3H).

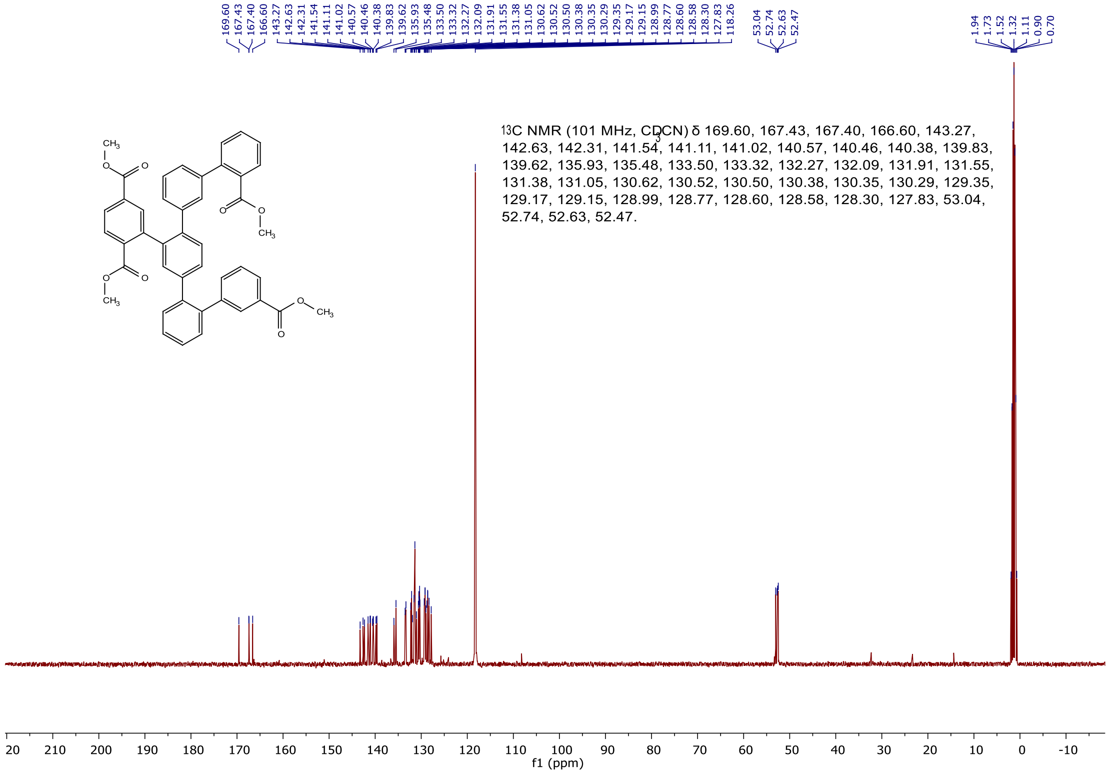

^{13}C NMR (101 MHz, CD_3CN) δ 169.60, 167.43, 167.40, 166.60, 143.27, 142.63, 142.31, 141.54, 141.11, 141.02, 140.57, 140.46, 140.38, 139.83, 139.62, 135.93, 135.48, 133.50, 133.32, 132.27, 132.09, 131.91, 131.55, 131.38, 131.05, 130.62, 130.52, 130.50, 130.38, 130.35, 130.29, 129.35, 129.17, 129.15, 128.99, 128.77, 128.60, 128.58, 128.30, 127.83, 53.04, 52.74, 52.63, 52.47.

Mass Spectrum SmartFormula Report

Analysis Info

Analysis Name	N:\new acq data\RM138 001.d	Acquisition Date	20.11.2015 09:20:09
Method	hn Direct_Infusion_pos mode_75-1700 mid 4eV.m	Operator	hn
Sample Name	Rajesh Mannancherry	Instrument / Ser#	maXis 4G 21243
Comment	RM138, ca. 8 ug/ml MeCN		

Acquisition Parameter

Source Type	ESI	Ion Polarity	Positive	Set Nebulizer	0.4 Bar
Focus	Not active	Set Capillary	3600 V	Set Dry Heater	180 °C
Scan Begin	75 m/z	Set End Plate Offset	-500 V	Set Dry Gas	4.0 l/min
Scan End	1700 m/z	Set Collision Cell RF	350.0 Vpp	Set Ion Energy (MS only)	4.0 eV

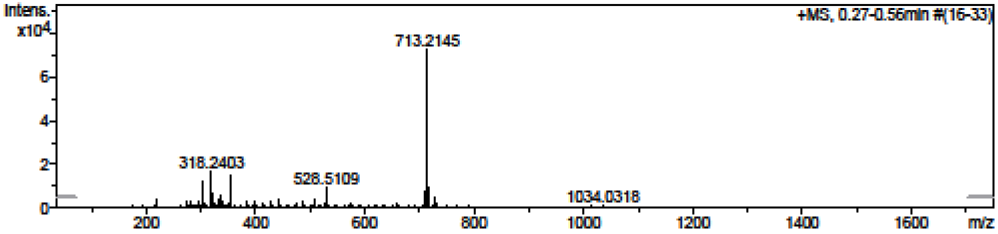

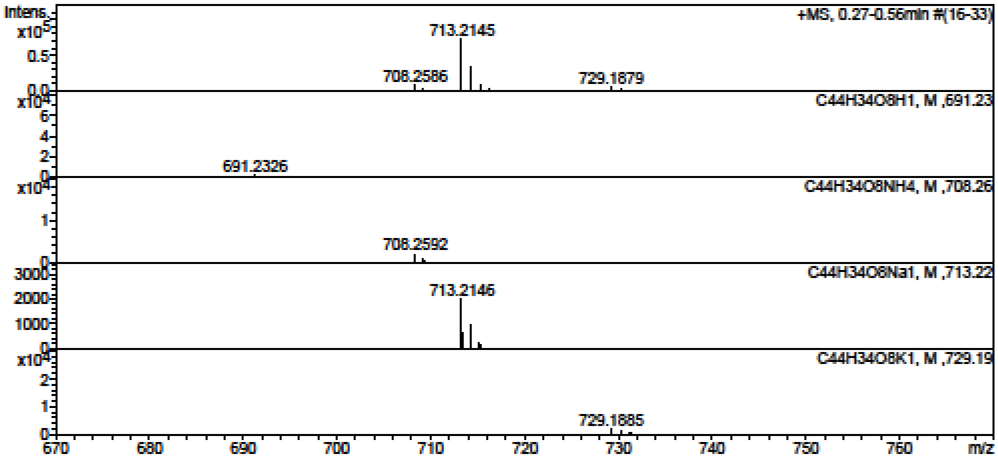

Meas. m/z	#	Formula	Score	m/z	err [mDa]	err [ppm]	mSigma	rdb	e⁻ Conf	N-Rule	z
691.2321	1	C 44 H 35 O 8	100.00	691.2326	0.5	0.8	19.7	27.5	even	ok	1+
708.2586	1	C 44 H 38 N O 8	100.00	708.2592	0.6	0.9	5.5	26.5	even	ok	
713.2145	1	C 44 H 34 Na O 8	100.00	713.2146	0.1	0.1	6.3	27.5	even	ok	
729.1879	1	C 44 H 34 K O 8	100.00	729.1885	0.6	0.8	13.3	27.5	even	ok	

^{1}H-, ^{13}C-NMR (CD_3CN, 400/101 MHz, 25 °C) and HR-ESI spectra of compound 13

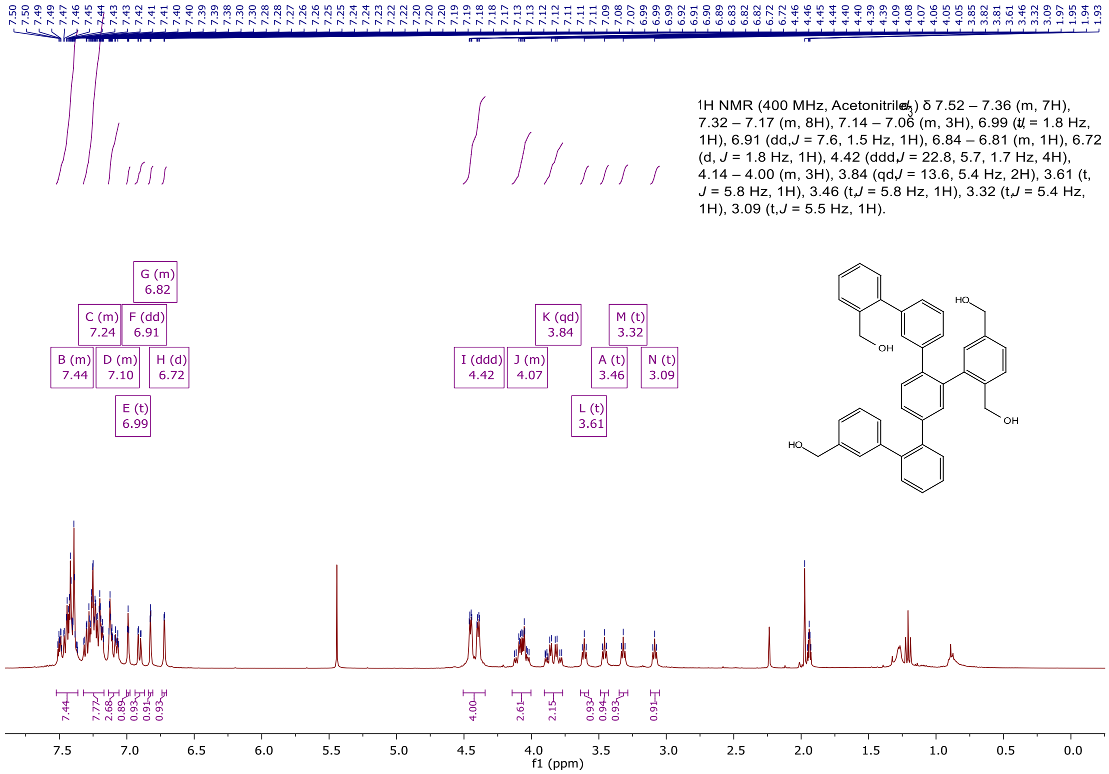

^{1}H NMR (400 MHz, Acetonitrile-d_3) δ 7.52 – 7.36 (m, 7H), 7.32 – 7.17 (m, 8H), 7.14 – 7.06 (m, 3H), 6.99 (t, J = 1.8 Hz, 1H), 6.91 (dd, J = 7.6, 1.5 Hz, 1H), 6.84 – 6.81 (m, 1H), 6.72 (d, J = 1.8 Hz, 1H), 4.42 (ddd, J = 22.8, 5.7, 1.7 Hz, 4H), 4.14 – 4.00 (m, 3H), 3.84 (qd, J = 13.6, 5.4 Hz, 2H), 3.61 (t, J = 5.8 Hz, 1H), 3.46 (t, J = 5.8 Hz, 1H), 3.32 (t, J = 5.4 Hz, 1H), 3.09 (t, J = 5.5 Hz, 1H).

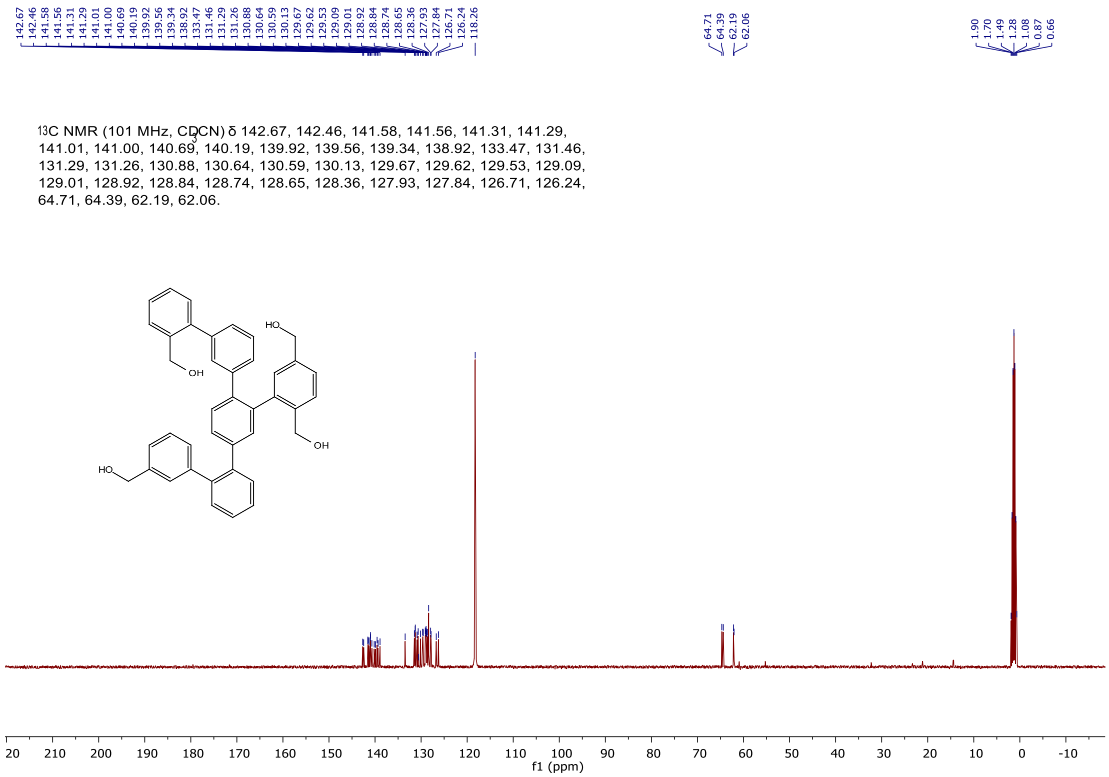

^{13}C NMR (101 MHz, CD_3CN) δ 142.67, 142.46, 141.58, 141.56, 141.31, 141.29, 141.01, 141.00, 140.69, 140.19, 139.92, 139.56, 139.34, 138.92, 133.47, 131.46, 131.29, 131.26, 130.88, 130.64, 130.59, 130.13, 129.67, 129.62, 129.53, 129.09, 129.01, 128.92, 128.84, 128.74, 128.65, 128.36, 127.93, 127.84, 126.71, 126.24, 64.71, 64.39, 62.19, 62.06.

Mass Spectrum SmartFormula Report

Analysis Info

Analysis Name	N:\new acq data\RM127 002.d	Acquisition Date	11.11.2015 17:55:18
Method	140815 mml putz quad tune_mid_pos.m	Operator	hn
Sample Name	Rajesh Mannancherry	Instrument / Ser#	maXis 4G 21243
Comment	RM127, ca. 10 ug/mL MeCN		

Acquisition Parameter

Source Type	ESI	Ion Polarity	Positive	Set Nebulizer	0.4 Bar
Focus	Active	Set Capillary	3800 V	Set Dry Heater	180 °C
Scan Begin	100 m/z	Set End Plate Offset	-500 V	Set Dry Gas	4.0 l/min
Scan End	2900 m/z	Set Collision Cell RF	1500.0 Vpp	Set Ion Energy (MS only)	10.0 eV

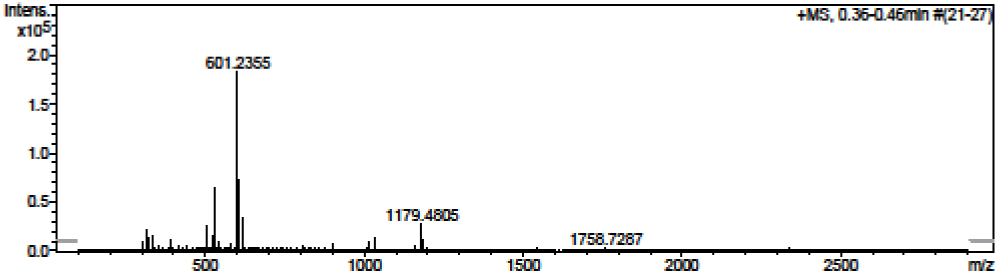

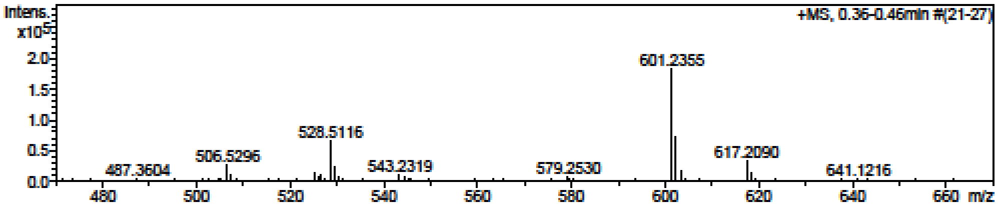

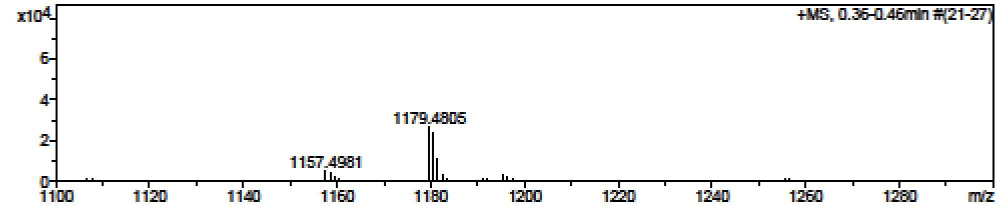

Meas. m/z	#	Formula	Score	m/z	err [mDa]	err [ppm]	mSigma	rdb	e⁻ Conf	N-Rule	z
579.2530	1	C 40 H 35 O 4	100.00	579.2530	-0.0	-0.0	3.0	23.5	even	ok	1+
601.2355	1	C 40 H 34 Na O 4	100.00	601.2349	-0.6	-1.0	19.4	23.5	even	ok	
617.2090	1	C 40 H 34 K O 4	100.00	617.2089	-0.1	-0.2	15.7	23.5	even	ok	
1157.4981	1	C 80 H 69 O 8	100.00	1157.4987	0.6	0.5	15.4	46.5	even	ok	
1179.4805	1	C 80 H 68 Na O 8	100.00	1179.4806	0.2	0.1	2.8	46.5	even	ok	
1195.4543	1	C 80 H 68 K O 8	100.00	1195.4546	0.3	0.2	13.3	46.5	even	ok	
1735.7429	1	C 120 H 103 O 12	100.00	1735.7444	1.5	0.9	93.7	69.5	even	ok	
1757.7252	1	C 120 H 102 Na O 12	100.00	1757.7263	1.2	0.7	20.9	69.5	even	ok	
1773.6992	1	C 120 H 102 K O 12	100.00	1773.7003	1.1	0.6	27.6	69.5	even	ok	

^{1}H- and ^{13}C-NMR (Toluene-d_8, 600/125 MHz, 25 °C) and HR-ESI spectra of compound 1

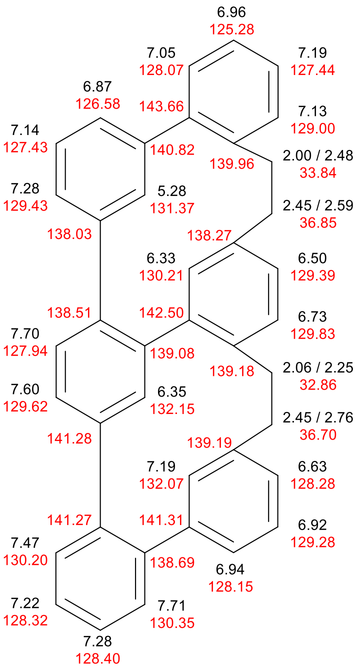

1 in toluene-d_8 ^{1}H-NMR

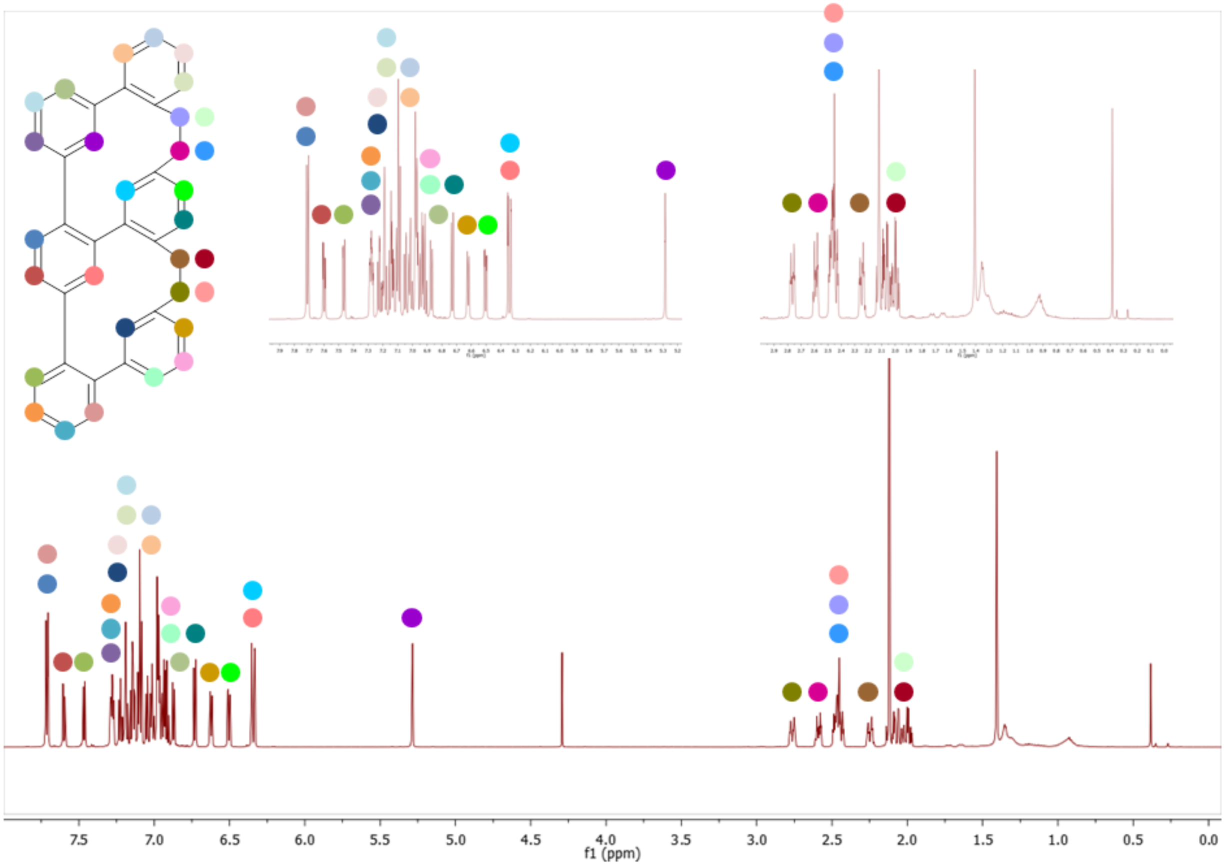

1 in toluene-d$_8$ ^{13}C-NMR

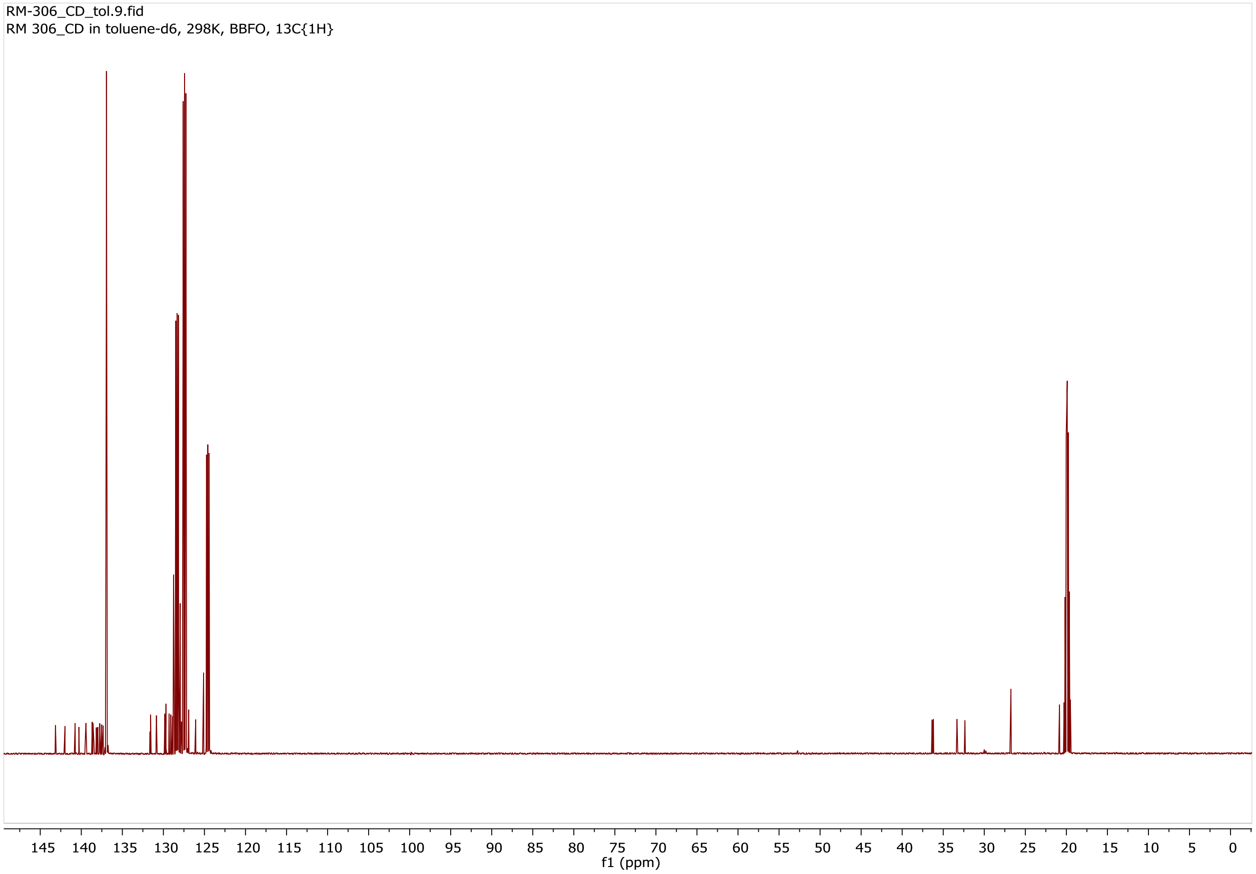

HR-EI-Report (Thermo DFS)

Sample: Client:

2953juhr-cmass3 #9-18 RT: 1.00-2.13 AV: 10 NL: 5.76E4
T: + c EI Full ms [489.50-533.50]

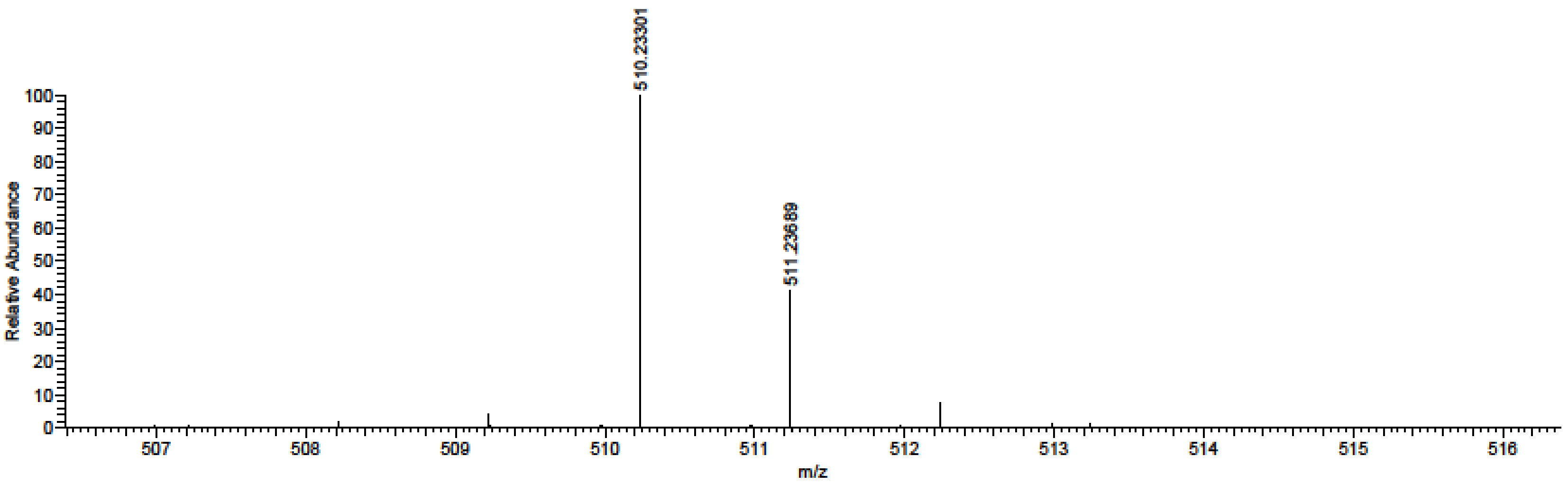

2953juhr-cmass3#9-18 RT: 1.00-2.13 AV: 10
T: + c EI Full ms [489.50-533.50]
m/z= 508.92613-510.44889

m/z	Intensity	Relative	Theo. Mass	Delta (ppm)	Composition
510.23301	57574.6	100.00	510.23420	-2.34	$C_{40}H_{30}$

^{1}H-NMR (Toluene-d8, 600/125 MHz, 25 °C) and HR-ESI spectra of compound 2

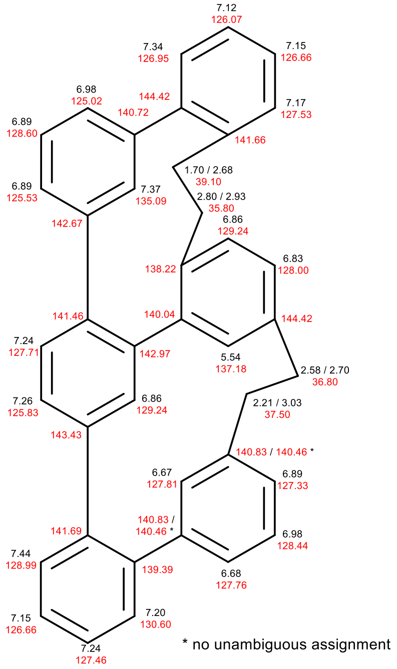

2 in toluene-d8 ^{1}H-NMR

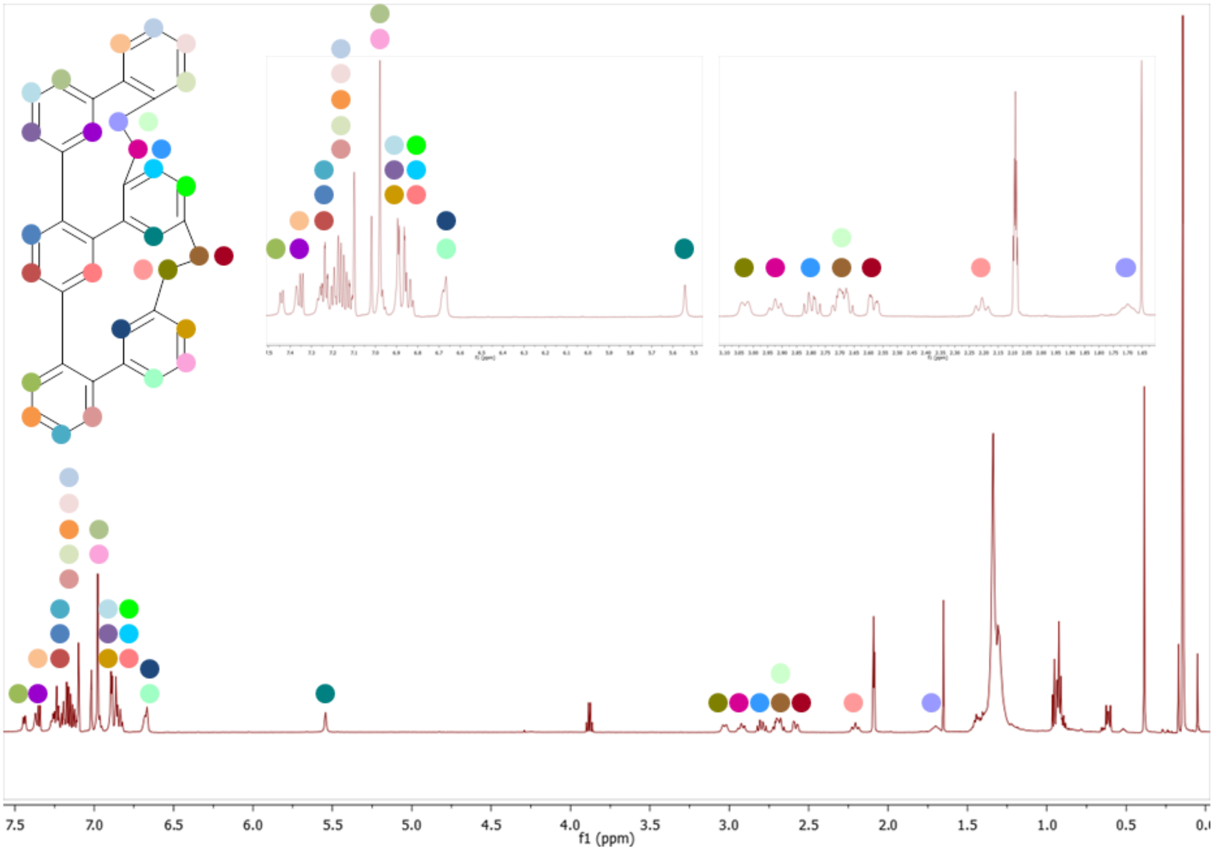

HR-EI-Report (Thermo DFS)

Sample: Client:

2952juhr-cmass1 #3-14 RT: 0.25-1.63 AV: 12 NL: 9.22E4
T: + c EI Full ms [489.50-533.50]

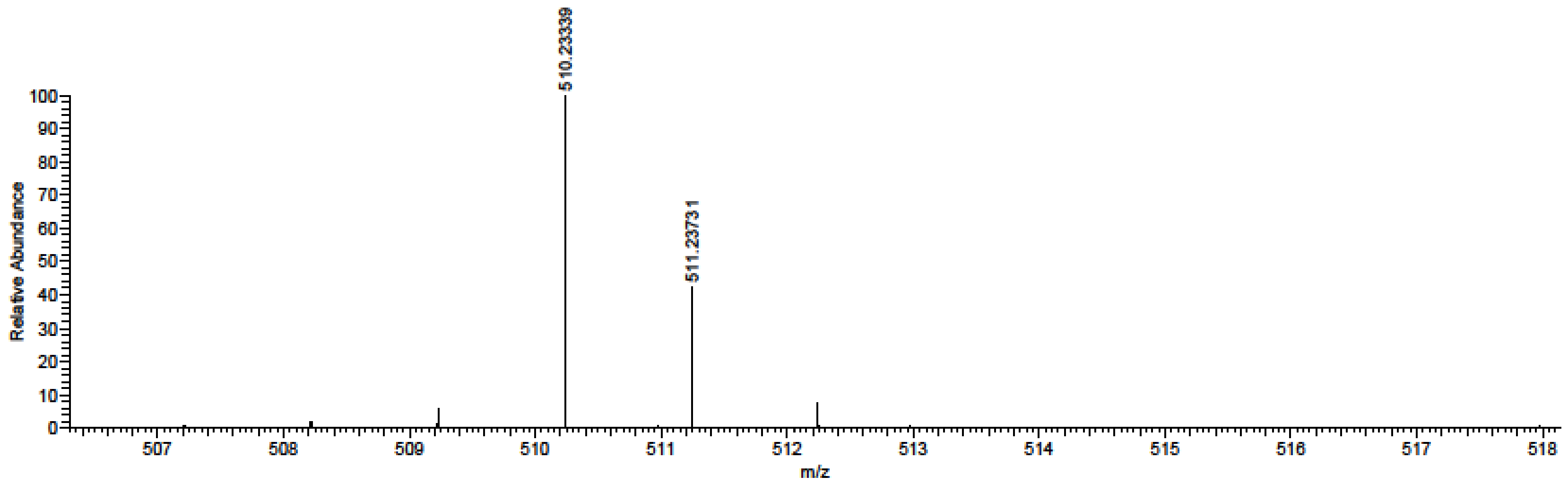

2952juhr-cmass1#3-14 RT: 0.25-1.63 AV: 12
T: + c EI Full ms [489.50-533.50]
m/z= 507.90218-513.41689

m/z	Intensity	Relative	Theo. Mass	Delta (ppm)	Composition
509.22585	5166.7	5.60	509.22638	-1.04	$C_{40}H_{29}$
510.23339	92199.1	100.00	510.23420	-1.59	$C_{40}H_{30}$

UV-Vis spectroscopy

The UV-Vis spectra were recorded on a *Shimadzu* UV spectrometer UV-1800. The wavelength was measured in nm. All solutions were prepared and measured under air saturated conditions in *n*-hexane:*i*-PrOH 99:1. UV/Vis spectra were recorded on a Shimadzu UV spectrometer UV-1800 using optical 1115F-QS Hellma cuvettes (10 mm light path).

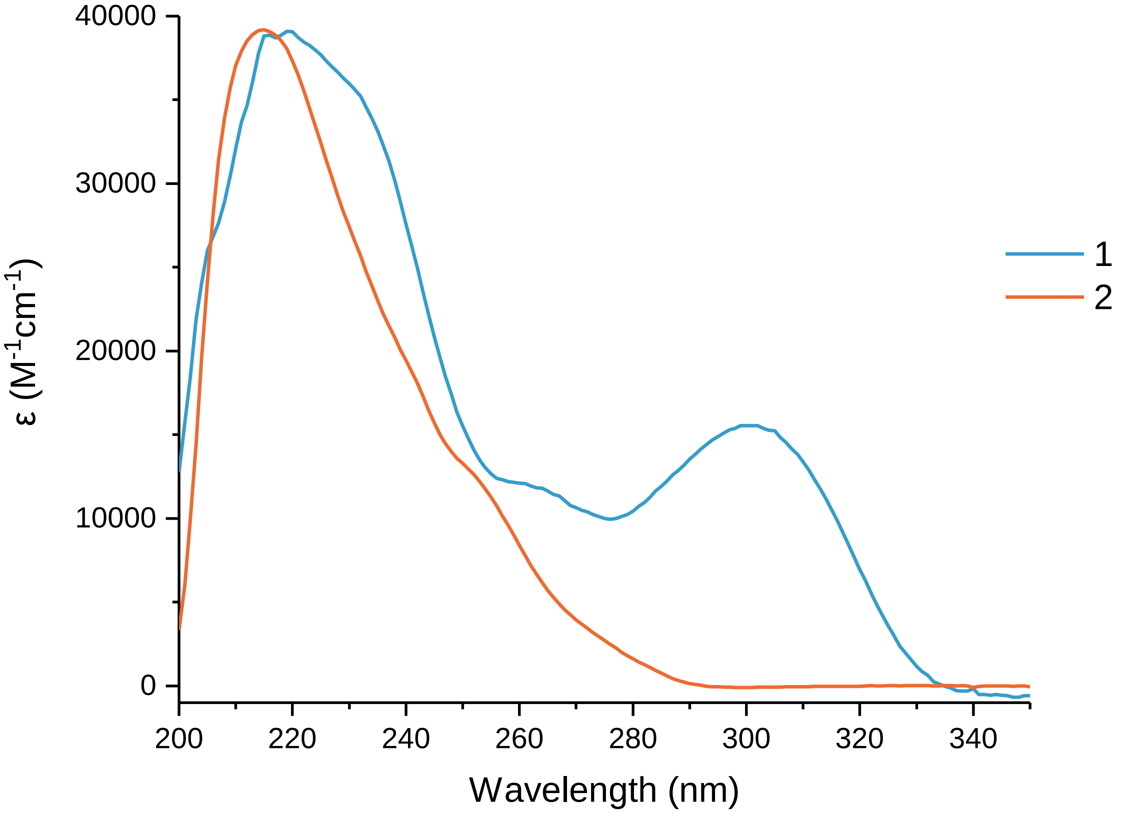

Figure S4: UV-Vis spectra of the racemate **1** and **2**.

Experimental UV-Vis and CD: Data of 1

Table S1: Δε and ε values of compound **1**.

λ [nm]	Δε for enantiomer **1a** [$M^{-1}cm^{-1}$]	Δε for enantiomer **1b** [$M^{-1}cm^{-1}$]	ε for racemate [$M^{-1}cm^{-1}$]
350	-4.16E-01	-3.75E-01	-589.73658
349	-4.28E-01	-5.08E-01	-589.73658
348	-3.72E-01	-4.59E-01	-669.43071
347	-3.99E-01	-3.67E-01	-669.43071
346	-3.94E-01	-4.45E-01	-589.73658
345	-3.69E-01	-5.11E-01	-557.85893
344	-4.43E-01	-5.20E-01	-504.7295
343	-3.56E-01	-5.29E-01	-557.85893
342	-3.59E-01	-6.26E-01	-504.7295
341	-3.32E-01	-6.45E-01	-504.7295
340	-2.83E-01	-5.95E-01	-175.32709
339	-2.29E-01	-7.18E-01	-308.15065
338	-1.36E-01	-7.89E-01	-308.15065
337	-6.00E-02	-8.85E-01	-276.27299
336	1.05E-02	-1.01E+00	-111.57179
335	1.21E-01	-1.14E+00	-26.564711
334	3.45E-01	-1.20E+00	116.884727
333	4.72E-01	-1.37E+00	255.021223
332	5.59E-01	-1.55E+00	616.30129
331	8.18E-01	-1.76E+00	844.757803
330	9.12E-01	-2.05E+00	1158.22139
329	1.27E+00	-2.30E+00	1572.63088

λ [nm]	Δε for enantiomer **1a** [$M^{-1}cm^{-1}$]	Δε for enantiomer **1b** [$M^{-1}cm^{-1}$]	ε for racemate [$M^{-1}cm^{-1}$]
328	1.54E+00	-2.53E+00	1997.66625
327	1.74E+00	-2.89E+00	2396.13691
326	1.96E+00	-3.26E+00	3007.12526
325	2.44E+00	-3.47E+00	3570.29713
324	2.58E+00	-3.87E+00	4191.91136
323	3.02E+00	-4.12E+00	4840.0903
322	3.23E+00	-4.52E+00	5514.83396
321	3.38E+00	-4.89E+00	6274.58469
320	3.79E+00	-5.19E+00	6949.32834
319	4.09E+00	-5.42E+00	7677.20142
318	4.29E+00	-5.75E+00	8436.95214
317	4.55E+00	-5.93E+00	9143.57345
316	4.78E+00	-6.26E+00	9871.44653
315	5.01E+00	-6.34E+00	10546.1902
314	5.25E+00	-6.63E+00	11189.0562
313	5.38E+00	-6.74E+00	11784.1057
312	5.51E+00	-6.88E+00	12347.2776
311	5.52E+00	-7.05E+00	12905.1365
310	5.69E+00	-7.09E+00	13383.3013
309	5.93E+00	-7.11E+00	13829.5884
308	5.93E+00	-7.21E+00	14143.052
307	5.90E+00	-7.29E+00	14536.2097
306	5.93E+00	-7.30E+00	14817.7957
305	5.95E+00	-7.30E+00	15232.2052

λ [nm]	Δε for enantiomer **1a** [$M^{-1}cm^{-1}$]	Δε for enantiomer **1b** [$M^{-1}cm^{-1}$]	ε for racemate [$M^{-1}cm^{-1}$]
304	5.98E+00	-7.27E+00	15264.0828
303	5.87E+00	-7.19E+00	15380.9675
302	5.86E+00	-7.08E+00	15545.6687
301	5.85E+00	-7.10E+00	15545.6687
300	5.70E+00	-7.13E+00	15545.6687
299	5.66E+00	-7.03E+00	15545.6687
298	5.42E+00	-7.01E+00	15380.9675
297	5.44E+00	-6.74E+00	15295.9605
296	5.41E+00	-6.69E+00	15099.3816
295	5.17E+00	-6.49E+00	14902.8027
294	4.98E+00	-6.40E+00	14674.3462
293	4.91E+00	-6.12E+00	14424.638
292	4.76E+00	-6.02E+00	14143.052
291	4.51E+00	-5.82E+00	13829.5884
290	4.19E+00	-5.60E+00	13548.0025
289	4.08E+00	-5.26E+00	13186.7224
288	3.81E+00	-5.01E+00	12873.2588
287	3.36E+00	-4.58E+00	12591.6729
286	3.02E+00	-4.09E+00	12230.3928
285	2.51E+00	-3.76E+00	11916.9293
284	1.93E+00	-3.26E+00	11635.3433
283	1.59E+00	-2.72E+00	11242.1856
282	1.09E+00	-2.35E+00	10960.5997
281	7.14E-01	-1.93E+00	10710.8914
280	4.73E-01	-1.38E+00	10429.3055

λ [nm]	Δε for enantiomer **1a** [$M^{-1}cm^{-1}$]	Δε for enantiomer **1b** [$M^{-1}cm^{-1}$]	ε for racemate [$M^{-1}cm^{-1}$]
279	-1.18E-01	-1.04E+00	10232.7266
278	-5.43E-01	-4.98E-01	10115.8419
277	-9.12E-01	1.40E-02	10004.2701
276	-1.71E+00	5.42E-01	9951.14066
275	-2.27E+00	1.15E+00	10004.2701
274	-2.79E+00	1.64E+00	10115.8419
273	-3.23E+00	2.21E+00	10232.7266
272	-3.69E+00	2.59E+00	10397.4278
271	-4.11E+00	3.19E+00	10482.4349
270	-4.70E+00	3.69E+00	10647.1361
269	-5.36E+00	4.35E+00	10764.0208
268	-5.89E+00	4.89E+00	11072.1715
267	-6.36E+00	5.30E+00	11353.7574
266	-6.61E+00	5.52E+00	11438.7645
265	-6.91E+00	5.85E+00	11635.3433
264	-7.20E+00	6.21E+00	11800.0445
263	-7.58E+00	6.43E+00	11831.9222
262	-7.87E+00	6.78E+00	11948.8069
261	-8.14E+00	6.92E+00	12081.6305
260	-8.28E+00	7.12E+00	12113.5081
259	-8.53E+00	7.29E+00	12145.3858
258	-8.64E+00	7.56E+00	12198.5152
257	-8.96E+00	7.84E+00	12310.087
256	-9.33E+00	8.24E+00	12395.094
255	-1.01E+01	8.71E+00	12676.68

λ [nm]	Δε for enantiomer **1a** [$M^{-1}cm^{-1}$]	Δε for enantiomer **1b** [$M^{-1}cm^{-1}$]	ε for racemate [$M^{-1}cm^{-1}$]
254	-1.07E+01	9.44E+00	13037.9601
253	-1.18E+01	1.05E+01	13484.2472
252	-1.29E+01	1.16E+01	14079.2967
251	-1.44E+01	1.29E+01	14754.0404
250	-1.59E+01	1.43E+01	15513.7911
249	-1.74E+01	1.55E+01	16353.236
248	-1.83E+01	1.61E+01	17394.5726
247	-1.84E+01	1.62E+01	18435.9093
246	-1.78E+01	1.54E+01	19588.8177
245	-1.66E+01	1.39E+01	20826.7333
244	-1.46E+01	1.17E+01	22176.2206
243	-1.23E+01	9.33E+00	23499.1432
242	-9.81E+00	6.79E+00	24986.767
241	-7.55E+00	4.48E+00	26336.2543
240	-5.35E+00	2.27E+00	27574.1698
239	-3.59E+00	5.52E-01	28944.9089
238	-2.17E+00	-8.28E-01	30182.8244
237	-1.06E+00	-1.86E+00	31335.7328
236	-2.29E-01	-2.80E+00	32292.0624
235	5.14E-01	-3.31E+00	33136.8202
234	9.95E-01	-3.72E+00	33896.571
233	1.49E+00	-4.31E+00	34571.3146
232	1.77E+00	-4.77E+00	35214.1806
231	2.38E+00	-5.22E+00	35612.6513
230	3.03E+00	-6.05E+00	35973.9313

λ [nm]	Δε for enantiomer **1a** [$M^{-1}cm^{-1}$]	Δε for enantiomer **1b** [$M^{-1}cm^{-1}$]	ε for racemate [$M^{-1}cm^{-1}$]
229	3.77E+00	-6.40E+00	36287.3949
228	4.22E+00	-6.97E+00	36648.675
227	4.48E+00	-7.14E+00	36962.1386
226	4.83E+00	-7.29E+00	37296.8539
225	5.03E+00	-7.46E+00	37690.0117
224	4.95E+00	-7.43E+00	37971.5976
223	4.72E+00	-7.28E+00	38253.1835
222	4.08E+00	-7.12E+00	38449.7624
221	3.49E+00	-6.49E+00	38731.3483
220	2.50E+00	-5.64E+00	39060.7507
219	1.47E+00	-4.70E+00	39092.6284
218	1.27E-01	-3.42E+00	38864.1719
217	-1.32E+00	-2.39E+00	38694.1577
216	-2.87E+00	-1.44E+00	38858.8589
215	-4.32E+00	9.99E-03	38805.7295
214	-5.49E+00	8.26E-01	37806.8964
213	-6.61E+00	1.43E+00	36133.3196
212	-6.97E+00	1.80E+00	34629.757
211	-7.08E+00	1.86E+00	33699.9921
210	-7.12E+00	1.54E+00	32084.8577
209	-6.50E+00	1.19E+00	30459.0974
208	-5.64E+00	6.28E-01	28881.1536
207	-4.77E+00	-2.47E-02	27664.4898
206	-3.99E+00	-5.45E-01	26840.9838
205	-3.18E+00	-1.18E+00	25980.2871

λ<br>[nm]	Δε for enantiomer **1a**<br>[$M^{-1}cm^{-1}$]	Δε for enantiomer **1b**<br>[$M^{-1}cm^{-1}$]	ε for racemate<br>[$M^{-1}cm^{-1}$]
204	-2.78E+00	-1.61E+00	24009.1856
203	-2.14E+00	-1.78E+00	21910.5735
202	-1.82E+00	-1.97E+00	18430.5963
201	-1.73E+00	-1.52E+00	15726.3088
200	-1.18E+00	-1.41E+00	12751.0612
199	-1.20E+00	-1.19E+00	
198	-1.03E+00	-9.22E-01	
197	-7.70E-01	-7.11E-01	
196	-9.11E-01	-5.67E-01	
195	-9.60E-01	-7.57E-01	
194	-4.81E-01	-3.36E-01	
193	-6.57E-01	-3.55E-01	
192	-4.75E-01	-3.47E-01	
191	-4.87E-01	-3.77E-01	
190	-3.02E-01	-1.56E-01	

Experimental UV-Vis and CD: Data of 2

Table S2: $\Delta\varepsilon$ and ε values of compound **2**.

λ [nm]	$\Delta\varepsilon$ for enantiomer **2a** [$M^{-1}cm^{-1}$]	$\Delta\varepsilon$ for enantiomer **2b** [$M^{-1}cm^{-1}$]	ε for racemate [$M^{-1}cm^{-1}$]
350	0.077157946	1.084618898	-39.99907
349	0.047921245	0.895027368	-2.73928
348	0.155218993	1.23537446	-2.73928
347	0.045038046	0.928869046	-23.36773
346	-0.052453778	0.828477684	-2.73928
345	0.131566724	0.931113413	-2.73928
344	0.040685192	0.867987729	-2.73928
343	0.129052385	0.90306266	-2.73928
342	-0.003990543	1.000757718	4.3045824
341	0.061330158	0.960634877	-29.43328
340	-0.01932384	1.022971585	-84.30273
339	0.049853835	0.9264868	4.3045824
338	0.065814735	1.032485249	13.10941
337	0.052482911	0.892093605	4.3045824
336	0.05441819	0.775830818	30.719065
335	0.114460702	1.075358013	20.153272
334	0.063681805	1.019065009	4.3045824
333	0.023536501	0.978114892	4.3045824
332	0.081929364	1.082244312	13.10941
331	0.027389581	1.002389288	13.10941
330	0.051984525	0.976077345	13.10941
329	0.083596179	1.134132843	20.935923

λ [nm]	Δε for enantiomer **2a** [$M^{-1}cm^{-1}$]	Δε for enantiomer **2b** [$M^{-1}cm^{-1}$]	ε for racemate [$M^{-1}cm^{-1}$]
328	0.080243278	1.046771063	13.10941
327	0.08349444	1.06587499	4.3045824
326	0.108023814	1.209039537	13.10941
325	0.074820641	1.167928562	12.131096
324	0.130240982	1.214309585	4.3045824
323	0.13889506	1.181754779	4.3045824
322	0.032127429	1.157158666	13.10941
321	0.012394169	1.093971702	-12.80194
320	0.092182132	1.089505949	-23.36773
319	0.090328423	1.25380431	-16.32387
318	0.23565051	1.221647822	-16.32387
317	0.17000026	1.264030349	-16.32387
316	0.090788713	1.09429342	-26.88966
315	0.108929603	1.292563679	-26.88966
314	0.121459618	1.156614809	-26.88966
313	0.137796549	1.221816341	-26.88966
312	0.098338453	1.266542814	-36.95232
311	0.08658515	1.189008756	-47.51812
310	0.152819838	1.376478479	-49.27908
309	0.067161991	1.25881392	-49.27908
308	0.121522365	1.398730646	-49.27908
307	0.138073081	1.34100523	-59.84488
306	0.136605263	1.214769183	-69.90754
305	0.120008831	1.334103612	-69.90754

λ [nm]	Δε for enantiomer **2a** [M^{-1}cm^{-1}]	Δε for enantiomer **2b** [M^{-1}cm^{-1}]	ε for racemate [M^{-1}cm^{-1}]
304	0.143764631	1.393230799	-80.47333
303	0.080785138	1.486483084	-80.47333
302	0.159647098	1.35946572	-73.42947
301	0.072886259	1.439956521	-94.05792
300	0.193618018	1.449654023	-83.99526
299	0.146004231	1.436379322	-94.05792
298	0.101073302	1.371836547	-86.23141
297	0.12236003	1.414655692	-79.18754
296	0.22496524	1.448642909	-62.08102
295	0.11263478	1.383196259	-47.21065
294	0.104208382	1.607165665	-38.40582
293	0.046760884	1.608513817	-14.73062
292	0.040839637	1.673983447	36.616902
291	0.050342361	1.858381528	88.747072
290	-0.079327628	1.769786496	140.09459
289	-0.001495472	1.853555757	233.00648
288	-0.135481654	1.848684026	328.65766
287	-0.091686883	1.965980907	440.94018
286	-0.06336135	2.025322573	595.76538
285	-0.127628937	2.090286646	759.39542
284	-0.39391898	2.234883605	915.98159
283	-0.466600416	2.428435332	1115.1104
282	-0.59709059	2.51926707	1271.6966
281	-0.655068296	2.678057924	1435.3267
280	-0.786082852	2.708215165	1616.8459

λ [nm]	Δε for enantiomer **2a** [M^{-1}cm^{-1}]	Δε for enantiomer **2b** [M^{-1}cm^{-1}]	ε for racemate [M^{-1}cm^{-1}]
279	-0.822215834	2.816151582	1808.9309
278	-0.927459533	2.829204144	2023.9084
277	-0.885567324	3.050438946	2260.2971
276	-0.938534279	2.731218008	2476.0573
275	-0.74992746	2.897615196	2719.4898
274	-0.773381181	2.907335678	2946.7941
273	-0.747274182	2.904179777	3187.4873
272	-0.890004393	3.063614067	3439.7247
271	-0.978113833	3.202626919	3691.962
270	-1.00967529	3.481816936	3935.3946
269	-1.015748248	3.564261024	4239.7621
268	-1.122466488	3.676349137	4536.3031
267	-1.338811713	3.647976666	4892.0181
266	-1.426172677	3.7495783	5292.0368
265	-1.517656979	3.90565753	5716.9885
264	-1.758424013	4.087359308	6167.0969
263	-1.923998408	4.365974828	6673.6121
262	-2.196896098	4.665563303	7203.5229
261	-2.434790242	4.962677613	7785.0608
260	-2.615984017	5.284847633	8393.0132
259	-2.84245831	5.315334252	9008.7921
258	-3.14932423	5.568756182	9593.0693
257	-3.085977223	5.910213967	10164.545
256	-3.357808223	6.19173261	10748.822
255	-3.441776398	6.05408323	11268.949

λ [nm]	Δε for enantiomer **2a** [$M^{-1}cm^{-1}$]	Δε for enantiomer **2b** [$M^{-1}cm^{-1}$]	ε for racemate [$M^{-1}cm^{-1}$]
254	-3.38296058	6.027549148	11764.619
253	-3.149897912	6.12797115	12208.942
252	-2.668395218	5.865433874	12608.96
251	-2.450700945	5.52045251	12954.11
250	-2.049580915	4.695000508	13286.149
249	-1.302530828	4.215571624	13606.366
248	-0.664072409	3.385286191	13990.536
247	0.117250229	2.608742997	14450.707
246	1.048497405	1.70522074	14998.507
245	1.823855099	0.804088389	15695.066
244	2.629846498	0.021936041	16474.475
243	3.132059997	-0.975326669	17291.619
242	3.51348661	-1.26380055	18084.613
241	4.009663005	-1.526069728	18796.043
240	4.298534145	-2.236354316	19448.299
239	4.533160992	-2.471085957	20100.555
238	4.712929527	-2.840877914	20804.941
237	4.994974751	-3.574104066	21511.563
236	5.701141054	-4.384580857	22226.012
235	6.416315953	-6.049770676	23026.05
234	7.902868628	-7.520029976	23889.761
233	9.003172179	-9.340165352	24762.557
232	9.671869897	-11.00942261	25680.635
231	10.45364565	-12.21816341	26533.781
230	10.48725982	-12.97312851	27398.751

λ [nm]	Δε for enantiomer **1a** [$M^{-1}cm^{-1}$]	Δε for enantiomer **1b** [$M^{-1}cm^{-1}$]	ε for racemate [$M^{-1}cm^{-1}$]
229	10.54978216	-13.0921642	28280.352
228	10.00711513	-12.84635626	29276.471
227	9.506084853	-12.41173045	30298.027
226	8.693159973	-12.1177414	31339.736
225	7.82223946	-11.36438489	32383.681
224	6.880549932	-10.81708115	33461.364
223	6.021909792	-9.625268854	34530.242
222	4.868809598	-8.693512005	35515.013
221	4.213987806	-7.991936785	36495.284
220	3.421863369	-7.068100329	37342.336
219	2.596958402	-6.70881017	38046.89
218	2.509646738	-6.430623607	38535.488
217	1.87287351	-6.343851644	38853.133
216	1.511359926	-6.164286994	39071.604
215	1.393629687	-5.604581788	39191.378
214	1.107676257	-5.101996863	39151.742
213	0.432328761	-4.946193391	38917.086
212	0.123965442	-3.523609647	38505.887
211	-0.460666396	-3.056069012	37916.774
210	-0.870167556	-1.972874866	37061.252
209	-1.519095665	-1.421871369	35726.664
208	-1.85296048	0.014172065	33879.02
207	-1.95702096	1.172608794	31435.778
206	-2.350302167	1.254600945	28186.405
205	-2.389155656	1.628483318	24109.099

λ [nm]	Δε for enantiomer **2a** [$M^{-1}cm^{-1}$]	Δε for enantiomer **2b** [$M^{-1}cm^{-1}$]	ε for racemate [$M^{-1}cm^{-1}$]
204	-2.282159539	1.853165099	19545.068
203	-2.544166241	1.322353242	14430.525
202	-2.122304033	1.128058499	9927.5968
201	-1.804771219	0.765052496	6026.1358
200	-1.415797106	0.335663796	3348.4061
199	-1.591406449	-0.47806771	
198	-1.080462232	0.072434367	
197	-1.043047429	-0.277738455	
196	-0.853956566	-0.157888511	
195	-1.07347945	-0.301684239	
194	-0.875577195	-0.104530035	
193	-1.129086237	-0.194461729	
192	-0.925438201	0.034571672	
191	-0.700389149	0.401197004	
190	-0.635840996	0.398580363	

Calculated CD: Data

The DFT calculations were performed in Gaussian 09 (Revision C.01) suite of electronic structure programs. The atomic coordinates for both Geländer systems were taken from the single-crystal X-ray data and were used without further structural optimization as input for the calculations. The CD spectra were calculated using TD-B3LYP/6-31G**. The calculated data are listed below. This type of calculation provides only the electronic energy of the molecules (at 0 K) and fully neglects their vibrational motion and thermal corrections to both the enthalpy and the entropy. Consequently, these electronic energies are not suited as proxy for the thermodynamic stability of the structures **1** and **2**.

a)

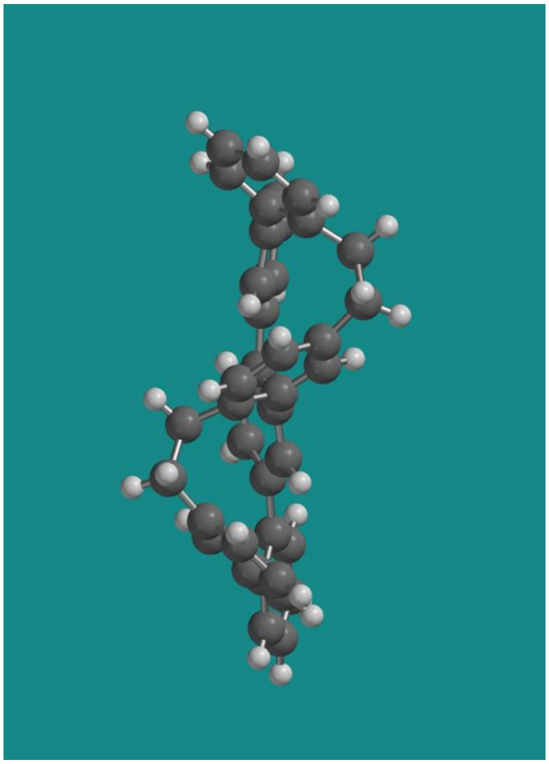

b)

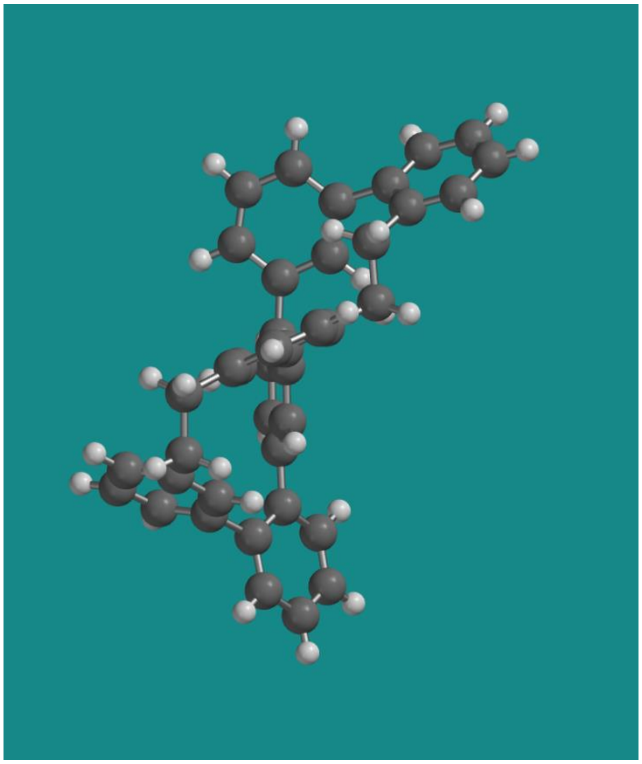

Figure S5: The input-conformations of the Geländer structures a) **1** and b) **2** used for the calculations of the CD signals.

Table S3: Calculated CD data of compound **1**.

Wavelength (nm)	Energy (cm^{-1})	R(length)
427.989206	23365.0753	0
362.601097	27578.5156	0
344.075576	29063.3823	0
338.504909	29541.6691	0
331.004066	30211.1092	0
319.127418	31335.4461	0
311.197493	32133.935	0
307.97405	32470.2682	43.0021
299.420868	33397.8058	0
297.53826	33609.1231	0
294.583238	33946.2628	0
292.857599	34146.2883	0
286.927387	34852.0234	0
286.516287	34902.0298	-0.6262
286.192219	34941.551	0
283.379487	35288.3694	0
278.17858	35948.1309	0
276.448066	36173.1596	0
276.392601	36180.4186	-2.5977
275.88828	36246.556	0
274.434886	36438.516	-11.5485
273.56897	36553.8533	0
272.062216	36756.2984	0
271.650913	36811.9507	-21.0956

270.324197	36992.6189	-39.3878
269.677418	37081.3399	0
269.022052	37171.674	0
265.63867	37645.1214	0
262.784157	38054.0445	-6.9059
261.553473	38233.0996	0
258.850459	38632.344	0
256.754526	38947.7068	-21.8375
253.536037	39442.1247	13.1111
253.168466	39499.3901	0
251.928705	39693.7697	0
250.68582	39890.5689	-5.2866
250.079053	39987.3555	0
249.963091	40005.9062	4.8482
249.189414	40130.1156	7.6848
248.082504	40309.1707	0
247.182346	40455.9636	0
244.882862	40835.8507	-4.6517
244.771668	40854.4015	0
243.36394	41090.7219	-4.4254
242.963341	41158.4725	8.416
242.251256	41279.4557	7.6248
241.50099	41407.6978	0
240.447198	41589.1726	0
240.437872	41590.7857	7.4252
239.800772	41701.2836	0

239.541322	41746.4507	-4.2533
238.577958	41915.0206	0
238.21583	41978.7384	4.4492
237.040805	42186.8294	0
236.507245	42282.0028	-15.6532
236.007525	42371.5304	-16.4111
235.756214	42416.6974	0
235.157031	42524.7757	-40.1044
234.321502	42676.4079	0
233.883898	42756.2568	0
233.320524	42859.4958	22.5002
232.123627	43080.4917	0
231.954264	43111.9473	-56.292
231.205954	43251.4813	8.4116
230.784195	43330.5236	-5.4135
230.676849	43350.6875	0
229.532348	43566.844	18.0007
229.358257	43599.9128	0
228.795337	43707.1845	20.0719
228.538079	43756.3843	0
228.092413	43841.8791	0
227.023223	44048.357	-34.1893
226.807268	44090.2979	3.313
226.459283	44158.0484	0
226.001081	44247.576	0
225.577558	44330.6511	0

225.282444	44388.723	-9.4881
224.426089	44558.0994	0
224.052972	44632.3024	7.0401
223.556064	44731.5086	0
222.957063	44851.6852	-9.0882
222.840852	44875.0753	0
222.241688	44996.0585	56.6746
222.082455	45028.3207	-3.7202
221.526931	45141.2383	4.7921
221.455709	45155.7563	0
221.179165	45212.2151	0
220.883635	45272.7067	0
220.773506	45295.2902	-5.114
220.584968	45334.0048	-29.0034
220.353665	45381.5915	5.7425
220.158024	45421.9192	0
219.437166	45571.1318	16.8659
219.12303	45636.4627	0
218.2244	45824.3899	0
218.093886	45851.8127	9.0087
217.645952	45946.1796	0
217.558113	45964.7304	-5.0705
217.154204	46050.2251	-24.4066
217.127584	46055.871	0
216.683607	46150.2379	0
216.381076	46214.7622	6.3081

216.045503	46286.5456	14.4941
215.665941	46368.0076	-3.9355
215.527228	46397.8501	0
214.858666	46542.2233	0
214.735864	46568.8396	3.5671
214.375712	46647.0754	0
214.135048	46699.5014	21.6919
214.02785	46722.8915	0
213.880165	46755.1537	14.42
213.027599	46942.2743	-2.0559
212.888602	46972.9234	0
212.775344	46997.9266	0
212.596569	47037.4477	-41.8396
212.33806	47094.7131	-21.9506
212.236285	47117.2966	0
212.09469	47148.7523	0
212.054787	47157.6244	-20.5352
211.400353	47303.6107	0
211.040516	47384.2661	16.0843
210.724872	47455.2429	0
210.431598	47521.3804	-7.3584
210.324506	47545.577	0
210.306668	47549.6098	-1.4476
210.082168	47600.4227	-6.3189
210.021671	47614.1342	0
209.609794	47707.6945	-27.0265

209.146595	47813.3531	-6.1877
208.942168	47860.1333	1.7225
208.854176	47880.2971	0
208.474901	47967.405	0
208.090015	48056.126	0
207.723948	48140.8142	0
207.397322	48216.6303	14.2098
207.203224	48261.7974	0
207.092474	48287.6071	51.8333
206.660988	48388.4264	6.7357
206.64721	48391.6526	0
206.354864	48460.2098	-2.3096
206.138717	48511.0227	22.598
205.864897	48575.547	0
205.690716	48616.6813	0
204.814063	48824.7724	8.2252
204.682195	48856.228	5.4267
204.6349	48867.5197	0
204.405488	48922.3654	0
203.673478	49098.1943	0
203.379471	49169.1711	0
203.332775	49180.4629	6.3281
203.162851	49221.5971	18.8464
203.159522	49222.4037	0
202.866996	49293.3805	-1.221
202.694535	49335.3213	-1.1181

202.677967	49339.3541	0
202.337282	49422.4292	0
202.080049	49485.3404	0
201.807043	49552.2845	0.1756
201.433272	49644.2317	10.0995
200.842663	49790.218	0
200.589214	49853.1293	-34.6207
200.433562	49891.8439	0
200.329929	49917.6536	48.3373
199.687856	50078.1579	-7.1595
199.687856	50078.1579	0
199.164996	50209.6263	43.5181
199.088241	50228.9836	0
198.893423	50278.1834	0
198.762694	50311.2522	2.6951
198.663964	50336.2554	0
198.339801	50418.5239	-12.5934
198.159112	50464.4975	0
198.070473	50487.081	-3.3853
197.433347	50650.005	36.0863
197.351638	50670.9754	15.3335
197.175879	50716.1425	0
197.119452	50730.6605	-42.8029
196.8503	50800.0242	0
196.345284	50930.686	-16.9337
196.301762	50941.9777	0

196.193042	50970.2071	0
196.016241	51016.1807	-2.6179
195.876887	51052.4757	0
195.700655	51098.4493	0
195.438442	51167.0064	13.9237
195.352219	51189.5899	0
195.241474	51218.6259	1.8705
194.962092	51292.0224	0
194.93757	51298.4748	9.1137
194.503315	51413.0055	0
194.372197	51447.6874	5.4151
193.955624	51558.1853	-43.2908
193.335609	51723.529	0
193.263282	51742.8863	33.3251
192.89645	51841.2859	0
192.638699	51910.6496	-52.7778
192.083587	52060.6687	0
191.982461	52088.0916	1.061
191.825035	52130.839	0
191.78646	52141.3242	0
191.727145	52157.4553	-6.0378
191.519831	52213.9141	0
191.180215	52306.6678	-5.8496
190.85649	52395.3888	34.5422
190.718505	52433.2969	14.3726
190.665713	52447.8149	0

190.396341	52522.0179	0
189.982061	52636.5486	-2.1885
189.833711	52677.6829	0
189.662378	52725.2696	-64.3622
189.364012	52808.3447	0
189.34666	52813.184	-5.1327
188.669547	53002.7243	0
188.534705	53040.6324	0
188.365709	53088.2191	-125.3621
188.357124	53090.6387	-16.7972
188.088524	53166.4549	0
187.681375	53281.7921	0
187.052778	53460.8472	-3.9155
187.021741	53469.7193	0
186.534962	53609.2532	5.1345
185.808133	53818.9574	9.8183
185.774724	53828.636	0
185.399696	53937.5209	0
185.360891	53948.8126	133.7476
184.921313	54077.0548	-44.8693
184.910282	54080.281	0
184.108509	54315.7949	71.4213
183.996487	54348.8636	0
183.821897	54400.4831	-19.746
183.511727	54492.4303	0
183.457419	54508.5614	-31.8469

182.988994	54648.0953	0
182.482659	54799.7276	0
182.364559	54835.216	61.264
182.249292	54869.8978	0
182.1529	54898.9338	-19.1802
181.385424	55131.2214	6.3911
181.358892	55139.287	0
181.242242	55174.7754	0
180.977686	55255.4308	9.1649
180.935428	55268.3357	13.9597
180.872079	55287.693	0
180.600709	55370.7681	0
180.429875	55423.1941	1.4563
180.212202	55490.1382	18.1357
180.175538	55501.4299	0
180.06302	55536.1118	43.0342
179.788856	55620.8	5.0072
179.67682	55655.4818	0
179.507729	55707.9079	0
179.178266	55810.3403	0
179.051474	55849.8614	5.3453
178.927443	55888.576	68.1202
178.715954	55954.7135	0
178.659298	55972.4577	120.1532
177.872422	56220.0699	0
177.503175	56337.0203	0

177.216479	56428.161	24.2109
176.749103	56577.3735	0
176.221546	56746.75	-31.3798
176.151443	56769.3335	0
175.866598	56861.2807	-47.3276
175.515562	56975.0049	0
175.212958	57073.4045	0
175.18325	57083.0832	18.3622
175.148603	57094.3749	92.9776
174.857125	57189.5483	0
174.714211	57236.3285	-117.5192
174.694518	57242.7809	0
174.340785	57358.9248	45.4114
173.995808	57472.6489	-77.6851
173.70085	57570.242	0
173.375368	57678.3203	-90.8348
173.351127	57686.3859	0
173.036612	57791.2379	0
172.771373	57879.9589	-125.0222
172.706394	57901.7359	0
172.456558	57985.6176	45.2091
172.329515	58028.3649	467.6014
172.021079	58132.4105	0
171.947124	58157.4137	-302.2472
171.782741	58213.0659	0
171.381446	58349.3736	66.1444

171.34355	58362.2785	0
170.72304	58574.4023	0
170.638452	58603.4383	-66.274
170.102339	58788.1392	106.2437
170.076672	58797.0113	45.8712
168.954926	59187.3837	-31.2406
168.722706	59268.8457	-84.5172
168.479675	59354.3404	-73.0377
168.433899	59370.4715	-7.4521
167.521305	59693.8998	-84.8454
166.89217	59918.9285	-125.7759
166.74179	59972.9677	101.916
166.374838	60105.2426	128.2196
165.57497	60395.6022	-23.2358
164.94498	60626.2768	17.4697
164.509451	60786.7811	-1.8806

Table S4: Calculated CD data of compound **2**.

**Wavelength (nm)**	**Energy (cm^{-1})**	**R(length)**
363.707334	27494.6339	0
346.431007	28865.7765	0
343.484577	29113.3887	0
332.932849	30036.0869	0
326.583587	30620.0323	0
321.903087	31065.2504	0
289.095047	34590.6998	0

285.875474	34980.2656	0
281.801471	35485.9752	0
277.873088	35987.6521	0
276.82458	36123.9598	0
275.857588	36250.5888	0
274.653744	36409.48	0
270.365461	36986.973	0
269.824141	37061.176	0
268.998705	37174.9002	0
266.741664	37489.4564	0
266.157596	37571.725	0
264.150229	37857.2452	0
262.250551	38131.4737	-22.4819
260.323331	38413.7678	-86.4164
256.573874	38975.1297	0
255.769351	39097.7259	0
255.379499	39157.411	-46.8356
252.905093	39540.5243	0
250.914118	39854.274	0
250.817675	39869.5985	53.8753
249.17439	40132.5353	0
247.894018	40339.8198	16.5149
247.133076	40464.0291	0
245.620256	40713.2545	15.75
244.578528	40886.6637	1.3003
243.785035	41019.7452	-33.6969

242.312805	41268.9705	0
241.769418	41361.7242	-65.1498
240.461188	41586.7529	41.1507
239.495051	41754.5162	0
239.411808	41769.0342	6.3047
239.047147	41832.752	0
237.927832	42029.5513	0
237.058934	42183.6032	3.8185
236.124387	42350.56	105.5005
235.112438	42532.8413	0
234.702974	42607.0443	12.1991
233.333697	42857.0761	-48.5721
233.281013	42866.7548	0
231.923891	43117.5932	1.4127
231.685527	43161.9537	5.2067
231.451973	43205.5077	0
231.132682	43265.1927	0
230.767013	43333.7498	-27.4986
229.860014	43504.7393	0
229.672662	43540.2277	9.1122
228.900938	43687.0206	0
227.91212	43876.5609	0
227.828359	43892.692	69.2858
227.623406	43932.2132	-9.8098
227.085595	44036.2587	0
226.915194	44069.3274	0

226.116489	44224.9925	1.6933
225.528318	44340.3297	-32.3995
225.397119	44366.1395	0
225.15153	44414.5327	0
224.910556	44462.1195	15.9748
224.462657	44550.8404	-59.7783
224.3124	44580.683	0
224.259655	44591.1682	36.2278
223.64478	44713.7644	0
223.354698	44771.8364	33.4471
222.912968	44860.5573	18.7377
222.476974	44948.4718	0
221.81625	45082.3598	44.562
221.645738	45117.0416	0
220.679197	45314.6475	-1.1528
220.189303	45415.4668	0
220.048617	45444.5027	35.0319
219.542078	45549.3548	0
219.39057	45580.8104	-14.1461
219.119158	45637.2693	-11.6777
218.945033	45673.5642	0
218.659294	45733.2492	0.2428
218.586049	45748.5738	0
218.028687	45865.5242	-62.9442
217.963527	45879.2356	0
217.302638	46018.7695	19.84

217.268366	46026.0285	0
216.657102	46155.8838	0
216.600327	46167.9821	17.7621
216.396183	46211.536	0
216.13966	46266.3817	11.0533
216.113287	46272.0276	0
216.030445	46289.7718	0
216.007863	46294.6111	-5.5535
215.452322	46413.9812	-6.5042
215.149484	46479.3121	0
215.030079	46505.1218	21.7246
214.691243	46578.5183	0
214.672657	46582.551	7.6656
214.190538	46687.4031	14.6727
214.138747	46698.6949	0
213.743739	46784.9962	0.9194
213.684797	46797.9011	0
213.29146	46884.2024	0
213.001981	46947.9202	0
212.881292	46974.5365	-9.1365
212.786299	46995.5069	-17.2052
212.640323	47027.7691	10.5682
212.225386	47119.7163	0
212.218121	47121.3294	14.6679
211.783122	47218.1159	0
211.67465	47242.3126	24.2305

211.447222	47293.1255	6.3679
211.065665	47378.6203	0
210.871816	47422.1742	0
210.853885	47426.207	0
210.285266	47554.4491	4.7198
209.964764	47627.039	0
209.943432	47631.8784	9.8824
209.719706	47682.6913	0
209.549568	47721.4059	0
209.52832	47726.2452	-10.1045
208.952732	47857.7136	0
208.896403	47870.6185	-0.8407
208.724084	47910.1396	40.1351
208.338279	47998.8606	0
208.107479	48052.0932	-19.9525
208.009719	48074.6767	0
207.905078	48098.8734	0
207.553557	48180.3354	-12.1484
207.324493	48233.568	-10.5174
207.078638	48290.8333	0
206.740246	48369.8757	-2.1138
206.588675	48405.3641	0
206.447637	48438.4328	22.8329
206.224436	48490.8588	0
206.186711	48499.7309	26.6439
206.066769	48527.9603	38.6221

206.049646	48531.9931	0
205.584985	48641.6845	-69.0587
205.37385	48691.6909	0
205.105449	48755.4087	0
205.068133	48764.2808	0
205.017268	48776.3791	23.3475
204.641655	48865.9066	-5.6157
204.489771	48902.2016	17.7195
204.32801	48940.9162	0
204.233767	48963.4997	0
204.112726	48992.5357	0
203.88113	49048.1879	22.1525
203.867721	49051.4142	0
203.646715	49104.6467	0
203.486284	49143.3614	2.3565
203.066354	49244.9872	33.3568
202.926762	49278.8625	17.4731
202.876954	49290.9608	0
202.57531	49364.3573	0
202.492598	49384.5211	-74.3794
202.324075	49425.6554	5.5017
202.066875	49488.5667	0
201.895771	49530.5075	29.733
201.656056	49589.386	0
201.613427	49599.8712	-6.5698
201.534774	49619.2285	0

201.214244	49698.2708	2.2004
200.875203	49782.1525	0
200.823145	49795.0573	-0.5783
200.462729	49884.5849	0
200.304037	49924.106	13.3897
200.17468	49956.3682	0
200.009991	49997.5025	-34.3461
199.453353	50137.0364	0
199.341115	50165.2658	21.8662
199.321886	50170.1051	0
199.113819	50222.5312	0
198.957256	50262.0524	-10.5589
198.298562	50429.0091	0
198.270022	50436.2681	-5.8934
198.105286	50478.2089	16.123
198.057816	50490.3073	0
197.707249	50579.8348	4.6459
197.669424	50589.5135	0
197.543446	50621.7756	0
197.423915	50652.4247	5.5439
197.285692	50687.9131	2.46
197.135123	50726.6277	-1.9763
196.931594	50779.0537	0
196.790935	50815.3487	0
196.560066	50875.0337	0
196.538255	50880.6796	-1.0764

196.155794	50979.8858	9.0441
195.790277	51075.0592	-0.7583
195.787185	51075.8658	0
195.518573	51146.036	39.583
195.259923	51213.7866	0
195.247623	51217.0128	0
195.133925	51246.8553	-3.4051
194.824232	51328.3173	-16.0521
194.656001	51372.6778	15.2864
194.585735	51391.2286	0
194.478907	51419.458	0
194.250385	51479.9495	-0.9225
193.937421	51563.0246	0
193.643608	51641.2604	-13.7181
193.616392	51648.5194	0
193.3537	51718.6897	0
193.037605	51803.3779	-1.2612
192.782475	51871.935	0
192.659653	51905.0037	-2.1958
192.504104	51946.9446	1.8294
191.934909	52100.9965	32.196
191.90223	52109.8685	0
191.647128	52179.2322	0
191.626394	52184.8781	50.8145
191.233293	52292.1499	-75.4621
191.082982	52333.2841	0

190.847676	52397.8085	0
190.786005	52414.7461	64.7109
190.756651	52422.8117	0
190.677442	52444.5886	-4.6498
190.355416	52533.3096	-213.5613
189.877319	52665.5846	46.7162
189.514526	52766.4039	-5.5668
189.430556	52789.7939	0
189.268617	52834.961	0
189.031992	52901.0984	0
189.017582	52905.1312	2.2719
188.640841	53010.7898	15.5044
188.319931	53101.1239	0
188.059994	53174.5204	-54.092
188.051437	53176.9401	0
187.732527	53267.2742	0
187.522412	53326.9592	15.5955
187.281643	53395.5163	0
187.185508	53422.9392	32.7677
186.473241	53626.9974	0
186.324717	53669.7448	40.7982
186.207186	53703.6201	0
185.90007	53792.3411	-74.3225
185.410787	53934.2947	-12.9703
185.396924	53938.3274	0
184.957175	54066.5696	67.116

184.940622	54071.4089	0
184.811056	54109.317	0
184.258996	54271.4344	3.4163
183.838251	54395.6438	-47.8155
183.612281	54462.5878	-82.1589
183.54161	54483.5582	0
183.435705	54515.0139	85.9091
183.411283	54522.2728	0
182.897215	54675.5182	-157.701
182.883726	54679.551	0
182.410171	54821.5045	22.7155
182.027209	54936.8418	0
181.741708	55023.1431	401.4557
181.475692	55103.7986	0
181.313805	55152.9984	0
181.3032	55156.2246	-5.1511
181.078126	55224.7818	3.5395
181.054328	55232.0407	0
180.537594	55390.1254	0
180.102254	55524.0134	166.8797
180.086559	55528.8528	0
179.437584	55729.6848	-196.2279
179.206754	55801.4682	0
178.623263	55983.7495	0
178.543523	56008.7527	32.4254
178.486976	56026.4969	0

178.043559	56166.0308	93.0754
177.576902	56313.6302	0
177.112685	56461.2297	81.7518
177.057041	56478.9739	0
177.034288	56486.2329	-238.5149
176.178977	56760.4614	-63.9437
175.991417	56820.953	0
175.946461	56835.4709	58.2011
175.639882	56934.6771	0
175.498171	56980.6507	0
175.269926	57054.8538	-98.0697
174.556785	57287.948	-155.4854
174.497823	57307.3053	0
174.367756	57350.0527	-348.3361
174.27952	57379.0886	0
174.073981	57446.8392	0
173.820176	57530.7209	316.0042
173.70085	57570.242	0
173.55982	57617.0222	263.133
173.450558	57653.3171	0
172.45176	57987.2307	-561.584
171.604419	58273.5575	-61.9777
170.960802	58492.9403	50.6059
170.878334	58521.1697	254.3714
170.375827	58693.7724	120.6075
170.011372	58819.5948	-61.7759

169.414343	59026.8793	-29.4088
168.504863	59345.4683	56.1321
167.657223	59645.5066	99.8047
167.412729	59732.6145	99.0974
167.205018	59806.8175	-64.158
166.726095	59978.6136	57.1186
166.107358	60202.0291	-60.9478

Determination of $\Delta G^{\neq}_{rac(298\,K)}$ by circular dichroism

See references[2,3,4] for calculation.

The rate of racemization between *M* and *P* enantiomer can be described as first order process with

$$ln[A]_t = -k_{rac}\,t + ln[A]_0 \qquad \textbf{(1)}$$

with $[A]_0$ being the concentration at $t = 0$, $[A]_t$ the concentration at observed time t and k_{rac} the rate constant.

Equation 1 can be brought into a linear form:

$$ln\left(\frac{[A]_t}{[A]_0}\right) = -k_{rac}\,t \qquad \textbf{(2)}$$

Plotting t against $ln\left(\frac{[A]_t}{[A]_0}\right)$ gives access to k_{rac} directly from the slope and the half-live can be determined with the following equation:

$$t_{1/2} = \frac{\ln(2)}{k_{rac}} \qquad \textbf{(3)}$$

The obtained k_{rac} can be then used to caluclate the free Gibbs energy of enantiomerization $G^{\neq}_{rac(T)}$ by the rearranged Eyring equation:

$$\Delta G^{\neq}_{rac(298.15)} = -RTln\left(\frac{hk_{rac}}{\kappa k_B T}\right) \textbf{(4)}$$

Where k_{rac} is the obtained kinetic rate constant at RT, κ the transition factor ($\kappa = 0.5$), k_B the Boltzmann constant (k_B = 1.380662 x 10^{-23} J K^{-1}), h the Planck's constant (h = 6.626176 x 10^{-34} J

---

[3] M. Rickhaus, L. M. Bannwart, M. Neuburger, H. Gsellinger, K. Zimmermann, D. Häussinger, M. Mayor, *Angew. Chem. Int. Ed.*, 2014, **53**, 14587–14591.
[4] M. Rickhaus, L. Jundt, M. Mayor, *CHIMIA*, 2016, **70**, 192–202.

s), R the universal gas constant (R = 8.31446 x 10^{-3} kJ K^{-1} mol^{-1}) and T the temperature (T = 298.15 K).

Table S5: Rate constants and half-lifes of compound **1** and **2** by ECD determined at 25 °C.

Temperature	Rate constant [1/s] of compound **1**		Half-life [h]		Rate constant [1/s] of compound **2**	Half-life [h]	
25 °C	k_{rac} =	2.09337E-06	τ =	91.98	6.22212E-05	τ =	3.09
	Racemization barrier [kJ mol^{-1}]						
	$\Delta G^{\neq}_{rac(298.15)}$ =	103.7			$\Delta G^{\neq}_{rac(298.15)}$ =	95.3	

Table S6: Mdeg values of compound **1** at 247 nm.

time [s]	Intensity A (mdeg)	Intensity B (mdeg)	$[A]_t$	$ln\left(\frac{[A]_t}{[A]_0}\right)$
63.2071	13.0933	0	13.0933	0
128.634	13.0554	0.0379	13.0554	-0.00289881
196.358	13.0549	0.0384	13.0549	-0.00293711
266.46	13.0741	0.0192	13.0741	-0.00146748
339.024	13.0606	0.0327	13.0606	-0.00250058
414.136	13.0842	0.0091	13.0842	-0.00069525
491.885	13.0805	0.0128	13.0805	-0.00097808
572.365	13.0826	0.0107	13.0826	-0.00081755
655.671	13.0617	0.0316	13.0617	-0.00241637
741.902	13.0813	0.012	13.0813	-0.00091692
831.161	13.1066	-0.0133	13.1066	0.00101527
923.554	13.0848	0.0085	13.0848	-0.0006494
1019.19	13.1153	-0.022	13.1153	0.00167884
1118.19	13.1136	-0.0203	13.1136	0.00154921

1220.66	13.1009	-0.0076	13.1009	0.00058028
1326.73	13.115	-0.0217	13.115	0.00165596
1436.53	13.1224	-0.0291	13.1224	0.00222004
1550.18	13.1148	-0.0215	13.1148	0.00164071
1667.82	13.1107	-0.0174	13.1107	0.00132804
1789.59	13.119	-0.0257	13.119	0.00196091
1915.64	13.1178	-0.0245	13.1178	0.00186944
2046.12	13.103	-0.0097	13.103	0.00074056
2181.17	13.1075	-0.0142	13.1075	0.00108394
2320.97	13.0983	-0.005	13.0983	0.0003818
2465.68	13.1025	-0.0092	13.1025	0.0007024
2615.47	13.1026	-0.0093	13.1026	0.00071003
2770.52	13.0834	0.0099	13.0834	-0.0007564
2931.01	13.0823	0.011	13.0823	-0.00084048
3097.14	13.0894	0.0039	13.0894	-0.00029791
3269.11	13.0843	0.009	13.0843	-0.00068761
3447.11	13.0923	0.001	13.0923	-7.6378E-05
3631.36	13.074	0.0193	13.074	-0.00147512
3822.08	13.0882	0.0051	13.0882	-0.00038959
4019.5	13.0789	0.0144	13.0789	-0.0011004
4223.85	13.063	0.0303	13.063	-0.00231684
4435.38	13.0641	0.0292	13.0641	-0.00223264
4654.34	13.0598	0.0335	13.0598	-0.00256184
4880.98	13.0619	0.0314	13.0619	-0.00240105
5115.59	13.0433	0.05	13.0433	-0.00382606
5358.43	13.0385	0.0548	13.0385	-0.00419413

5609.8	13.0215	0.0718	13.0215	-0.00549881
5869.99	13.0277	0.0656	13.0277	-0.00502279
6139.32	13.0136	0.0797	13.0136	-0.00610568
6418.11	13.0228	0.0705	13.0228	-0.00539898
6706.69	13.0155	0.0778	13.0155	-0.00595969
7005.41	12.9947	0.0986	12.9947	-0.00755907
7314.61	12.9829	0.1104	12.9829	-0.00846754
7634.67	12.9706	0.1227	12.9706	-0.00941539
7965.97	12.974	0.1193	12.974	-0.00915329
8308.9	12.9652	0.1281	12.9652	-0.0098318
8663.88	12.9455	0.1478	12.9455	-0.01135241
9031.31	12.9523	0.141	12.9523	-0.01082727
9411.66	12.9298	0.1635	12.9298	-0.01256592
9805.36	12.9267	0.1666	12.9267	-0.01280571
10212.9	12.9183	0.175	12.9183	-0.01345574
10634.7	12.9072	0.1861	12.9072	-0.01431535
11071.4	12.8997	0.1936	12.8997	-0.01489659
11523.3	12.8748	0.2185	12.8748	-0.01682874
11991.2	12.8693	0.224	12.8693	-0.01725602
12475.5	12.8608	0.2325	12.8608	-0.01791672
12976.7	12.8427	0.2506	12.8427	-0.01932509
13495.6	12.8257	0.2676	12.8257	-0.02064968
14032.7	12.813	0.2803	12.813	-0.02164037
14588.7	12.7994	0.2939	12.7994	-0.02270235
15164.2	12.7762	0.3171	12.7762	-0.02451658
15759.9	12.771	0.3223	12.771	-0.02492367

16376.5	12.7462	0.3471	12.7462	-0.02686746
17014.8	12.7361	0.3572	12.7361	-0.02766017
17675.5	12.7033	0.39	12.7033	-0.03023885
18359.3	12.6925	0.4008	12.6925	-0.03108938
19067.2	12.6791	0.4142	12.6791	-0.03214568
19800	12.6516	0.4417	12.6516	-0.03431696
20558.5	12.6295	0.4638	12.6295	-0.0360653
21343.6	12.6062	0.4871	12.6062	-0.03791189
22156.3	12.5957	0.4976	12.5957	-0.03874516
22997.5	12.5561	0.5372	12.5561	-0.04189405
23868.3	12.5358	0.5575	12.5358	-0.0435121
24769.6	12.5189	0.5744	12.5189	-0.04486115
25702.6	12.4791	0.6142	12.4791	-0.0480454
26668.4	12.464	0.6293	12.464	-0.04925616
27668.1	12.4363	0.657	12.4363	-0.05148103
28702.8	12.4049	0.6884	12.4049	-0.05400909
29773.9	12.3735	0.7198	12.3735	-0.05654356
30882.7	12.3457	0.7476	12.3457	-0.05879282
32030.3	12.3182	0.7751	12.3182	-0.06102281
33218.3	12.2813	0.812	12.2813	-0.06402287
34447.9	12.2494	0.8439	12.2494	-0.06662369
35720.8	12.2073	0.886	12.2073	-0.07006652
37038.3	12.173	0.9203	12.173	-0.07288026
38402.1	12.1333	0.96	12.1333	-0.07614691
39813.8	12.0915	1.0018	12.0915	-0.07959792
41275.1	12.0528	1.0405	12.0528	-0.08280365

42787.7	12.0132	1.0801	12.0132	-0.0860946
44353.4	11.9666	1.1267	11.9666	-0.08998121
45974.1	11.9241	1.1692	11.9241	-0.09353909
47651.7	11.8805	1.2128	11.8805	-0.09720225
49388.2	11.8292	1.2641	11.8292	-0.1015296
51185.6	11.7769	1.3164	11.7769	-0.10596066
53046.2	11.7304	1.3629	11.7304	-0.10991689
54972.2	11.6895	1.4038	11.6895	-0.11340965
56965.7	11.6246	1.4687	11.6246	-0.11897711
59029.3	11.5661	1.5272	11.5661	-0.12402224
61165.3	11.5039	1.5894	11.5039	-0.12941454
63376.3	11.4478	1.6455	11.4478	-0.13430308
65665	11.391	1.7023	11.391	-0.13927708
68034	11.3305	1.7628	11.3305	-0.14460244
70486.3	11.2727	1.8206	11.2727	-0.14971678
73024.6	11.2166	1.8767	11.2166	-0.15470583
75652.1	11.156	1.9373	11.156	-0.16012318
78371.8	11.0965	1.9968	11.0965	-0.16547091
81187	11.0282	2.0651	11.0282	-0.17164502
84101.1	10.9581	2.1352	10.9581	-0.17802174
87117.6	10.8908	2.2025	10.8908	-0.18418225
90239.9	10.823	2.2703	10.823	-0.19042715
93471.9	10.7471	2.3462	10.7471	-0.1974647
96817.4	10.6572	2.4361	10.6572	-0.20586493
100280	10.5608	2.5325	10.5608	-0.21495162
103865	10.4817	2.6116	10.4817	-0.22246977

107575	10.3952	2.6981	10.3952	-0.23075649
111416	10.3072	2.7861	10.3072	-0.23925797
115392	10.2173	2.876	10.2173	-0.24801829
119507	10.1291	2.9642	10.1291	-0.25668818
123767	10.0349	3.0584	10.0349	-0.26603163
128176	9.94386	3.14944	9.94386	-0.27514537
132740	9.85028	3.24302	9.85028	-0.28460077
137464	9.75893	3.33437	9.75893	-0.29391789
142355	9.64129	3.45201	9.64129	-0.30604573
147417	9.55483	3.53847	9.55483	-0.31505386
152656	9.49494	3.59836	9.49494	-0.32134162
158080	9.43776	3.65554	9.43776	-0.32738199
163694	9.36275	3.73055	9.36275	-0.3353616
169506	9.27322	3.82008	9.27322	-0.34496997
175521	9.15954	3.93376	9.15954	-0.35730469
181748	9.04513	4.04817	9.04513	-0.36987416
188193	8.81684	4.27646	8.81684	-0.39543712
194865	8.69561	4.39769	8.69561	-0.40928235
201770	8.5915	4.5018	8.5915	-0.42132731
208919	8.50937	4.58393	8.50937	-0.43093274
216318	8.41786	4.67544	8.41786	-0.44174501
223977	8.33915	4.75415	8.33915	-0.45113936
231906	8.16832	4.92498	8.16832	-0.47183739
240112	8.13924	4.95406	8.13924	-0.47540384
248607	7.97688	5.11642	7.97688	-0.49555329
259200	7.71642	5.37688	7.71642	-0.52875012

Table S7: Mdeg values of compound **2** at 229 nm.

time [s]	Intensity A (mdeg)	Intensity B (mdeg)	$[A]_t$	$ln\left(\frac{[A]_t}{[A]_0}\right)$
21.069	8.51805	0	8.51805	0
42.8779	8.52353	-0.00548	8.52353	0.00064313
65.4526	8.54713	-0.02908	8.54713	0.00340811
88.82	8.54136	-0.02331	8.54136	0.0027328
113.008	8.53156	-0.01351	8.53156	0.00158479
138.045	8.49947	0.01858	8.49947	-0.00218363
163.962	8.50213	0.01592	8.50213	-0.00187072
190.788	8.50456	0.01349	8.50456	-0.00158495
218.557	8.4782	0.03985	8.4782	-0.00468928
247.301	8.46502	0.05303	8.46502	-0.00624506
277.054	8.49522	0.02283	8.49522	-0.00268379
307.851	8.44473	0.07332	8.44473	-0.00864486
339.731	8.37267	0.14538	8.37267	-0.01721461
372.729	8.39681	0.12124	8.39681	-0.01433557
406.887	8.4042	0.11385	8.4042	-0.01345586
442.244	8.35453	0.16352	8.35453	-0.01938353
478.842	8.33319	0.18486	8.33319	-0.02194111
516.726	8.29158	0.22647	8.29158	-0.0269469
555.94	8.31964	0.19841	8.31964	-0.02356846
596.531	8.30248	0.21557	8.30248	-0.02563318
638.547	8.2512	0.26685	8.2512	-0.0318288
682.039	8.23598	0.28207	8.23598	-0.03367508
727.057	8.20154	0.31651	8.20154	-0.0378655

773.657	8.19563	0.32242	8.19563	-0.03858636
821.893	8.17245	0.3456	8.17245	-0.0414187
871.823	8.11189	0.40616	8.11189	-0.04885655
923.506	8.11228	0.40577	8.11228	-0.04880848
977.004	8.06421	0.45384	8.06421	-0.05475169
1032.38	8.04681	0.47124	8.04681	-0.0569117
1089.7	8.02019	0.49786	8.02019	-0.06022533
1149.04	8.01406	0.50399	8.01406	-0.06098994
1210.45	7.95688	0.56117	7.95688	-0.06815048
1274.03	7.93301	0.58504	7.93301	-0.07115491
1339.83	7.87028	0.64777	7.87028	-0.0790938
1407.95	7.80657	0.71148	7.80657	-0.08722175
1478.46	7.79467	0.72338	7.79467	-0.08874727
1551.45	7.73654	0.78151	7.73654	-0.09623288
1626.99	7.71914	0.79891	7.71914	-0.09848448
1705.2	7.65151	0.86654	7.65151	-0.10728443
1786.14	7.63309	0.88496	7.63309	-0.1096947
1869.93	7.57114	0.94691	7.57114	-0.11784379
1956.66	7.54606	0.97199	7.54606	-0.12116187
2046.44	7.51779	1.00026	7.51779	-0.12491523
2139.37	7.46169	1.05636	7.46169	-0.13240551
2235.56	7.40849	1.10956	7.40849	-0.1395608
2335.14	7.36221	1.15584	7.36221	-0.14582728
2438.2	7.32089	1.19716	7.32089	-0.15145554
2544.89	7.26602	1.25203	7.26602	-0.15897876
2655.32	7.20027	1.31778	7.20027	-0.16806892

2769.63	7.16103	1.35702	7.16103	-0.17353362
2887.96	7.09607	1.42198	7.09607	-0.18264633
3010.44	7.04221	1.47584	7.04221	-0.1902654
3137.22	6.96345	1.5546	6.96345	-0.2015124
3268.45	6.94325	1.5748	6.94325	-0.20441748
3404.29	6.86099	1.65706	6.86099	-0.2163357
3544.9	6.80369	1.71436	6.80369	-0.22472233
3690.45	6.76913	1.74892	6.76913	-0.22981487
3841.11	6.67886	1.83919	6.67886	-0.24324013
3997.06	6.61496	1.90309	6.61496	-0.25285369
4158.49	6.54604	1.97201	6.54604	-0.26332715
4325.58	6.47085	2.0472	6.47085	-0.27487997
4498.54	6.37551	2.14254	6.37551	-0.28972335
4677.58	6.30479	2.21326	6.30479	-0.30087778
4862.9	6.22769	2.29036	6.22769	-0.31318196
5054.73	6.14211	2.37594	6.14211	-0.32701911
5253.3	6.0522	2.46585	6.0522	-0.3417656
5458.84	5.97256	2.54549	5.97256	-0.3550118
5671.59	5.88805	2.63	5.88805	-0.36926257
5891.82	5.81334	2.70471	5.81334	-0.38203216
6119.78	5.71319	2.80486	5.71319	-0.3994099
6355.75	5.62997	2.88808	5.62997	-0.41408333
6600	5.55295	2.9651	5.55295	-0.42785812
6852.83	5.45768	3.06037	5.45768	-0.44516365
7114.54	5.37286	3.14519	5.37286	-0.46082709
7385.43	5.26767	3.25038	5.26767	-0.4805993

7665.84	5.15753	3.36052	5.15753	-0.50172966
7956.1	5.05241	3.46564	5.05241	-0.52232208
8256.54	4.9583	3.55975	4.9583	-0.5411245
8567.54	4.85351	3.66454	4.85351	-0.56248529
8889.46	4.7505	3.76755	4.7505	-0.58393757
9222.68	4.65776	3.86029	4.65776	-0.6036528
9567.61	4.5513	3.96675	4.5513	-0.62677453
9924.64	4.45342	4.06463	4.45342	-0.6485151
10294.2	4.33621	4.18184	4.33621	-0.67518675
10676.8	4.22191	4.29614	4.22191	-0.70189981
11072.8	4.10792	4.41013	4.10792	-0.72927062
11482.6	4.00276	4.51529	4.00276	-0.75520332
11906.9	3.88505	4.633	3.88505	-0.78505159
12346.1	3.79283	4.72522	3.79283	-0.809075
12800.7	3.68143	4.83662	3.68143	-0.83888618
13271.3	3.55774	4.96031	3.55774	-0.87306193
13758.4	3.44562	5.07243	3.44562	-0.90508358
14262.6	3.34427	5.17378	3.34427	-0.93493901
14784.5	3.23417	5.28388	3.23417	-0.96841512
15324.7	3.1258	5.39225	3.1258	-1.00249719
15883.9	3.00079	5.51726	3.00079	-1.04331185
16462.7	2.89205	5.626	2.89205	-1.08022185
17061.9	2.7867	5.73135	2.7867	-1.11732934
17682.1	2.67881	5.83924	2.67881	-1.15681478
18324.1	2.57885	5.9392	2.57885	-1.19484388
18988.6	2.46007	6.05798	2.46007	-1.24199764

19676.4	2.36493	6.15312	2.36493	-1.28143902
20388.4	2.25402	6.26403	2.25402	-1.32947215
21125.4	2.14594	6.37211	2.14594	-1.37860976
21888.3	2.0442	6.47385	2.0442	-1.42718093
22678	1.95171	6.56634	1.95171	-1.47348153
23495.4	1.85414	6.66391	1.85414	-1.52476646
24341.5	1.74405	6.774	1.74405	-1.58597745
25217.4	1.66214	6.85591	1.66214	-1.63408151
26123.9	1.56282	6.95523	1.56282	-1.69569556
27062.3	1.46702	7.05103	1.46702	-1.75895431
28033.7	1.38932	7.12873	1.38932	-1.81337302
29039.2	1.2929	7.22515	1.2929	-1.88529968
30080	1.21858	7.29947	1.21858	-1.94450119
31157.3	1.14279	7.37526	1.14279	-2.0087148
32272.5	1.06556	7.45249	1.06556	-2.07868696
33426.8	0.993033	7.525017	0.993033	-2.14917882
34621.6	0.912478	7.605572	0.912478	-2.23377874
35858.4	0.8478	7.67025	0.8478	-2.30729796
37138.7	0.778338	7.739712	0.778338	-2.39278184
38463.9	0.737552	7.780498	0.737552	-2.44660613
39835.6	0.658337	7.859713	0.658337	-2.56022576
41255.5	0.606175	7.911875	0.606175	-2.642774
42725.3	0.54653	7.97152	0.54653	-2.74635352
44246.6	0.50454	8.01351	0.50454	-2.8262956
45821.4	0.453989	8.064061	0.453989	-2.93186975
47451.5	0.419564	8.098486	0.419564	-3.01072664

49138.8	0.384942	8.133108	0.384942	-3.09685005
50885.4	0.354086	8.163964	0.354086	-3.1804029
52693.3	0.324265	8.193785	0.324265	-3.26838164
54564.7	0.286092	8.231958	0.286092	-3.39362928
56501.9	0.252356	8.265694	0.252356	-3.51910193
58507	0.216646	8.301404	0.216646	-3.67167804
60582.5	0.191522	8.326528	0.191522	-3.79494004
62731	0.168507	8.349543	0.168507	-3.92296543
64954.8	0.162412	8.355638	0.162412	-3.9598064
67256.8	0.154264	8.363786	0.154264	-4.0112773
69639.6	0.141072	8.376978	0.141072	-4.10067232
72106.1	0.116545	8.401505	0.116545	-4.29166526

Determination of $\Delta G^{\neq}_{rac(\mathrm{T})}$, $\Delta H^{\neq}_{rac}$ and $\Delta S^{\neq}_{rac}$ by dynamic HPLC

The racemization experiments were performed in a mixture of *n*-hexane:*i*-PrOH 99:1. In order to be consistent in the experiment, always the first obtained enantiomer from the chiral HPLC was used and the column temperature was always kept at 25 °C for all experiments. The kinetic data set was collected until the enantiomers reached a 55:45 ratio. We observed below the mentioned ratio an inaccuracy of the enantiomer fluctuation.

The rate of racemization between the enantiomers of **1** and **2** can be described as a first order process with **Equation 1**.

Equation 1 can be brought into a linear form:

$$ln\left(\frac{[A]_t}{[A]_0}\right) = -k_{rac}\, t \qquad \textbf{(2)}$$

$[A]_0$ being the enantiomeric excess at t = 0, $[A]_t$ the enantiomeric excess at the observed time t and k_{rac} the rate constant for the racemization.

The decay of the enantiomeric excess was determined by dynamic HPLC. The measurements were performed at four different temperatures (25 °C, 30 °C, 35 °C, 40 °C) and the resulting enantiomeric excess values are listed on the following tables.

Table S8: Racemization measurements of compound **1** at 25 °C.

**Measurement at 25 °C**				
time [s]	1st Band	2nd Band	$[A]_t$	$ln\left(\frac{[A]_t}{[A]_0}\right)$
0	99.4	0.6	98.8	0
3600	99.1	0.9	98.2	-0.00609139
7200	98.8	1.2	97.6	-0.01222011
10800	98.5	1.5	97	-0.01838663
14400	98.3	1.7	96.6	-0.02251886
18000	97.9	2.1	95.8	-0.03083492

21600	97.3	2.7	94.6	-0.04344013
25200	97.4	2.6	94.8	-0.0413282
28800	96.8	3.2	93.6	-0.05406722
32400	96.5	3.5	93	-0.06049811
36000	96.4	3.6	92.8	-0.06265096
39600	95.2	4.8	90.4	-0.08885334
43200	94.9	5.1	89.8	-0.09551263
46800	94.6	5.4	89.2	-0.10221657
50400	94.3	5.7	88.6	-0.10896575
54000	93.9	6.1	87.8	-0.1180361
57600	93.7	6.3	87.4	-0.12260232
61200	93.4	6.6	86.8	-0.12949098
64800	93.1	6.9	86.2	-0.13642743
68400	92.6	7.4	85.2	-0.14809617
72000	92.2	7.8	84.4	-0.1575302
75600	91.9	8.1	83.8	-0.1646646
79200	91.7	8.3	83.4	-0.1694493

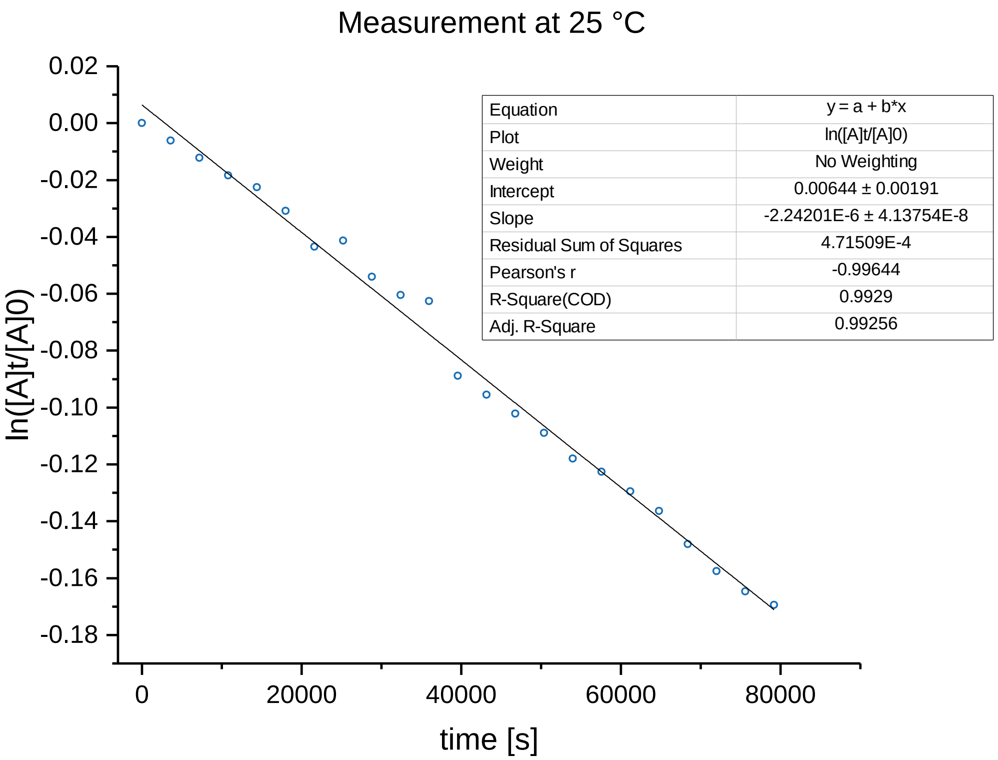

Figure S6: HPLC measurement of compound **1** at 25 °C and determination of k_{rac}.

Table S9: Racemization measurements of compound **1** at 30 °C.

**Measurement at 30 °C**				
time [s]	1st Band	2nd Band		$ln\left(\frac{[A]_t}{[A]_0}\right)$
0	98.7	1.3	97.4	0
7200	98.1	1.9	96.2	-0.0123969
10800	97.5	2.5	95	-0.0249493
14400	96.8	3.2	93.6	-0.0397958
18000	96.5	3.5	93	-0.0462267
21600	95.5	4.5	91	-0.0679667
25200	94.8	5.2	89.6	-0.0834709
28800	93.9	6.1	87.8	-0.1037647
32400	94.1	5.9	88.2	-0.0992192

36000	92.8	7.2	85.6	-0.1291409
39600	92.1	7.9	84.2	-0.1456313
43200	91.9	8.1	83.8	-0.1503932
46800	91.3	8.7	82.6	-0.1648165
50400	90.7	9.3	81.4	-0.1794509
54000	90	10	80	-0.1967996
57600	89.3	10.7	78.6	-0.2144545
61200	88.7	11.3	77.4	-0.2298394
64800	88.1	11.9	76.2	-0.2454647
68400	87.6	12.4	75.2	-0.258675
72000	87.2	12.8	74.4	-0.2693703
75600	86.5	13.5	73	-0.2883668
79200	86.1	13.9	72.2	-0.2993862
82800	85.7	14.3	71.4	-0.3105283

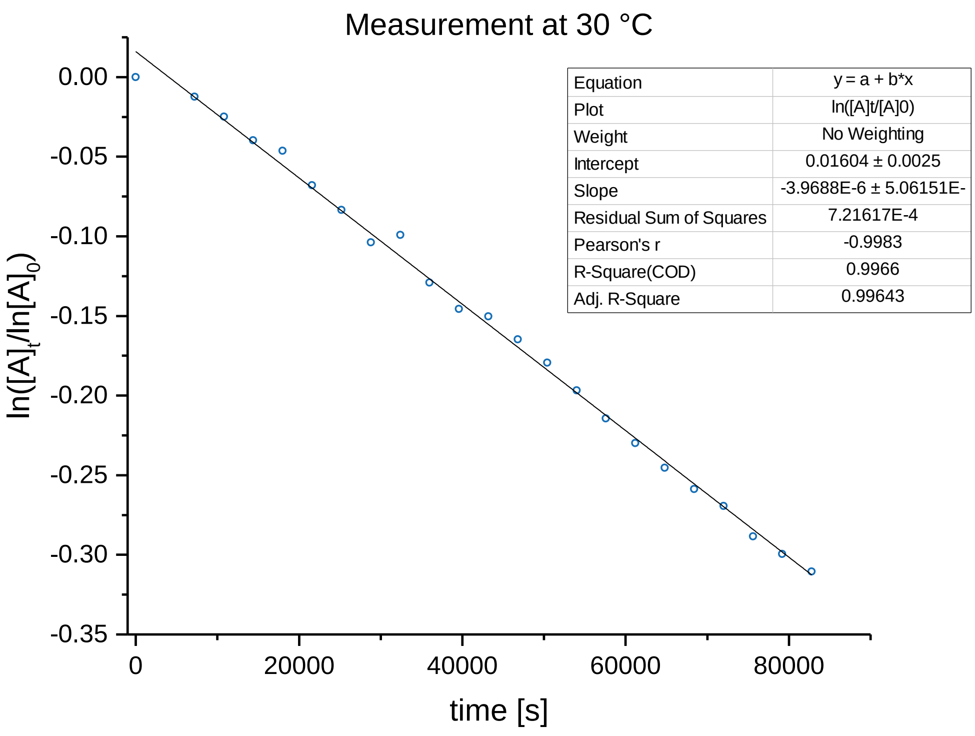

Figure S7: HPLC measurement of compound **1** at 30 °C and determination of k_{rac}.

Table S10: Racemization measurements of compound **1** at 35 °C.

**Measurement at 35 °C**				
time [s]	1st Band	2nd Band	$[A]_t$	$ln\left(\frac{[A]_t}{[A]_0}\right)$
0	98.1	1.9	96.2	0
3600	97.4	2.6	94.8	-0.0146599
7200	96.1	3.9	92.2	-0.0424692
10800	94.8	5.2	89.6	-0.071074
14400	93.7	6.3	87.4	-0.0959341
18000	92.4	7.6	84.8	-0.1261338
21600	91.1	8.9	82.2	-0.1572741
25200	90.1	9.9	80.2	-0.1819058
28800	88.8	11.2	77.6	-0.2148619

32400	88.7	11.3	77.4	-0.2174426
36000	86.4	13.6	72.8	-0.2787134
39600	84.6	15.4	69.2	-0.3294285
43200	83.6	16.4	67.2	-0.3587561
46800	82.5	17.5	65	-0.3920421
50400	81.6	18.4	63.2	-0.4201251
54000	81	19	62	-0.439295
57600	79.7	20.3	59.4	-0.4821351
61200	78.8	21.2	57.6	-0.5129068
64800	78.1	21.9	56.2	-0.5375126
68400	77.5	22.5	55	-0.5590962
72000	76.5	23.5	53	-0.5961374
75600	75.5	24.5	51	-0.6346037
79200	74.8	25.2	49.6	-0.6624385
82800	73.8	26.2	47.6	-0.7035966
86400	73	27	46	-0.737788
90000	72.4	27.6	44.8	-0.7642212
93600	71.6	28.4	43.2	-0.8005889

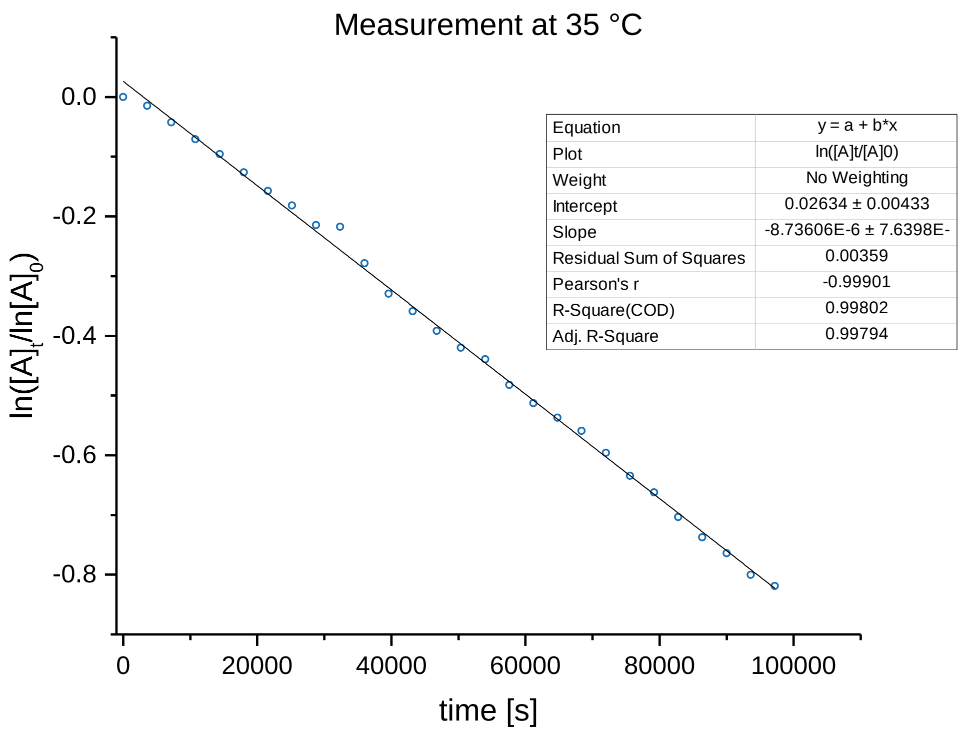

Figure S8: HPLC measurement of compound **1** at 35 °C and determination of k_{rac}.

Table S11: Racemization measurements of compound **1** at 40 °C.

**Measurement at 40 °C**				
time [s]	1st Band	2nd Band	$[A]_t$	$ln\left(\frac{[A]_t}{[A]_0}\right)$
0	96.4	3.6	92.8	0
1800	95.1	4.9	90.2	-0.0284172
3600	94	6	88	-0.0531098
5400	92.6	7.4	85.2	-0.0854452
7200	91.5	8.5	83	-0.111606
9000	90.5	9.5	81	-0.1359975
10800	89.2	10.8	78.4	-0.1686227
12600	88	12	76	-0.1997133
14400	86.9	13.1	73.8	-0.2290879

16200	85.9	14.1	71.8	-0.2565622
18000	84.8	15.2	69.6	-0.2876821
19800	83.6	16.4	67.2	-0.3227734
23400	82	18	64	-0.3715636
25200	80.9	19.1	61.8	-0.4065433
27000	80.2	19.8	60.4	-0.4294575
28800	79.2	20.8	58.4	-0.4631307
30600	78.4	21.6	56.8	-0.4909103
32400	77.5	22.5	55	-0.5231135
34200	76.7	23.3	53.4	-0.5526359
36000	75.9	24.1	51.8	-0.5830565
37800	75.2	24.8	50.4	-0.6104555
39600	74.9	25.1	49.8	-0.6224317
41400	73.8	26.2	47.6	-0.6676139
43200	73.1	26.9	46.2	-0.6974668
45000	72.5	27.5	45	-0.7237842
46800	71.9	28.1	43.8	-0.7508128
48600	71.2	28.8	42.4	-0.7832983

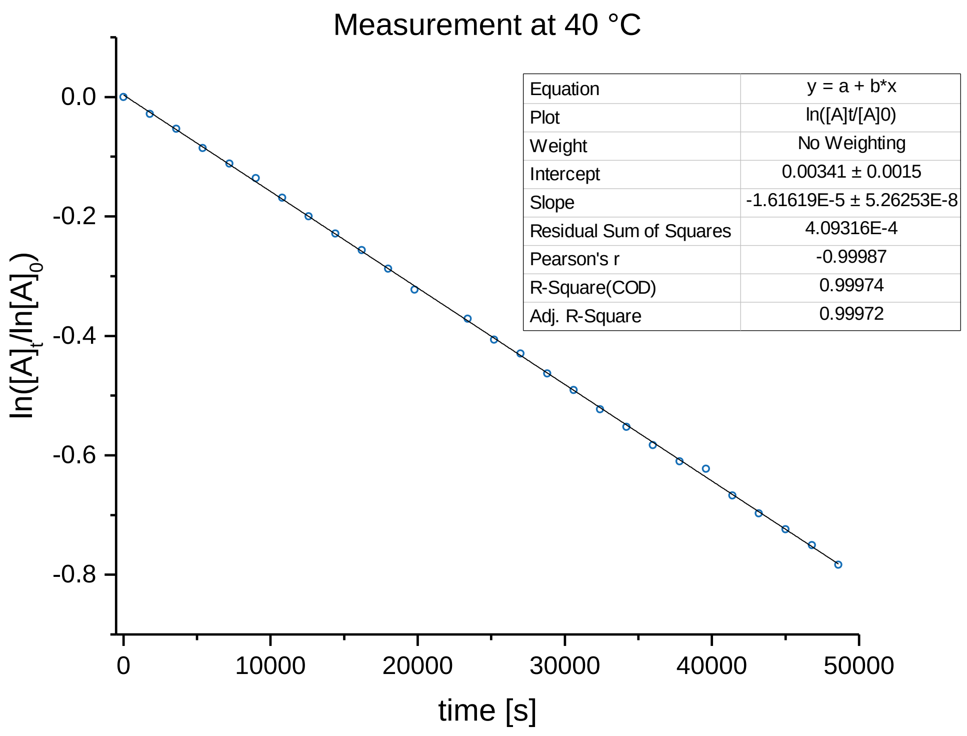

Figure S9: HPLC measurement of compound **1** at 40 °C and determination of k_{rac}.

Table S12: Racemization measurements of compound **2** at 25 °C.

**Measurement at 25 °C**				
time [s]	1st Band	2nd Band	$[A]_t$	$ln\left(\frac{[A]_t}{[A]_0}\right)$
0	96.1	3.9	92.2	0
1800	92.7	7.3	85.4	-0.07661403
3600	88.4	11.6	76.8	-0.18275549
5400	85.8	14.2	71.6	-0.25286506
7200	82	18	64	-0.36507705
9000	80	20	60	-0.42961557
10800	78.4	21.6	56.8	-0.4844238
12600	75.2	24.8	50.4	-0.60396896
14400	73.5	26.5	47	-0.67381253

16200	71.5	28.5	43	-0.76276001
18000	70.8	29.2	41.6	-0.79585996
19800	68.7	31.3	37.4	-0.90228943
21600	67.5	32.5	35	-0.96861207
23400	66.3	33.7	32.6	-1.03964784
25200	66.1	33.9	32.2	-1.05199368
27000	63.9	36.1	27.8	-1.19892411
28800	62.1	37.9	24.2	-1.3376075
30600	61.5	38.5	23	-1.38846591
32400	60.8	39.2	21.6	-1.45126682
34200	59.8	40.2	19.6	-1.54843056
36000	58.8	41.2	17.6	-1.65606123
37800	56.8	43.2	13.6	-1.91389034
39600	56.5	43.5	13	-1.95901077
41400	55.8	44.2	11.6	-2.07295503
43200	54.8	45.2	9.6	-2.26219703
45000	54.8	45.2	9.6	-2.26219703
46800	54.7	45.3	9.4	-2.28325044

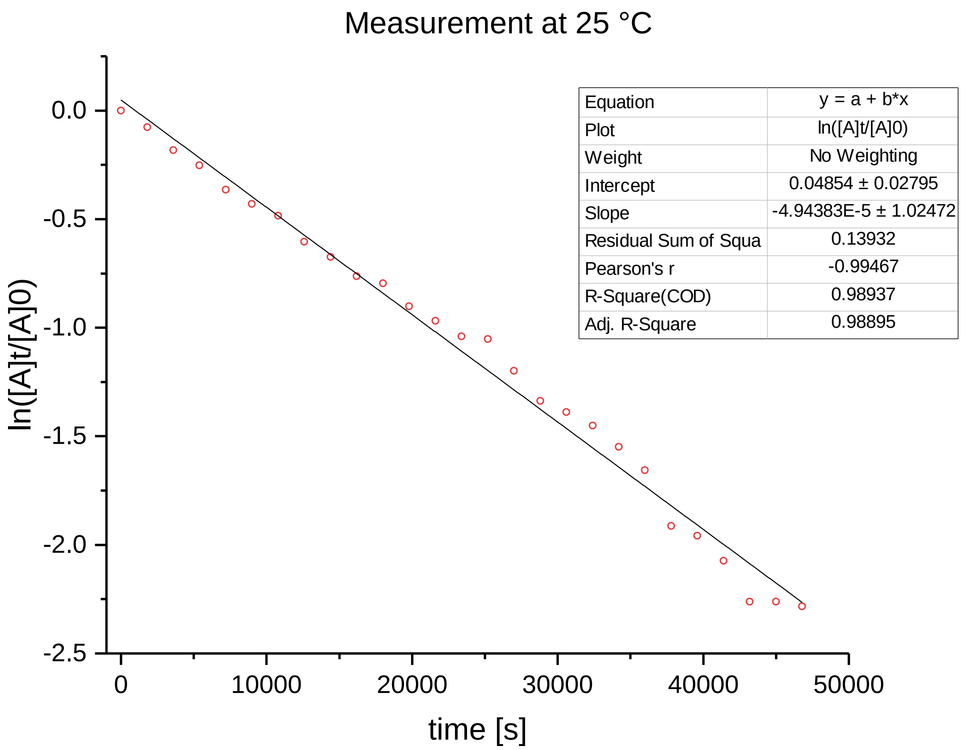

Figure S10: HPLC measurement of compound **2** at 25 °C and determination of k_{rac}.

Table S13: Racemization measurement of compound **2** at 30 °C.

**Measurement at 30 °C**				
time [s]	1st Band	2nd Band	$[A]_t$	$ln\left(\frac{[A]_t}{[A]_0}\right)$
0	99	1	98	0
1800	92.3	7.7	84.6	-0.14703321
3600	85.8	14.2	71.6	-0.3138724
5400	80.9	19.1	61.8	-0.46106411
7200	75.5	24.5	51	-0.65314185
9000	72.7	27.3	45.4	-0.76945537
10800	69.6	30.4	39.2	-0.91629073
12600	66.5	33.5	33	-1.08845992
14400	64.9	35.1	29.8	-1.19045909

16200	63.6	36.4	27.2	-1.28175051
18000	61.4	38.6	22.8	-1.45820694
19800	60.4	39.6	20.8	-1.55001449
21600	59.2	40.8	18.4	-1.67261681
23400	58.2	41.8	16.4	-1.78768614
25200	57.9	42.1	15.8	-1.82495754
27000	56.2	43.8	12.4	-2.06727101
28800	55.8	44.2	11.6	-2.13396238
30600	56.2	43.8	12.4	-2.06727101
32400	55	45	10	-2.28238239
34200	55.2	44.8	10.4	-2.24316167

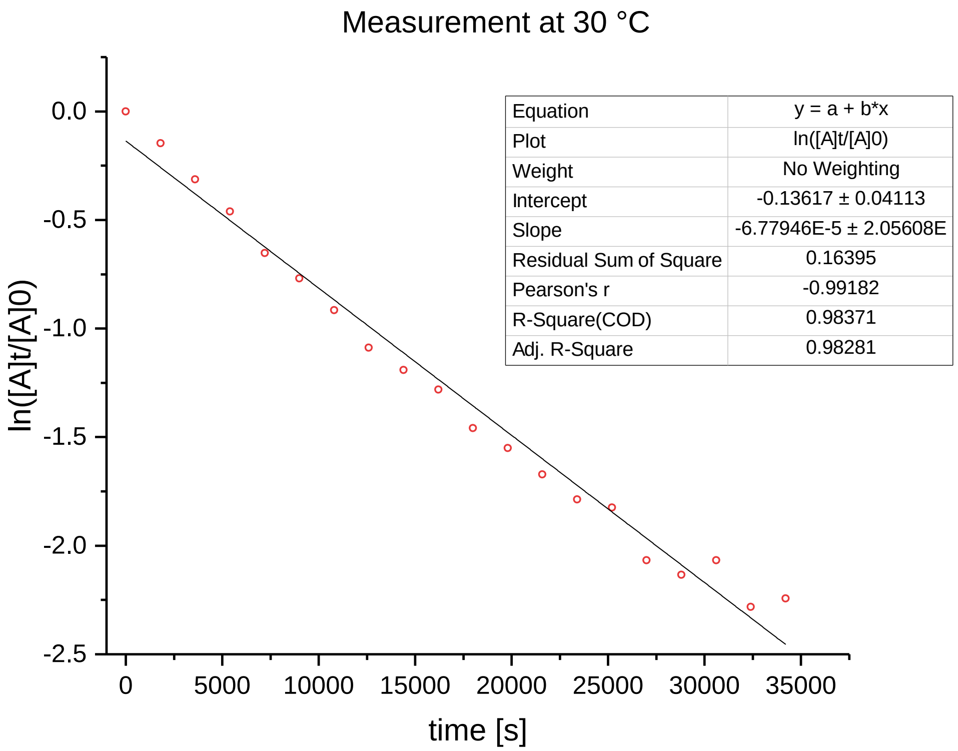

Figure S11: HPLC measurement of compound **2** at 30 °C and determination of k_{rac}.

Table S14: Racemization measurements of compound **2** at 35 °C.

**Measurement at 35 °C**				
time [s]	1st Band	2nd Band	$[A]_t$	$ln\left(\frac{[A]_t}{[A]_0}\right)$
0	100	0	100	0
1800	87.5	12.5	75	-0.28768207
3600	78.9	21.1	57.8	-0.54818141
5400	71.9	28.1	43.8	-0.82553637
7200	66.9	33.1	33.8	-1.08470938
9000	63.2	36.8	26.4	-1.33180618
10800	60.2	39.8	20.4	-1.58963529
12600	58.9	41.1	17.8	-1.72597173
14400	56.7	43.3	13.4	-2.00991548
16200	55	45	10	-2.30258509
18000	54.6	45.4	9.2	-2.3859667

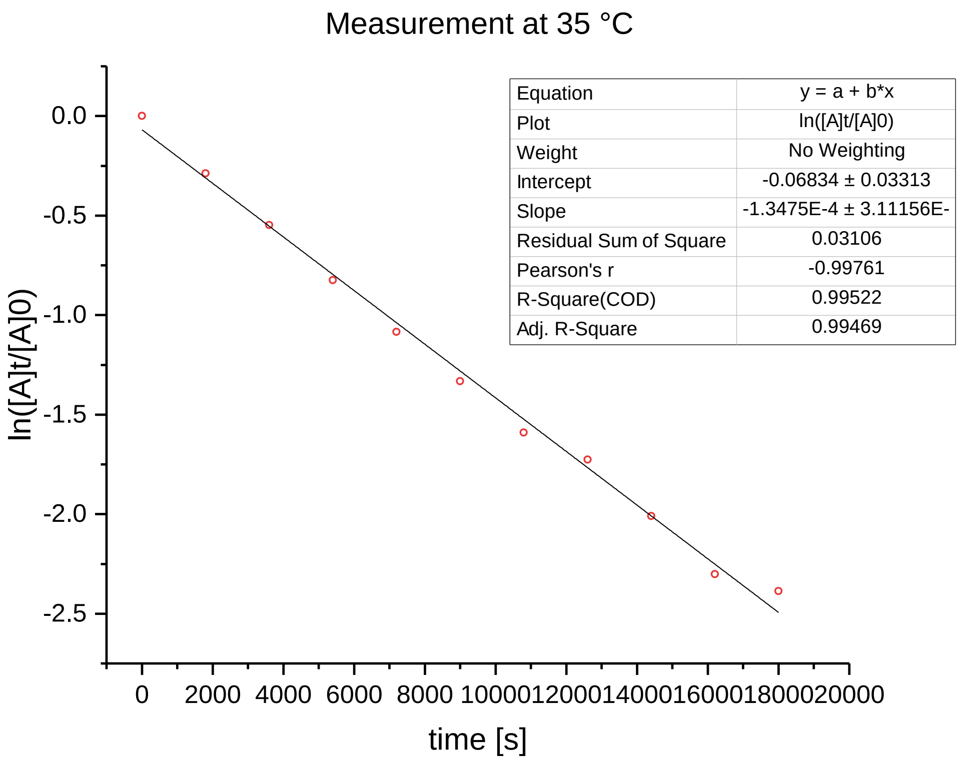

Figure S12: HPLC measurement of compound **2** at 35 °C and determination of k_{rac}.

Table S15: Racemization measurements of compound **2** at 40 °C.

**Measurement at 40 °C**				
time [s]	1st Band	2nd Band	$[A]_t$	$ln\left(\frac{[A]_t}{[A]_0}\right)$
0	100	0	100	0
1800	81.9	18.1	63.8	-0.449417
3600	71.1	28.9	42.2	-0.86274996
5400	64	36	28	-1.27296568
7200	59.1	40.9	18.2	-1.70374859
9000	57.5	42.5	15	-1.89711998
10800	56.9	43.1	13.8	-1.98050159
12600	54.2	45.8	8.4	-2.47693848
14400	54.5	45.5	9	-2.40794561

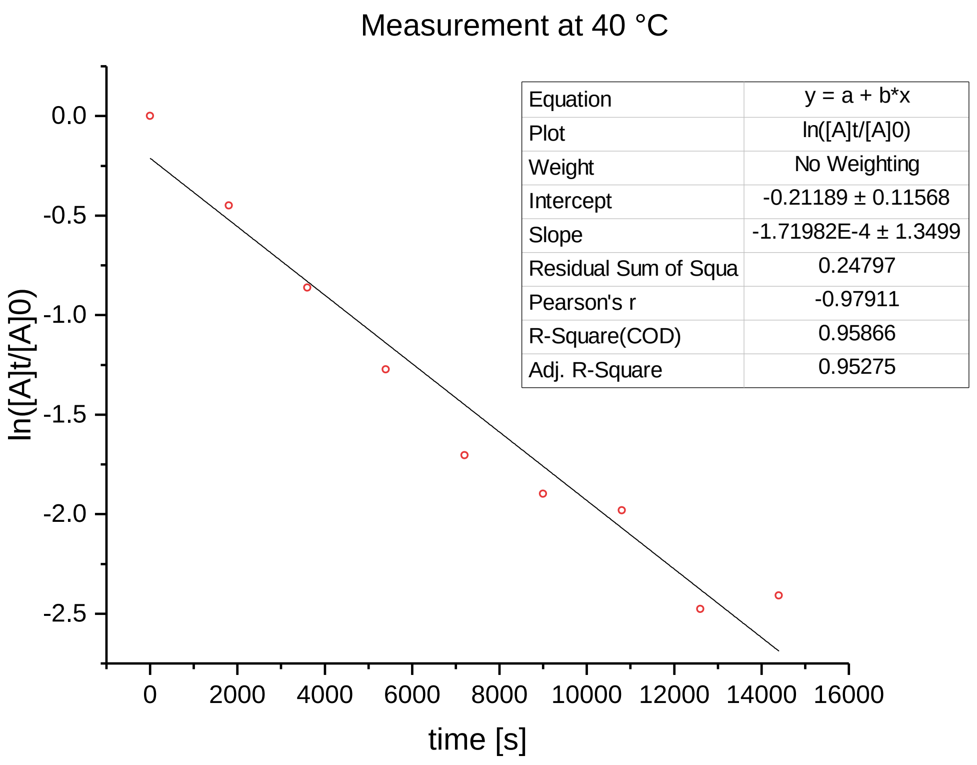

Figure S13: HPLC measurement of compound **2** at 40 °C and determination of k_{rac}.

Plotting t against $ln\left(\frac{[A]_t}{[A]_0}\right)$ gives a linear relation with the slope that corresponds to $-k_{rac}$.

Table S16: Rate constants and half-lifes of compound **1** and **2** at the different temperatures.

Temperature	Rate constant [1/s] of compound **1**		Half-life [h]		Rate constant [1/s] of compound **2**	Half-life [h]	
25 °C	k_{rac} =	2.24201E-06	τ =	85.88	4.94383E-05	τ =	3.89
30 °C	k_{rac} =	3.9688E-06	τ =	48.51	6.77946E-05	τ =	2.84
35 °C	k_{rac} =	8.74442E-06	τ =	22.02	0.00013475	τ =	1.43
40 °C	k_{rac} =	1.61619E-05	τ =	11.91	0.000171982	τ =	1.12

The k_{rac} values can be used to determine the free Gibbs energy of racemization $\Delta G^{\neq}_{rac(T)}$ by the rearranged Eyring equation:

$$\Delta G^{\neq}_{rac(298\,K)} = -RTln\left(\frac{hk_{rac}}{\kappa k_B T}\right) \quad \textbf{(4)}$$

Where k_{rac} is the obtained kinetic rate constant, κ the transition factor ($\kappa = 0.5$), k_B the Boltzmann constant (k_B = 1.380662 x 10^{-23} J K^{-1}), h the Planck's constant (h = 6.626176 x 10^{-34} J s), R the universal gas constant (R = 8.31446 J K^{-1} mol^{-1}) and T the measured temperature.

Table S17: Calculated values for the free Gibbs energy.

$\Delta G^{\neq}_{rac(T)}$[kJ/mol]	Compound **1**	Compound **2**
$\Delta G^{\neq}_{rac(298.15\,K)}$	103.6	95.9
$\Delta G^{\neq}_{rac(303.15\,K)}$	103.9	96.7
$\Delta G^{\neq}_{rac(308.15\,K)}$	103.6	96.6
$\Delta G^{\neq}_{rac(313.15\,K)}$	103.8	97.6

Using the Eyring equation

$$k_{rac} = \frac{\kappa k_B T}{h} e^{-\frac{\Delta G^{\neq}}{RT}} \qquad \textbf{(5)}$$

And substituting $\Delta G^{\neq}$ by

$$\Delta G^{\neq}_{(T)} = \Delta H^{\neq} - T\Delta S^{\neq} \qquad \textbf{(6)}$$

gives

$$k_{rac} = \frac{\kappa k_B T}{h} e^{\frac{-\Delta H^{\neq} + T\Delta S^{\neq}}{RT}} \qquad \textbf{(7)}$$

which can be brought in a linear form:

$$ln\left(\frac{k_{rac}}{T}\right) = -\left(\frac{\Delta H^{\neq}}{R}\right)\left(\frac{1}{T}\right) + \frac{\Delta S^{\neq}}{R} + ln\left(\frac{\kappa k_B}{h}\right) \qquad \textbf{(8)}$$

This equation states that plotting $ln\left(\frac{k_{rac}}{T}\right)$ vs. $\frac{1}{T}$ yield a straight line with slope = $-\left(\frac{\Delta H^{\neq}_{rac}}{R}\right)$ and intersect = $\frac{\Delta S^{\neq}_{rac}}{R} + ln\left(\frac{\kappa k_B}{h}\right)$.

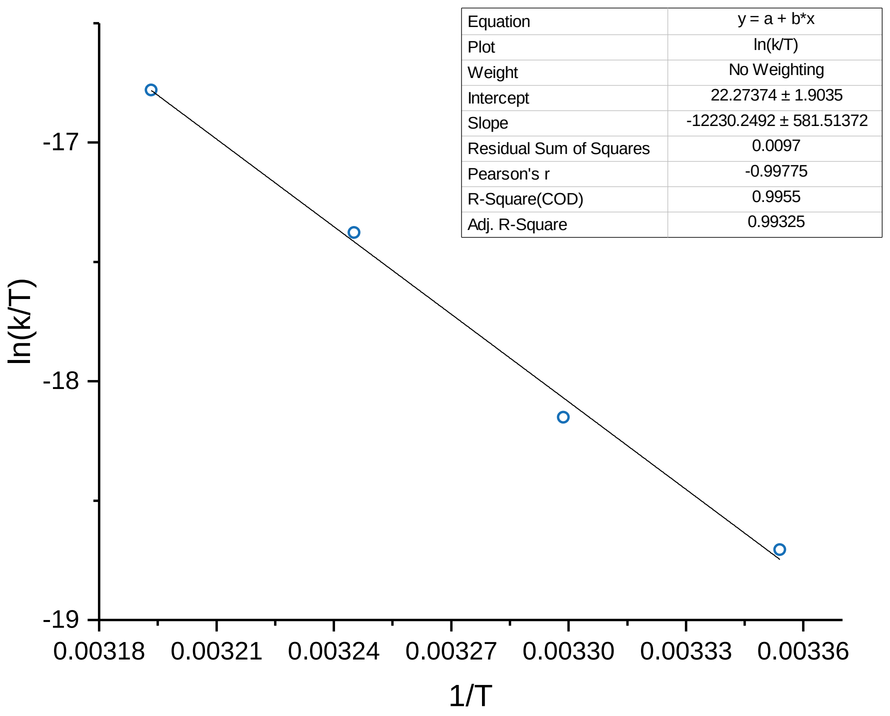

Figure S14: Eyring plot of compound **1**.

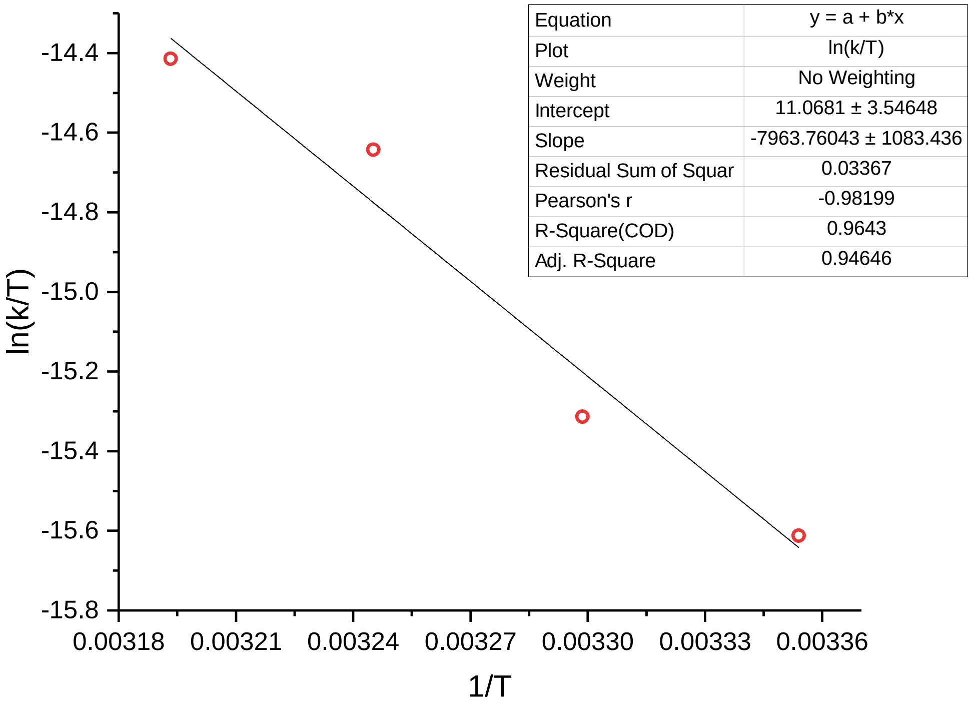

Figure S15: Eyring plot of compound **2**.

Table S18: The obtained $\Delta H^{\neq}_{rac}$ and $\Delta S^{\neq}_{rac}$ values for compound **1** and **2**.

	Compound **1**	Compound **2**
$\Delta H^{\neq}_{rac}$ [kJ/mol]	101.7	66.2
$\Delta S^{\neq}_{rac}$ [J/mol*K]	-6.6	-99.8

Table S19: Measured thermodynamic values of compound **3**-**7** by dynamic CD at 25 °C.

ratio of macrocycles	Compd.	Torsion angle [°]	$t_{1/2}$ [h]	$\Delta G^{\neq}_{rac}$ [kJ mol^{-1}]
[13:13]	**3**	147	3.82	95.8[b]
	**4**	178	1.49	93.5[b]
	**5**	159[a]	0.10	86.9[b]
[12:14]	**6**	126[a]	0.96	92.4[b]
	**7**	121	0.93	92.3[b]

[a] Computed values, [b] the κ factor was considered.
